# A Decision Support Tool Using an Open‐Source Methodology for Identifying Woody Encroachment and Juniper Species Vulnerability in the Chickasaw Nation, Oklahoma, USA

**DOI:** 10.1002/ece3.72551

**Published:** 2025-12-21

**Authors:** Mark Micozzi, Justin Baker

**Affiliations:** ^1^ Office of Natural Resources The Chickasaw Nation Ada Oklahoma USA; ^2^ Hazen and Sawyer Austin Texas USA

**Keywords:** Chickasaw Nation, conservation, Eastern Redcedar, Google earth engine, juniper, juniper evaluation tool, Oklahoma, remote sensing, vulnerability, woody encroachment

## Abstract

As a result of 20th century farming practices, fire suppression, grazing pressures, federal agriculture policies, drought, and related factors, juniper trees have experienced a dramatic increase in abundance across the Great Plains region, including millions of acres in Oklahoma. This invasive vegetation poses a significant threat to the environment, soil health, cultural resources and economies within the Cross Timbers region of the Chickasaw Nation in south‐central Oklahoma. To combat these negative impacts, the Chickasaw Nation (CN) has undertaken a proactive approach to evaluate the extent of juniper encroachment within the CN treaty territory (CNTT) and to use this information to make an economic case to landowners for the implementation of both prescribed burns, and juniper removal in general. This article describes the creation of a species‐focused evaluation methodology, model, and decision‐support tool, called the Juniper Evaluation Tool (JET), that is simple, open‐source, and applicable at an appropriate spatial scale to assist landowners, land managers, and CN resource managers in targeting best management practices and slowing the encroachment of juniper tree species. The JET, a product of a Natural Resources Conservation Service (NRCS) Conservation Innovation Grant (CIG), builds on the methods and functions of other planning tools, such as the Rangeland Analysis Platform (RAP) and the Rangeland Brush Estimation Toolbox (RaBET), but the outputs are tailored to parcel‐level management for watershed conservation planning in the CNTT. The JET aims to be a resource that is replicable, scalable, and actionable, and may provide the basis for an expanded tool that can be applied in other parts of Oklahoma and the Great Plains.

## Graphical abstract

First American peoples have relied on cultural burning to manage their lands and sustain healthy ecosystems, but modern agricultural practices and fire suppression have led to widespread juniper tree infestation in areas like Oklahoma. In response, the Chickasaw Nation developed an open‐source methodology, model and resulting decision‐support tool to evaluate the distribution of juniper species and the extent of juniper vulnerability, helping landowners, land managers and Chickasaw Nation resource managers choose the best management practices for their land. This initiative not only empowers local landowners and managers but may also have broader regional and national applications for the loss of grassland ecosystems at the expense of woody encroachment.

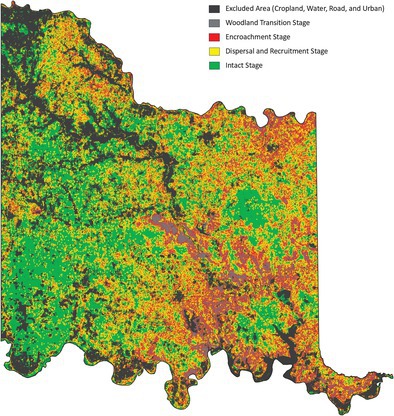

## 1 Juniper Expansion in Oklahoma

It is estimated that approximately 162 million hectares (400 million acres) of prairie grasslands in the Great Plains Region existed prior to European agriculture (Samson and Knoph 1994; Samson et al. 2004). Within these ecosystems, Junipers (*Juniperus* spp.), a pernicious yet traditionally native tree species, were primarily confined to protected alcoves, rocky outcrops and canyons as their distribution was limited by frequent fire regimes. However, as an increasing number of settlers made their way onto the plains, they inhibited the fires that had previously kept juniper species in check (Oklahoma Department of Agriculture, Food and Forestry 2002; DeSantis et al. 2025).

First American peoples long recognized that their livelihoods were dependent upon their tribal lands and waters as well as the abundant plant and animal communities supported by these invaluable resources. The prairies and grasslands, the river and stream flows, the springs and interstitial groundwaters are each inextricably linked to tribal identity, both in the native homelands along the rich bottomlands, forests, and ridges in the southeastern United States and today in the treaty territory occupied by the Chickasaw Nation (Chickasaw Nation and Choctaw Nation of Oklahoma 2024). Practicing age‐old stewardship and sustainability concepts, Indigenous people actively utilized “cultural burning” (i.e., the intentional lighting of small, controlled fires to provide a desired cultural service) (Roos 2020) to renew and maintain open grasslands, sustain plant species desirable to native populations, and establish hunting grounds with plentiful game (Fowler and Konopik 2007). Importantly, these healthy ecosystems and croplands, which became more resilient to destructive fire, yielded clothing and items essential to tribal ceremonies and related cultural activities. According to Frank Kanawha Lake, a research ecologist with the USDA Forest Service and a wildland firefighter of Karuk descent, “[Cultural burning] links back to the tribal philosophy of fire as medicine. When you prescribe it, you're getting the right dose to maintain the abundance of productivity of all ecosystem services to support the ecology in your culture” (Roos 2020).

**FIGURE 1:**
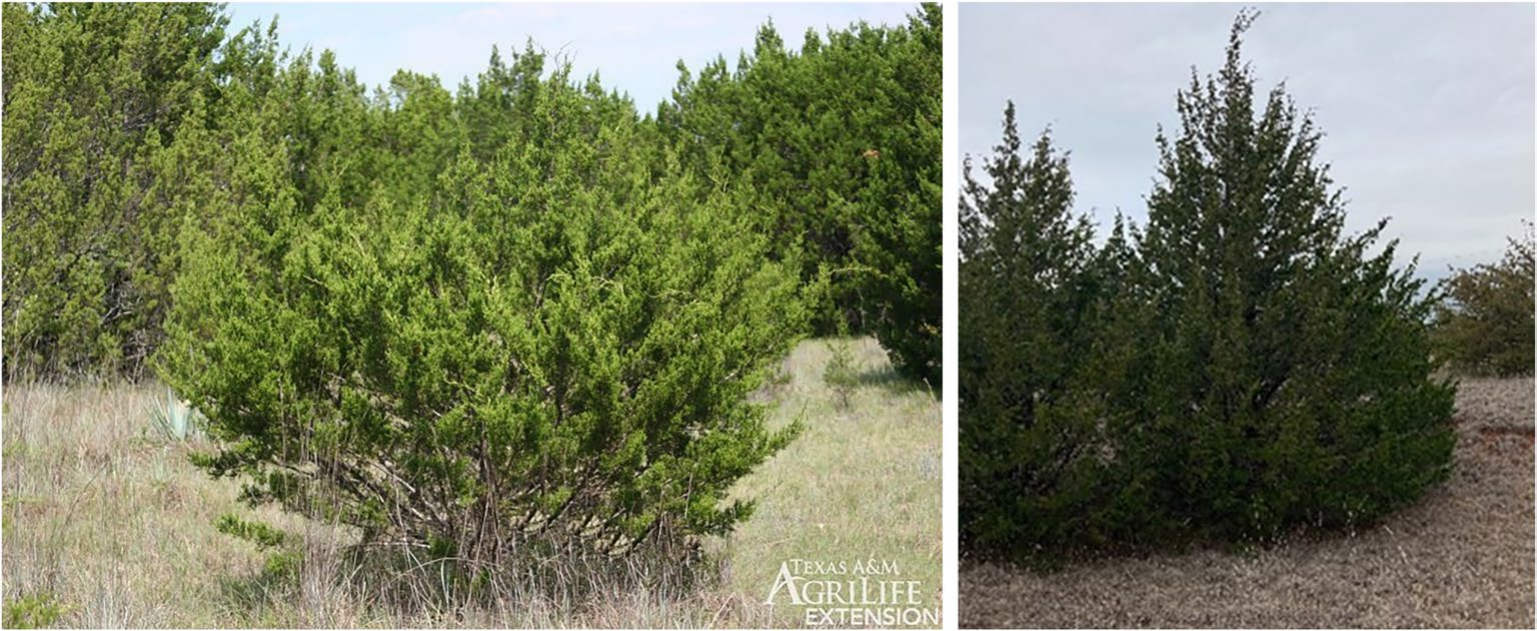
Ashe Juniper Tree (left) and Eastern Redcedar Tree (right). Ashe Juniper *Source:* https://www.beautifulhayscounty.org/resources/cedar‐trees‐understanding‐the‐role‐of‐the‐ashe‐juniper‐in‐central‐texas‐history‐and‐ecosystems? Eastern Red Cedar Source: Author‐provided photograph.

Today, because of 20th century farming practices, fire suppression, grazing pressures, federal agriculture policies, drought, and related factors, junipers have experienced a dramatic increase in abundance across the region (Van Auken 2009; DeSantis et al. 2011; Oklahoma Department of Wildlife Conservation 2015; Archer et al. 2017). This invasive vegetation poses a significant threat to the environment, soil health, cultural resources and economies within the Cross Timbers region of the Chickasaw Nation in south‐central Oklahoma. Species of particular concern include Eastern redcedar ( *Juniperus virginiana* ) and Ashe juniper ( *Juniperus ashei* ), both members of the Cupressaceae (Cypress) family. Due to the similarity of their detrimental impacts upon the native prairie landscapes of Oklahoma, they are hereafter collectively referred to as “juniper” or “juniper tree species” (Figure 1).

Throughout the region, both species readily outcompete established sun‐ and fire‐tolerant species of mainly post oak ( *Quercus stellata* ) and blackjack oak ( *Quercus marilandica* ) (DeSantis, Hallgren, Lynch, et al. 2010; DeSantis et al. 2025). From 1994 to 2001, it was estimated that areas infested with juniper tree species that contained at least 50 trees per acre increased from 6.3 to 8 million acres (Oklahoma Department of Agriculture, Food and Forestry 2002) and by 2013, this number would grow to 12.6 million acres, creating a significant loss of native prairies, shrublands, cross timbers forests and other ecosystems (Bidwell and Moseley 1989; Coffey 2013). In Oklahoma, including the Chickasaw Nation, the rapid spread of juniper tree species is considered the most important natural resource concern (Bidwell et al. 2000; Oklahoma Department of Agriculture, Food and Forestry 2002) and considerable research has been published on the subject (Bidwell et al. 2000; Coppedge et al. 2001; Bidwell et al. 2002; Zhang and Hiziroglu 2010; Zou et al. 2015; Hoff, Will, Zou, and Lillie 2018; Wang et al. 2018).

## 2 Impacts of Juniper Encroachment

This unprecedented expansion and infestation of juniper tree species has been identified as a major problem for ecosystems in the Great Plains, including Oklahoma (Bidwell et al. 2000; Van Auken 2000; Briggs et al. 2002; Briggs et al. 2005; Oklahoma Department of Agriculture, Food and Forestry 2002; Meneguzzo and Liknes 2015; Hoff, Will, Zou, and Lillie 2018; Fogarty et al. 2020; Ueckert 2020; Twidwell et al. 2021). Estimates of economic loss in Oklahoma from juniper encroachment in 2002 from cattle forage, lease hunting opportunities, increased wildfire risk, recreation, and water yield were estimated at $218 million and were projected to reach almost half a billion dollars in 2013, stressing the need for juniper management to protect both ecological integrity and economic productivity (Oklahoma Department of Agriculture, Food and Forestry 2002; Oklahoma Conservation Commission 2008; Zhang and Hiziroglu 2010). Increased land management costs from low encroachment maintenance to high encroachment restoration stress the importance of ongoing maintenance (Bidwell et al. 2002).

Loss and fragmentation of wildlife are fundamentally tied to a declining quantity and quality of habitat, stemming from pervasive land‐use conversion, fragmentation, altered disturbance regimes, and the proliferation of invasive species, including juniper species (Bidwell et al. 2000; Fuhlendorf et al. 2012). In addition, biodiversity loss with increased woody plant encroachment typically manifests through a marked decline in plant species richness, with greater losses occurring in wetter regions and where net primary productivity shifts significantly toward woody biomass (Ratajczak et al. 2012). For example, in tallgrass prairies invaded by junipers, herbaceous species richness dropped significantly under closed canopy junipers, with little to no herbaceous productivity, resulting in the near elimination of native grassland species (Briggs et al. 2002; Knapp et al. 2008; Limb et al. 2010). Bird communities are similarly affected; more than 70% of grassland bird species have experienced population declines in the Great Plains as their habitats are overtaken by woody vegetation, especially eastern redcedar (Engle et al. 2008; Twidwell et al. 2013; Rosenberg et al. 2019; North American Bird Conservation Initiative 2025). Animal diversity peaks at low to intermediate woody cover but then declines sharply as woody plants become dominant, displacing grassland specialists and shifting the community structure to favor shrub‐ or woodland‐associated species. In certain cases, encroachment by a few woody species led to monocultures that caused profound reductions in both plant and animal diversity (Archer et al. 2017).

Encroachment significantly affects yield gaps in herbaceous production and decreases agricultural productivity, suppressing the growth and regeneration of productive forage species, and by extension, livestock (Bidwell et al. 2000; Briggs et al. 2005; Anadón et al. 2014; Morford et al. 2022). This loss is initially localized but grows more severe as tree density and canopy closure increase (Engle et al. 1987). The transformation can be stark as originally diverse and productive rangeland transitions into areas dominated by unpalatable woody species, making it challenging for land managers to maintain herd sizes, manage weight gain, leading to the need to purchase supplemental feed, all contributing to reduced agricultural profitability (Taylor Jr et al. 2012; Anadón et al. 2014; Morford et al. 2022). Beyond livestock productivity, the loss in agricultural productivity associated with woody encroachment also translates to reduced land value, according to the Oklahoma Conservation Commission (2008).

Increased woody encroachment, particularly by juniper species, also leads to a substantial rise in wildfire potential by the introduction of highly flammable materials, dramatically altering the fuel structure of the landscape (Zhang and Hiziroglu 2010; Hoff, Will, Zou, Weir, et al. 2018; Donovan et al. 2023). Volatile oils ignite easily and contribute to rapid fire spread, while juniper foliage and small branches are especially prone to carrying flames vertically (i.e., “ladder fuels”) into the canopy, increasing the risk of intense crown fires rather than only surface fires. This volatility makes regions with dense encroachment much more hazardous during wildfire events, as even moderate wildfires can become severe, fast‐moving crown fires that are difficult to control and dangerous to suppress. This effect is particularly critical in areas where encroachment has shifted the plant community from an open grassland or savanna to dense woodlands or forests, as has been observed in parts of Oklahoma and Nebraska, as well as throughout the Great Plains and Cross Timbers regions (Briggs et al. 2005; Zhang and Hiziroglu 2010; Hoff, Will, Zou, Weir, et al. 2018; Donovan et al. 2023).

In terms of soil composition and carbon sequestration, encroachment‐driven changes include greater spatial variability in soil carbon, shifts in the soil microclimate that slow decomposition and nutrient cycling, a decline in native grass root systems, buildup of undecomposed organic material that may inhibit understory growth, and possible alterations in the stability and resilience of stored carbon (Norris et al. 2001; Nunes Biral et al. 2019). Woody plant encroachment also poses serious, well‐documented threats to both water quantity (streamflow, groundwater recharge, and runoff) and water quality (dilution capacity, sediment, and nutrient dynamics) across a range of ecosystems. These impacts are multifaceted, affecting surface and subsurface hydrology, ecosystem processes, and the sustainability of municipal and agricultural water supplies (Huang et al. 2006; Caterina et al. 2013; Zou et al. 2013, 2017, 2018; Zou and Engle 2017; Yang, Winrich, et al. 2024). Lastly, human health effects from airborne pollen in the form of allergic reactions and possible asthma and other respiratory effects are directly associated with juniper encroachment (Van De Water and Levetin 2001).

## 3 Juniper Species Identification Using Remotely Sensed Data

Over the past few decades, remote sensing has been increasingly used in a wide variety of contexts, scales and applications to identify and quantify changes in land cover, including applications in rangeland and forestry management (Booth and Tueller 2003; Hartfield and Van Leeuwen 2018; Galgamuwa et al. 2020). These applications have included monitoring the expansion of invasive species (Chance et al. 2016; Dronova et al. 2017; Hagani et al. 2023; Singh et al. 2024; Rakotoarivony et al. 2025) as well as native species that have exhibited invasive‐like behavior (i.e., native invasives) due to their negative encroachment effects (i.e., woody plant encroachment, WPE), including juniper tree species (Msanne et al. 2017; Wickramarathna et al. 2021; Morford et al. 2022; Yang, Will, et al. 2024). Remote sensing techniques have several advantages, including repeatability, the ability to implement on a large scale, and lower costs than field‐based surveys (Booth and Tueller 2003; Mirik et al. 2013). Field surveys have been used to quantify changes in the distribution of juniper species, particularly eastern redcedar, over time (Kaur et al. 2020), but these have been limited in scale and are difficult to repeat due to high costs and labor needs for larger areas (Meneguzzo and Liknes 2015) as well as the inaccessibility of some areas and observer bias (Mirik et al. 2013).

To date, there have been many studies on quantifying various aspects of juniper spread using very high resolution (VHR) imagery (Wang et al. 2021), hyperspectral imagery (Wylie et al. 2000), National Agricultural Imagery Program (NAIP) aerial photography (Mirik et al. 2013), historical aerial photography (Middleton and Norman 2021; Léger and Katz 2022), Light Detection and Ranging (LiDAR) (Wang et al. 2021), and unmanned aerial vehicle (UAV) derived data (Wang et al. 2021). Many of these previous studies have taken advantage of the fact that juniper/conifer trees are evergreen species, which have stable spectral signatures, and can therefore be distinguished from other vegetation in winter, leaf‐off images relatively easily, depending on the location and presence of other deciduous trees (Hoff, Will, Zou, Weir, et al. 2018; Galgamuwa et al. 2020). LiDAR data have been used in many contexts to quantify tree canopy and biomass (Ghanbari Parmehr and Amati 2021; Henn and Peduzzi 2023; Jakubowski et al. 2013), including in conjunction with other data inputs, such as unmanned aircraft systems imagery (Wang et al. 2021), hyperspectral data (Zhang and Fang 2012) and satellite imagery (Sankey et al. 2010; Jakubowski et al. 2013). Synthetic aperture radar combined with satellite imagery has also been used to successfully quantify tree canopy (Qin et al. 2016; Wang et al. 2017, 2018; Yao et al. 2025).

While there have been previous studies on the distribution of juniper species across broad areas using remote sensing datasets, including Landsat data (Sankey and Germino 2008; Wang et al. 2017, 2018; Kaskie et al. 2019; Yang et al. 2021), these studies were carried out at moderate to low spatial resolutions and aligned to regionally based objectives. The use of higher spatial resolution NAIP aerial photography, combined with wavelet analysis and/or object‐based image analysis, has been very successful in mapping woody encroachment of individual trees and open woodlands at local and regional scales (Nielsen and Noone 2014; Poznanovic et al. 2014; Falkowski et al. 2017; Gustafson et al. 2018; Roth et al. 2021), in particular to meet the specific needs of the threatened sage grouse in the western United States (Miller et al. 2017). Although higher resolution datasets (i.e., aerial photography and satellite imagery) are a preferred option to map woody encroachment in many cases, they can be cost‐prohibitive at regional extents, are not always publicly available, are difficult for landowners and land managers to obtain and use, require a more highly trained staff with increased workloads, and contain computational limits (Roth et al. 2021).

### 3.1 Purpose

With the spread of invasive juniper species showing no indication of slowing down (Yang, Will, et al. 2024) and its negative impacts on management costs, species diversity, wildfire risk, water resources, agricultural productivity, and soil health, the CN is taking proactive steps to mitigate these growing problems and their detrimental impacts across the treaty territory by encouraging the development and implementation of BMPs, including brush control using prescribed burns, riparian buffer zone establishment, rotational grazing, and minimal to no‐till farming to address water quantity and quality concerns, ecosystem health, habitat restoration, and soil health issues. Most recently, the CN was awarded a NRCS CIG to evaluate the distribution and extent of juniper species infestation and vulnerability within its treaty territory. Given that juniper cover is projected to double every 18 years (Oklahoma Department of Agriculture, Food and Forestry 2002) and recognizing the urgent need for effective intervention, the CN sought to develop a simple, open‐source, actionable and publicly accessible tool designed to empower landowners, land managers, and CN resource managers with data at a management‐relevant scale. The Juniper Evaluation Tool (JET) is specifically developed to address a critical gap, as existing large‐scale wood encroachment platforms (Table 1), while valuable, lack the species‐level specificity or regional focus required for effective management in Oklahoma (see Open‐Source Woody Encroachment Web‐Based Tools in the Supporting Information for a detailed review of each tool with appropriate citations). The purpose of this article, therefore, is to describe the remote sensing‐based methodology, model, and decision support tool (i.e., the JET) developed by the CN to equip landowners, land managers and CN resource managers with an actionable resource which can be used to accelerate planning and counter the rapid spread of invasive woody species.

**TABLE 1:** Comparison of open‐source woody encroachment web‐based tools (see Open‐Source Woody Encroachment Web‐Based Tools in the Supporting Information for a detailed review of each tool with appropriate citations).

Tool	Year created/Launched	Data source and resolution	Purpose	Strengths
Rangeland Analysis Platform (RAP)	2018	Field‐collected data; 30 m Landsat satellite imagery; cloud computing	Provide a free, user‐friendly, science‐based, near‐real‐time geospatial tool for assessing trends in rangeland vegetation cover and production (annual/perennial forbs & grasses, shrubs, trees, bare ground) from 1985–present	Long‐term, contiguous U.S. coverage; integrates field data; near‐real‐time updates; multicomponent vegetation monitoring; accessible and free
Sage Grouse Initiative (SGI) Interactive Web Application	2016	High‐resolution NAIP aerial photography (1 m); object‐based image analysis; automated feature extraction; validated with reference imagery and ground surveys	Map pinyon‐juniper presence, distribution, and canopy cover; integrate ecosystem resilience/resistance indices to support sage‐grouse habitat conservation across 11 western U.S. states	Very high spatial resolution (1 m); highly accurate classification; maps both individual trees and woodland canopies; robust validation; scalable and replicable for targeted conservation
Multi‐Resolution Land Characteristics (MRLC) Consortium Tools—NLCD & RCMAP	NLCD: 2001; RCMAP:2021	NLCD: Landsat & Sentinel‐2 (30 m) + USDA Forest Service FIA data; RCMAP: Extensive field data + Landsat (30 m); neural network models	NLCD: Track nationwide land cover, land cover change, tree canopy RCMAP: Monitor, assess, and project rangeland condition over time and under various climate scenarios	Consistent, nationwide datasets; long‐term time series back to 1985; field‐validated; supports multiple spatial/temporal scales; free and open access; advanced modeling for fractional cover
Rangeland Brush Estimation Tool (RaBET)	2023	30 m Landsat imagery + high‐resolution NAIP aerial photography (1 m) + ground measurements	Quantify woody plant encroachment; evaluate brush density and historical trends; monitor management effectiveness across western U.S. rangelands	Integrates multiple data sources; repeatable, field‐scale estimates; supports monitoring and adaptive management; free and public; facilitates targeted conservation and restoration

### 3.2 Study Area

**FIGURE 2:**
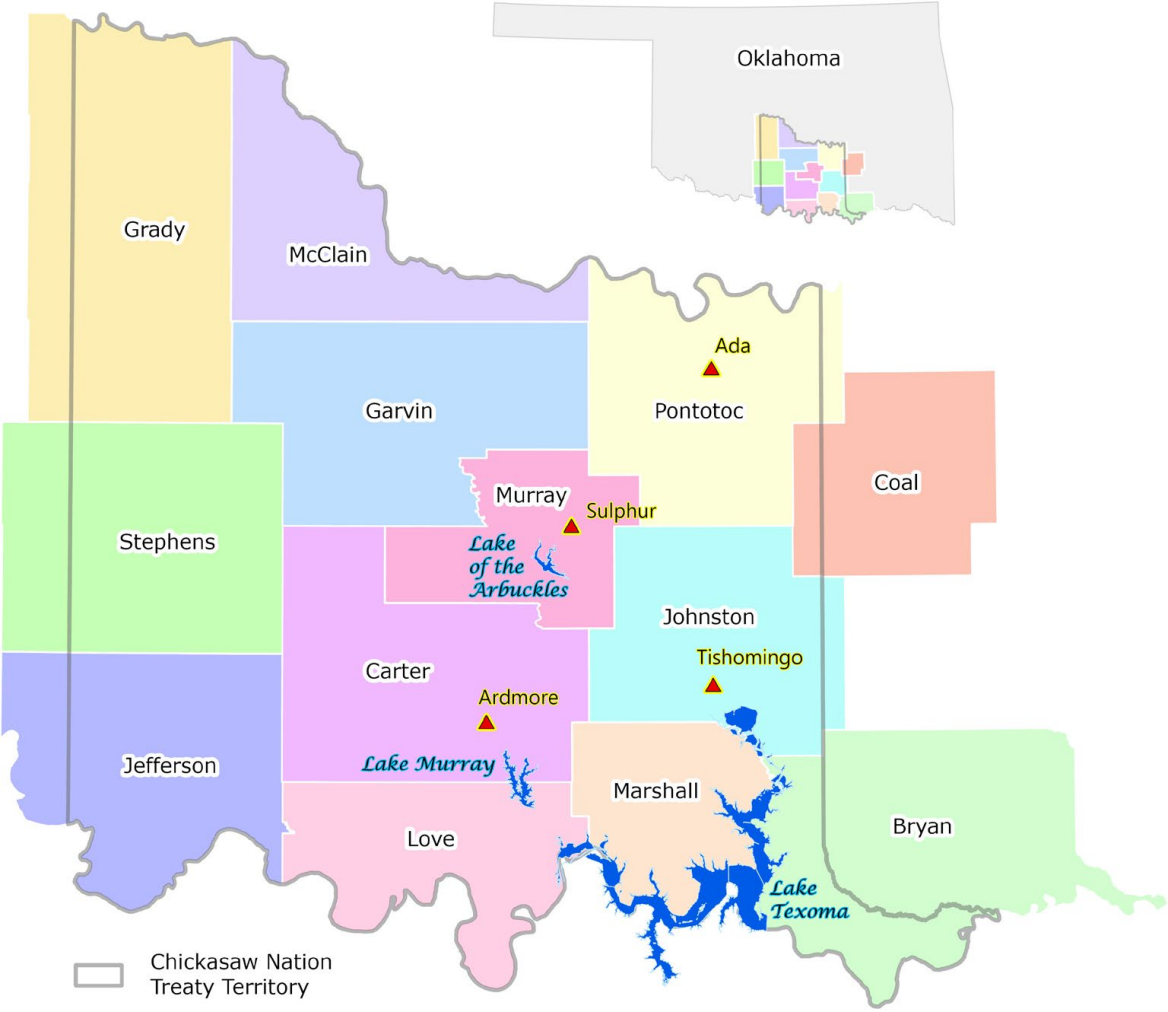
The Chickasaw Nation Treaty Territory and noteworthy towns and lakes. Tribal jurisdictional boundaries can be found on the Bureau of Indian Affairs website at https://onemap‐bia‐geospatial.hub.arcgis.com.

The CNTT encompasses all or part of 13 counties within south‐central Oklahoma (Figure 2), covering approximately 7648 mile^2^ (4.9 million acres) of the Central Great Plains and Cross Timbers Ecoregion (see Study Area in the Supporting Information for an accompanying map). Land use within the CNTT is primarily agricultural, though there are several municipal areas, including Ada, Ardmore, Sulfur, and Tishomingo. There are also several large water bodies, including the Lake of the Arbuckles, Lake Murray, and a large portion of Lake Texoma in the CNTT. However, approximately 80% of the land cover within the CN is made up of Grass/Pasture (approx. 53%) and Deciduous Forest (approx. 28%), with most of the cropping agriculture found in the western portion of the CNTT. In the eastern portion, most agriculture consists of cow‐calf operations and ranching (see Study Area in the Supporting Information for an accompanying table and map).

## 4 Methodology

### 4.1 Juniper Tree Species Distribution Modeling

**FIGURE 3:**
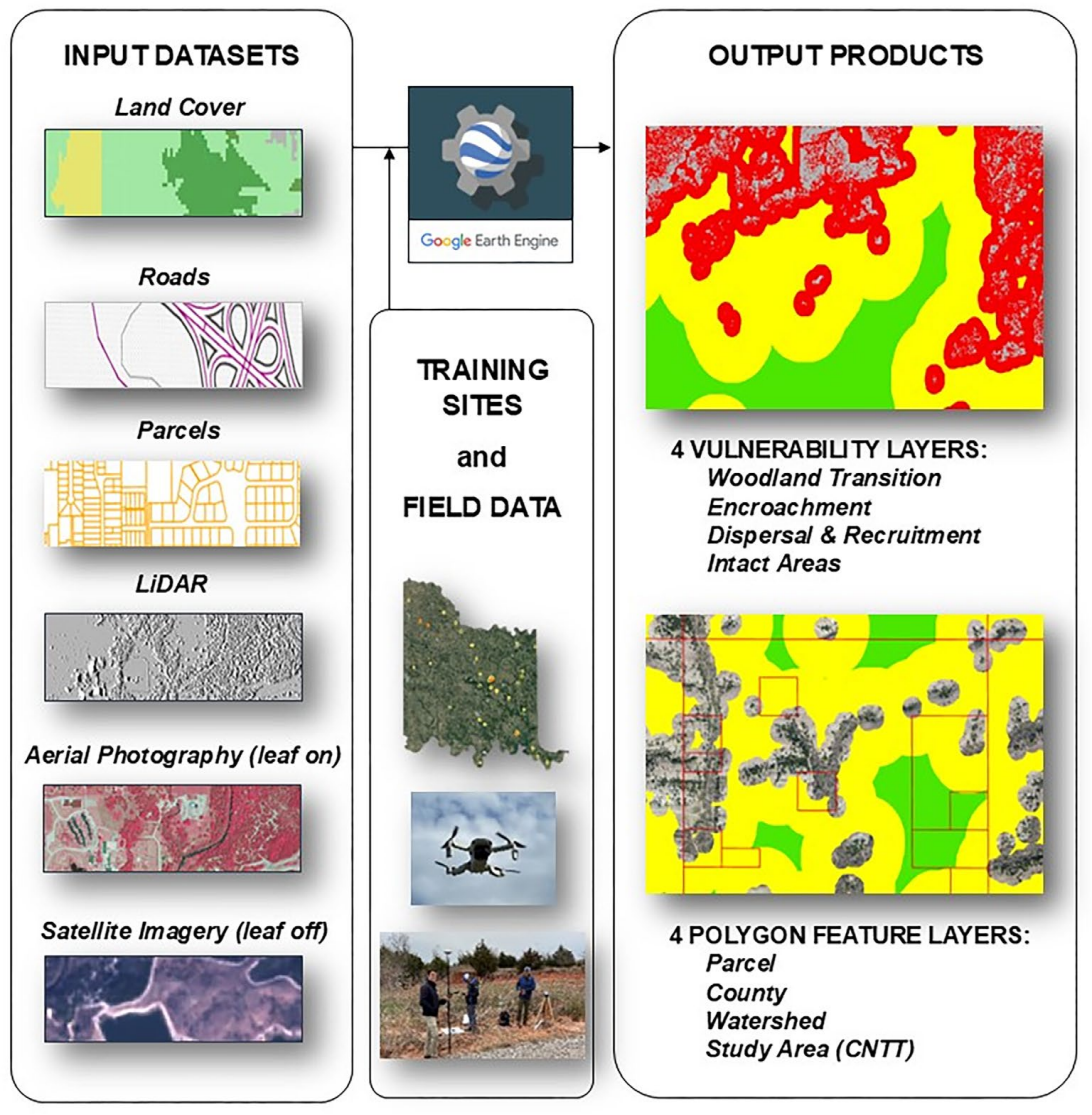
Juniper tree species distribution model flow diagram.

The general approach of the juniper tree species distribution model involved using three distinct, open‐source remote sensing datasets, described below and shown in Table 2, with a flow diagram shown in Figure 3.

1. LiDAR‐based elevation data (Oklahoma Geographic Information Council, OKGIC, and United States Geologic Survey, USGS; various dates). 1‐band, 1‐m resolution. This dataset allows identification of tree canopy, though other nontree features are included.
2. National Agriculture Imagery Program, NAIP, Aerial Photography (United States Department of Agriculture, USDA; 2021). 4‐band, 1‐m resolution. This dataset allows identification of vegetation, generally, and the tree canopy within the study area, in particular.
3. Sentinel‐2 satellite imagery (European Space Agency, ESA; various dates in 2020–2022). 4‐band, 10‐m resolution. This dataset allows identification of juniper species by isolating areas of photosynthetically active vegetation during winter months.

**TABLE 2:** Data sources used in the Juniper Tree Species Distribution Model (stepwise order from start to finish). Specific dataset source locations are provided in the separate sections of the methodology.

Dataset	Source	Resolution	Band(s)	Date(s) collected	Description/Use
Chickasaw Nation Treaty Territory	Chickasaw Nation Geospatial Information Department	N/A	N/A	N/A	Study area boundary
Oklahoma Ecological System Mapping (OKESM) Land Cover	Oklahoma Department of Wildlife Conservation	10‐m	N/A	2012–2015	Exclude water areas and crop land
ESRI Sentinel‐2 Land Cover	ESRI, derived from Sentinel‐2 Imagery	10‐m	N/A	2022	Exclude water areas and crop land
Oklahoma Roadway Lines	Oklahoma Department of Transportation	N/A	N/A	Last updated in 2023	Exclude roads
Oklahoma Municipal Areas	Oklahoma Geographic Information Council	N/A	N/A	Last updated in 2021	Exclude municipal parcels less than 1‐acre in size
Oklahoma Parcels	Visual Lease Services Inc	N/A	N/A	Updated in 2022	Exclude municipal parcels less than 1‐acre in size
LiDAR	Oklahoma Geographic Information Council & USGS Lidar Explorer	1‐m	N/A	Various (2009–2018)	Exclude flat, nonforest areas
NAIP aerial photography	USDA from GEE	1‐m	R, G, B, NIR	2021 (Summer)	Exclude nonphotosynthetically active vegetation. Data product: Tree cover layer (1‐m resolution)
Sentinel‐2 satellite imagery	European Space Agency from GEE	10‐m	R, G, B, NIR	Various 2020–2022 (winter)	Exclude nonphotosynthetically active vegetation in winter images. Data product: Juniper tree cover layer (1‐m resolution)

The model also made use of other open datasets, such as land use, land parcels (although open source in Oklahoma, a dataset was purchased for ease of formatting), roadways and municipal boundaries, which are all publicly available. Each of these datasets has unique characteristics, which were harnessed together to produce the juniper tree species distribution model. A “filtering” methodology was adopted, in which these spatial datasets were processed in a stepwise manner to narrow down to, as the final output, the distribution of juniper species within the CNTT. The final output (i.e., the distribution of juniper tree species) was also verified by collecting a diverse set of land cover ground control points at numerous sites across the study area, during multiple fieldwork campaigns.

Another important feature of this model, and one which helped to guide its development, is the use of Google Earth Engine (GEE) to house model input data and allow processing of large remote‐sensing datasets in an efficient way. It is free for noncommercial use and users must apply for access. Once approved, users can use GEE's full suite of geospatial analysis tools, satellite imagery, and cloud computing resources at no cost (Google, n.d.). It is used for a wide variety of GIS processes, particularly for large‐scale projects or time‐series analyses, including species habitat monitoring (Praticò et al. 2021; Wickramarathna et al. 2021). GEE offers several advantages over other GIS software packages for this type of modeling and workflow, including the ability to process large datasets on a remote server, to rerun updated versions of a model without having to reprocess input datasets, and the ability to export and share large datasets in an automated fashion. While it has some limitations compared to traditional remote sensing software packages, including evaluating spectral information and performing advanced remote sensing workflows, its advantages in processing raster data in an automated way outweighed these limitations for the purposes of this project.

Before adopting a “filtering” approach to identify juniper species, a random forest model was also tested in GEE. While the model performed well in distinguishing between pasture, tree canopy, and general vegetation types, it struggled to consistently differentiate evergreen juniper species from deciduous trees, especially in greener, higher‐rainfall areas. Though the random forest approach may still be useful for small‐scale juniper mapping with high‐resolution imagery or additional spectral data, for the large‐scale application within the CNTT using open‐source data, the adopted filtering approach proved more robust and accurate for the intended purpose. Although other studies have successfully used wavelet analysis (Falkowski et al. 2006, 2017; Poznanovic et al. 2014) and object‐based image analysis (Gustafson et al. 2018; Roth et al. 2021) to accurately classify evergreen species, GEE lacks native support for these techniques, requiring complex external workflows (Google Earth Engine Developers 2023; Pérez‐Cutillas et al. 2023).

### 4.2 Data Sources

The specific datasets used in building the juniper tree species distribution model are listed and described in Table 2. Each dataset was imported into the GEE platform or “called” directly from its location on the GEE server. More information on the use of each dataset within the model is provided in the following sections. The study area of the model was set to the CNTT boundary, obtained from the Chickasaw Nation Geospatial Information Department and also publicly available on the Bureau of Indian Affairs website; this was used to clip all input datasets for processing and analysis.

### 4.3 Land Use Filtering

**FIGURE 4:**
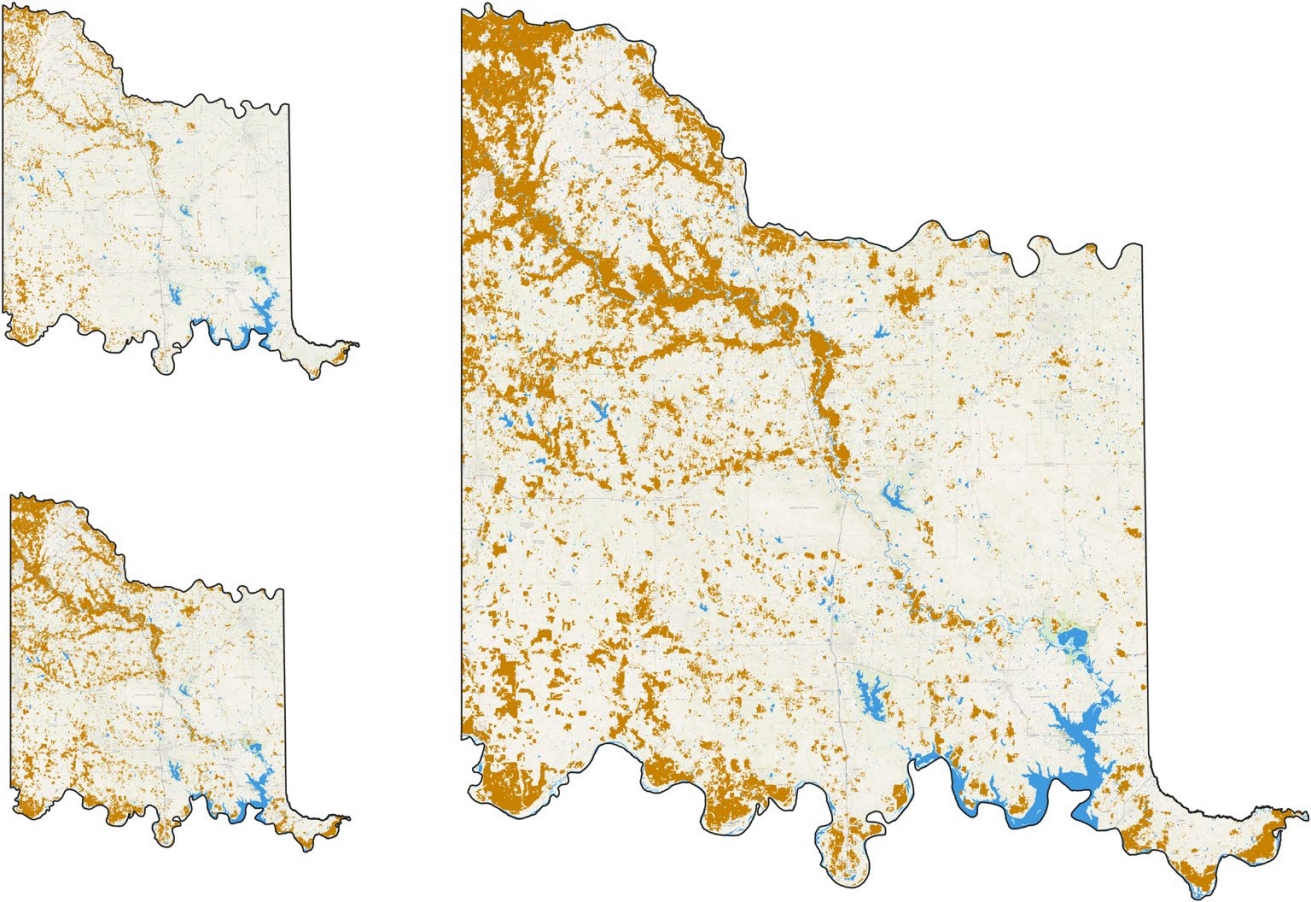
Combined water areas (blue) and crop land (brown) from OKESM (top left) and ESRI (bottom left) and the combined water areas and crop land mask (right). Data Sources: *Oklahoma Ecological System Mapping* (OKESM) https://www.wildlifedepartment.com/lands‐and‐minerals/oklahoma‐ecological‐system‐mapping; Sentinel‐2 Land Cover Explorer. https://livingatlas.arcgis.com/landcoverexplorer/.

After defining the study area, the first step involved removing nonvegetated landcover: water areas, crop land (both vegetated and nonvegetated), roadways and urban areas. To achieve this, a land cover mask was used. The standard 30‐m Landsat Thematic Mapper‐derived land use‐land cover (LULC) data from the National Land Cover Database (Multi‐Resolution Land Characteristics Consortium, n.d.) produced mixed results in defining these areas; therefore, two 10‐m resolution LULC datasets were used to mask water areas and crop lands. These included the Oklahoma Department of Wildlife Conservation's Oklahoma Ecological System Mapping land cover data (Oklahoma Ecological System Mapping, n.d.) and ESRI's Sentinel‐2 land cover data (Sentinel‐2 Land Cover Explorer, n.d.). These datasets were combined to capture the widest possible extent of water areas and crop land. After joining both datasets, the combined mask layer was buffered by 15 m to avoid over‐predicting juniper trees in “edge” areas near water areas and crop lands. Both datasets and the combined land use buffer are shown in Figure 4.

It was discovered that at 10‐m resolution, the LULC raster data for roadways and urban areas tended to exclude vegetated areas near roads and urban areas. Therefore, a more detailed vector road network was used to create a road mask. A road network consisting of several road categories from the Oklahoma Department of Transportation (Oklahoma Department of Transportation 2023) was used so that more vegetated areas (i.e., generally, wooded areas and specifically, juniper trees) were included in the classification. Using a different buffer width for each road category (e.g., local, highway, etc.) solved the problem of over‐masking wooded areas near roadways and urban areas. These buffer criteria, provided in Table 3, were estimated using visual inspection of the various road categories on aerial photography. Each buffered road network was merged into a combined road buffer spatial layer which was used to exclude roadways from subsequent steps in the model.

**TABLE 3:** Buffer distances by road width. Data *Source:* Road types correspond to the ODOT road layers available for download at https://gis‐okdot.opendata.arcgis.com/search?groupIds=ba4c7d77c5f24b1bba7e5027176b9ab6. The “Local Roadways” data layer includes streets and small roads, whereas the “Roadways” data layer includes larger roads, such as county roads and highways.

Road type	Buffer width (ft)
Local roadways	25
Turnpike	250
Ramps/Frontage roads	30
Roadways	Varied by reported road width; if no width was reported then a default value of 30‐ft was used.

**FIGURE 5:**
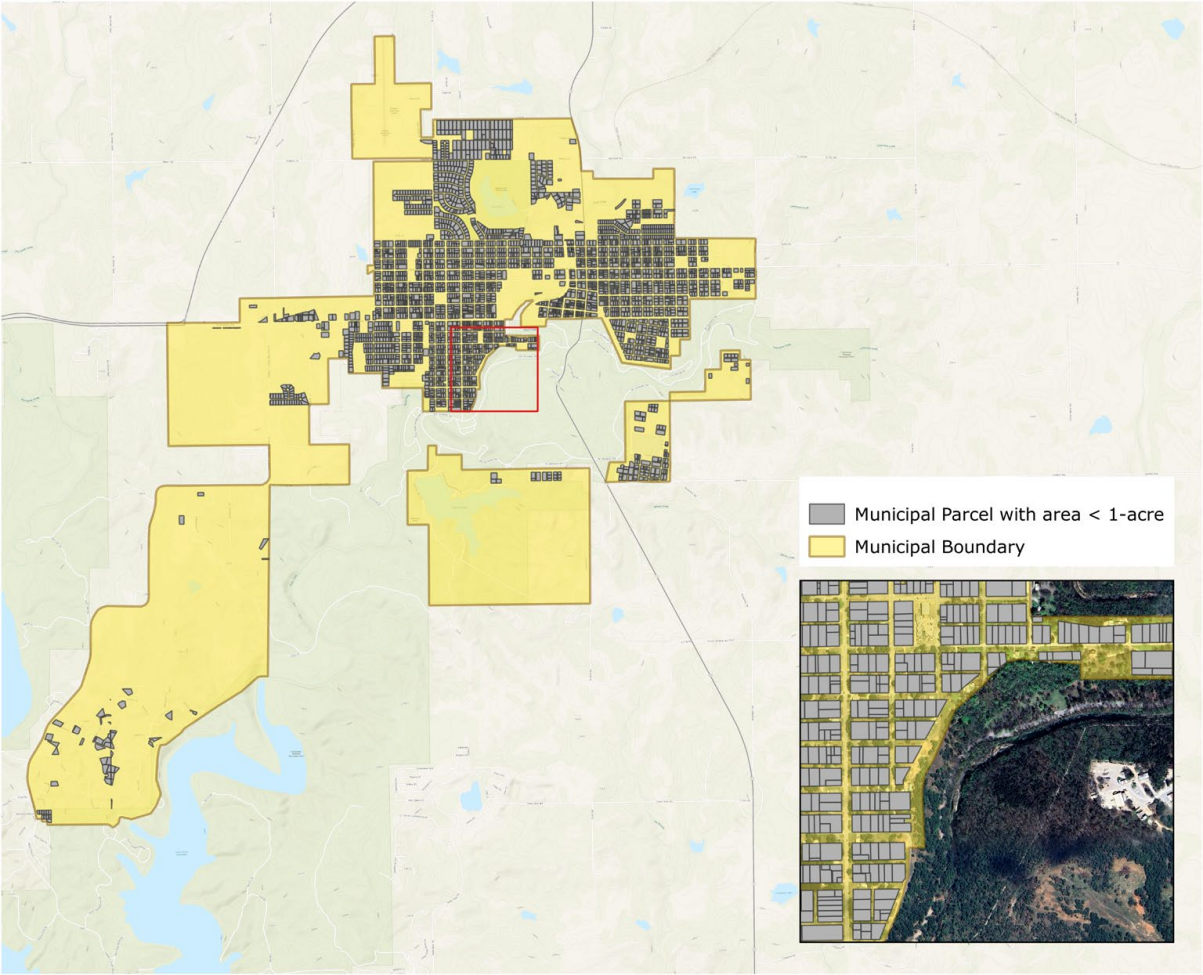
Municipal areas (yellow) and urban parcels (gray, less than one acre excluded from the model).

**FIGURE 6:**
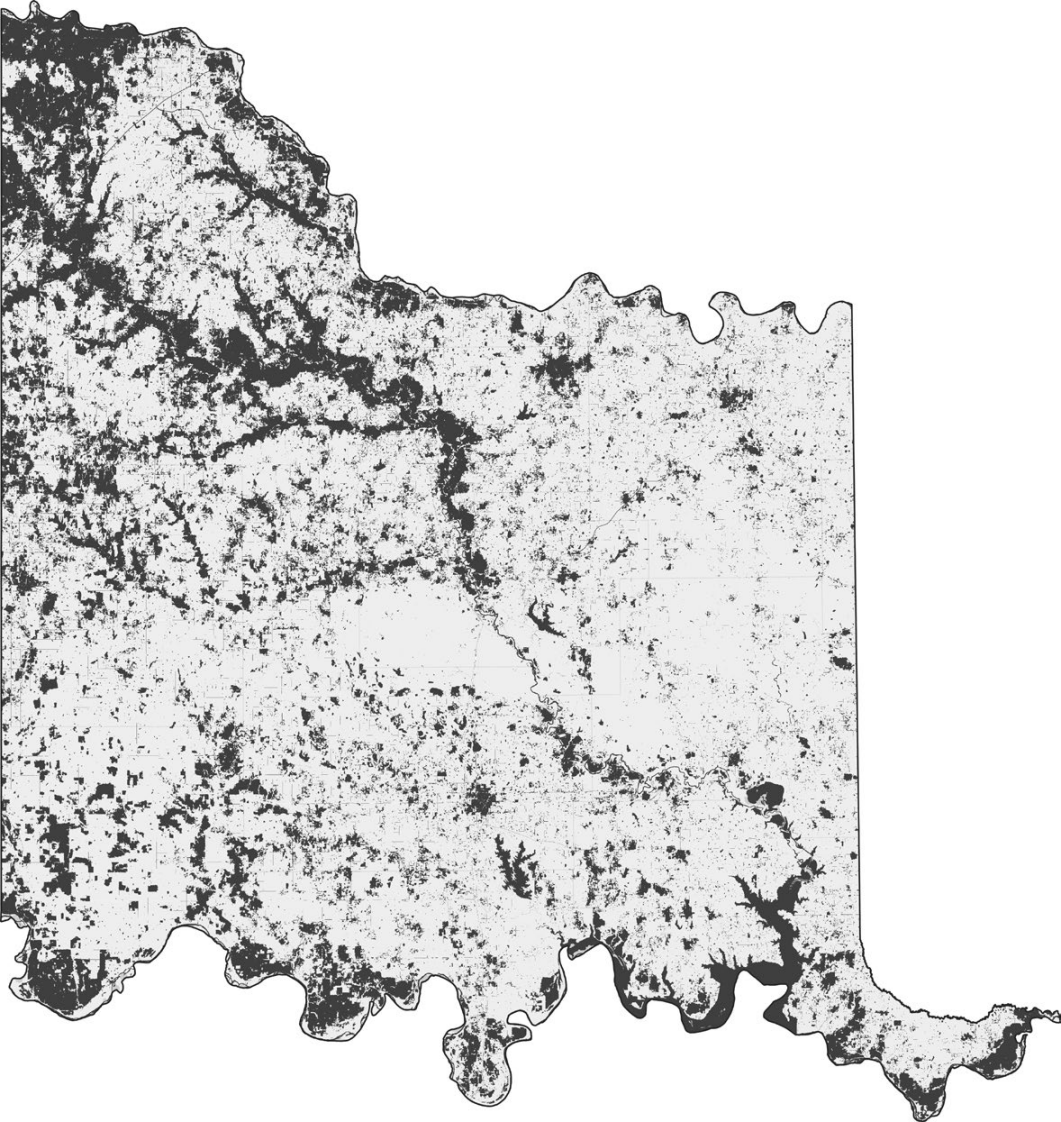
All areas (water areas, crop land, roadways and urban areas) excluded from the model across the Chickasaw Nation Treaty Territory.

To solve the problem of over‐masking wooded vegetation near and within urban areas, municipal boundaries were obtained from the Oklahoma Geographic Information Council's OKMAPS data viewer (OKMaps 2021). In addition, a land parcel geospatial layer was purchased from Visual Lease Services Inc. for the entire study area and all land parcels were intersected with urban areas defined by these municipal boundaries. All parcels less than one acre in size that fell within a municipal boundary were labeled as “urban” and excluded from the model. These urban parcels (an example of which is shown in Figure 5) were also buffered by 30 m to exclude trees near urban areas and in areas where irrigation might take place. Parcel sizes larger than one acre tended to be more mixed in land use and could potentially contain woody vegetation (i.e., juniper species). This buffer distance also allowed for the exclusion of smaller roads, driveways and gaps from previous masked areas. This “urban” layer was combined with the previously described masked roadways to create a final roadways‐urban area exclusion layer. All subsequent model steps were carried out only in areas outside of these exclusion areas (i.e., outside of water areas, crop land areas, roadways, and urban areas). All excluded areas, including urban areas, are shown in Figure 6.

### 4.4 Canopy Height Filtering

**FIGURE 7:**
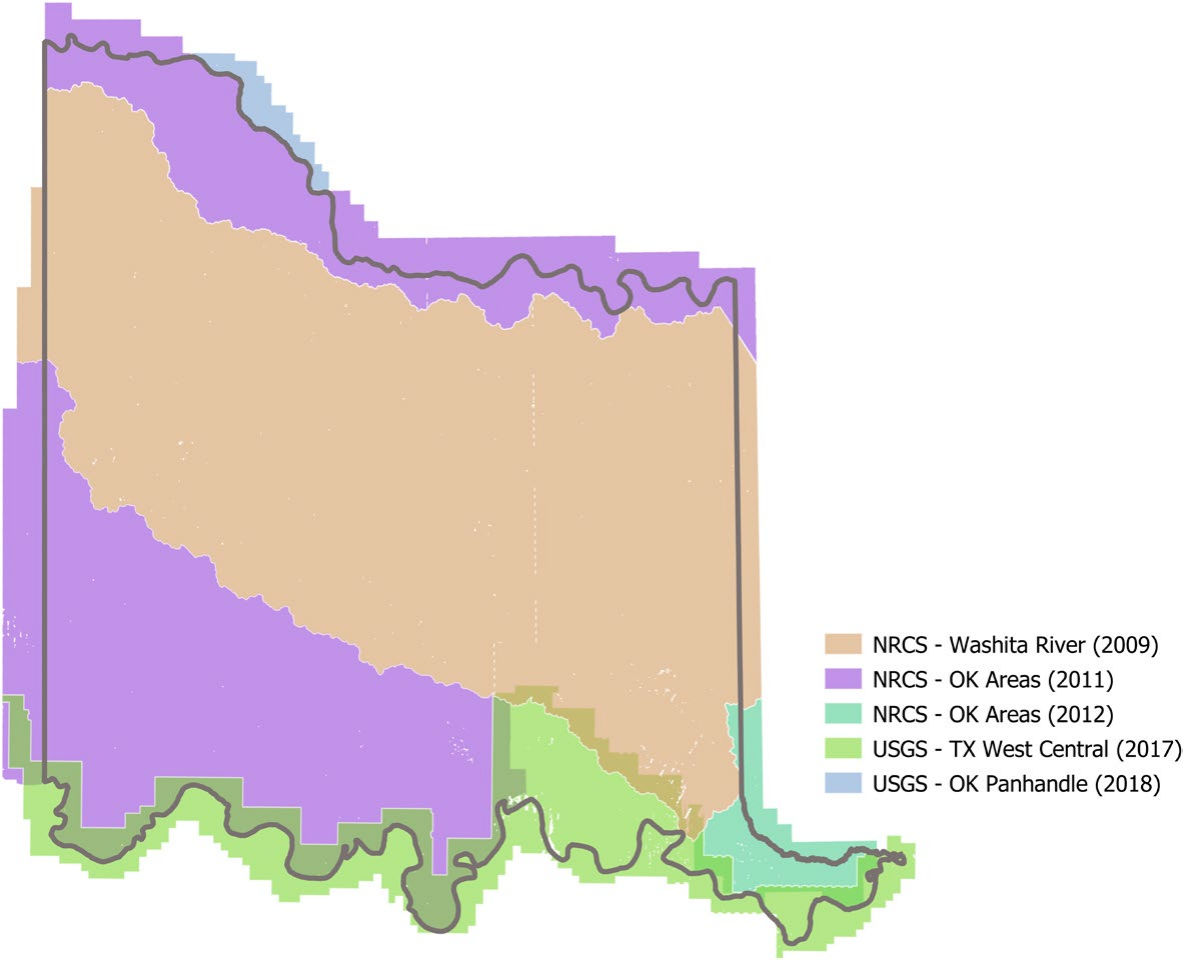
LiDAR datasets used in the Juniper Tree Species Distribution Model.

**FIGURE 8:**
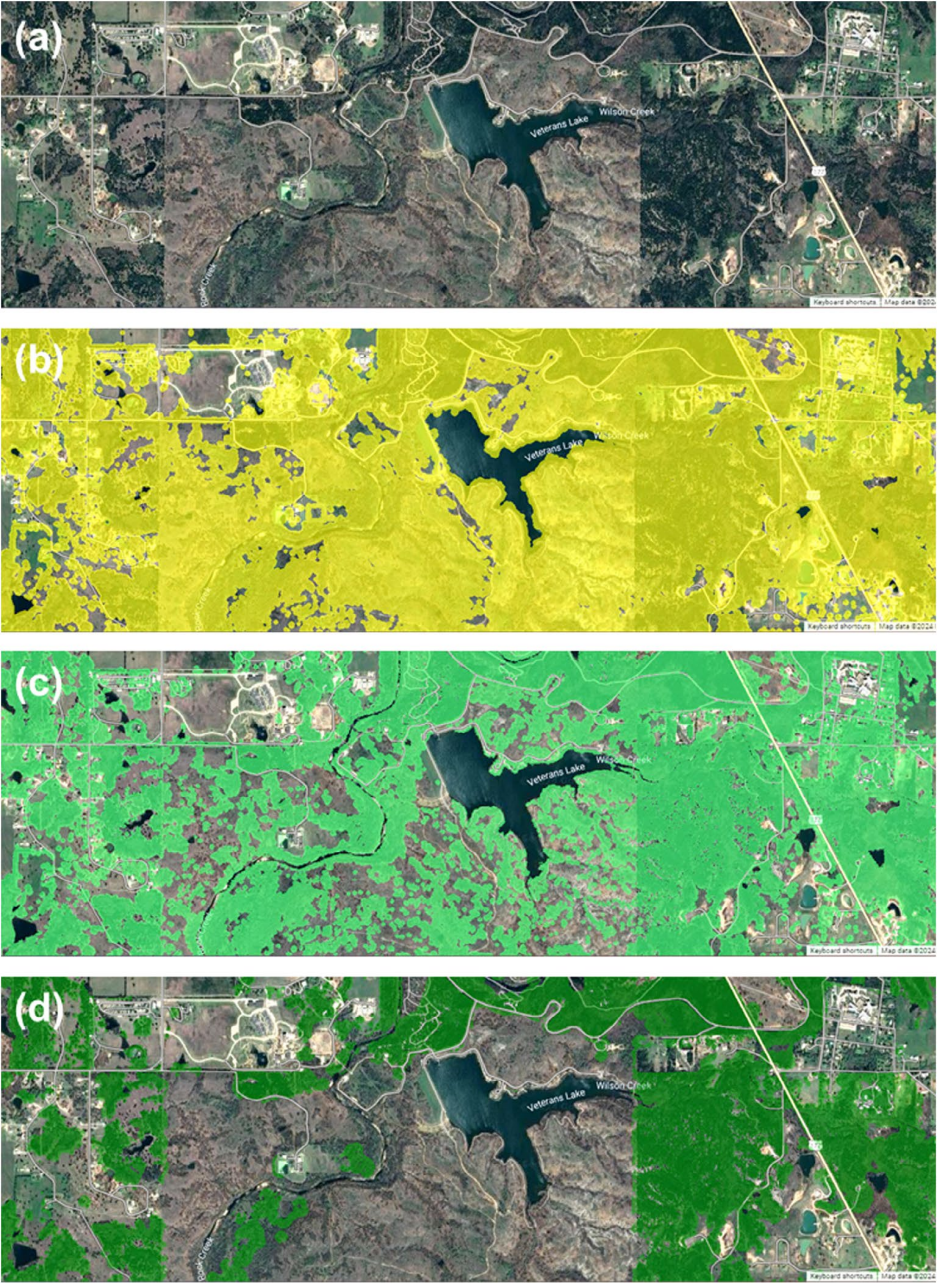
Google Earth Engine workflow shown over a sample site in Murray County, OK. (a) No model output; (b) Canopy height layer (canopy cover); (c) NDVI‐ and EVI‐filtered NAIP layer (tree cover); (d) NDVI‐filtered Sentinel‐2 layer (juniper cover).

At this stage of the methodology, wooded, pasture, and bare ground land cover areas remained after the land use filtering was performed. LiDAR datasets (listed in Table 4 and shown in Figure 7) from the Oklahoma Geographic Information Council's OKMAPS data viewer (OKMaps 2021) and USGS's Lidar Explorer (Lidar Explorer, n.d.) were incorporated to mask out flat areas and therefore preserve areas with a tree canopy (i.e., wooded areas). All 3878 LiDAR point cloud tiles covering the CNTT were obtained and processed into one‐meter canopy height grids using Global Mapper software (Blue Marble Geographics, n.d.). These grids were merged in GEE, taking the maximum height in areas of overlap, to create a final canopy height layer, an example of which is shown in Figure 8b. This layer was filtered to only include areas with a canopy height of one foot or greater, thereby excluding flat, nonforested areas. This layer was then processed by smoothing, eroding, and dilating processes in GEE to remove small “islands” within the data and smooth the resulting canopy height layer. All remaining areas outside of this layer were considered “flat” and were excluded in the further classification steps. (Note that while Global Mapper was used for convenience for this step, other open‐source software applications could also be used).

**TABLE 4:** LiDAR datasets used to build the canopy height layer.

LiDAR dataset	Collecting agency	Date(s) collected
TX West Central	USGS	2017
OK Areas	NRCS	2011–2012
OK Panhandle	USGS	2018
Washita river	NRCS	2009

Since Oklahoma is covered by LiDAR datasets collected in various years, a certain level of error was introduced in this step into the final juniper classification. However, using this data was more beneficial than not using it, as its inclusion improved the accuracy of the final juniper tree species distribution output.

### 4.5 Tree Canopy Filtering

**FIGURE 9:**
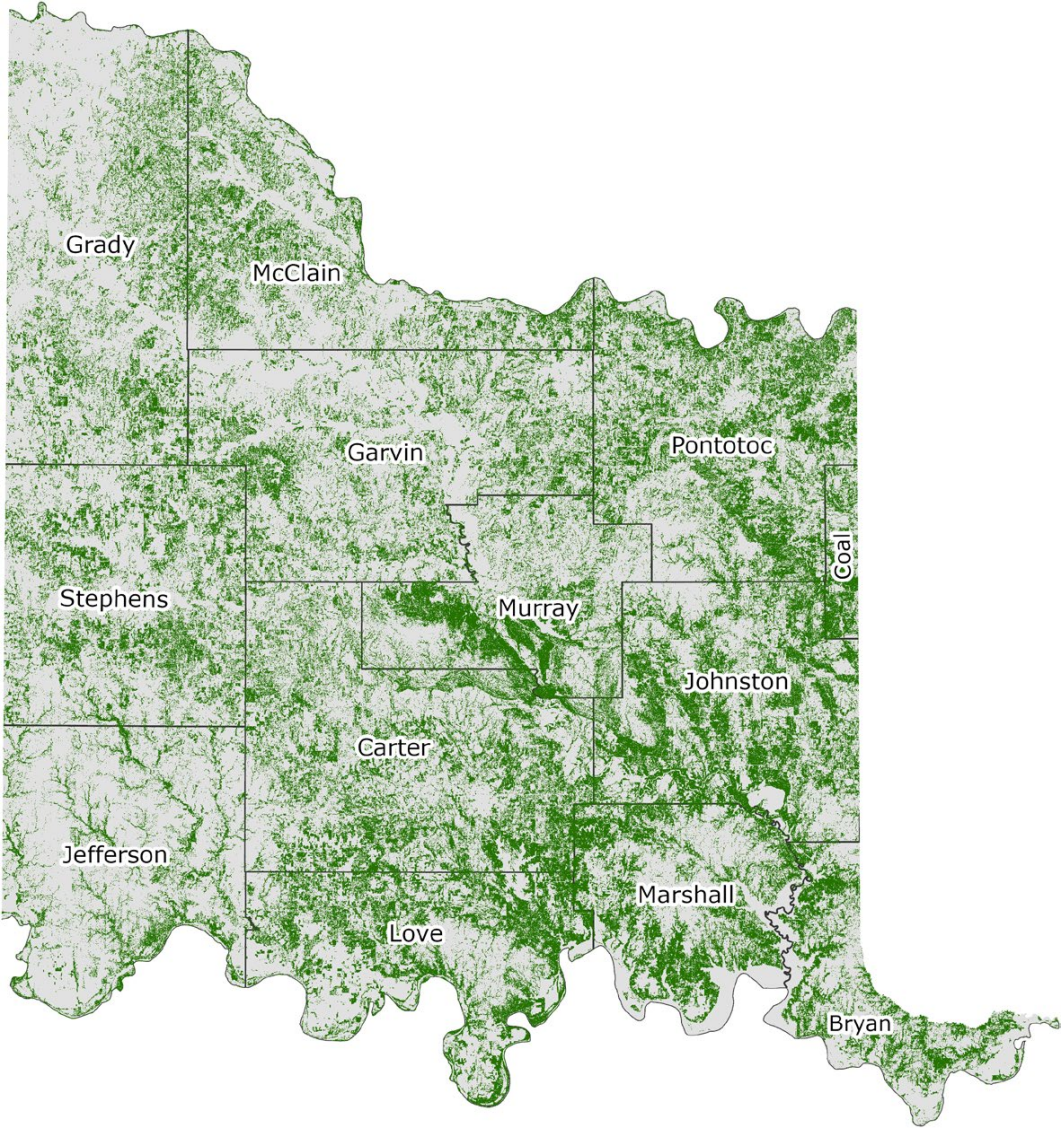
Model output of tree cover across the Chickasaw Nation Treaty Territory.

The canopy height layer described previously did not necessarily contain only tree canopy, as any areas with a height above 1 ft were included. Therefore, from the smoothed canopy height layer, a “tree” or “wooded area” tree canopy layer was created, using the four‐band aerial photography (B, G, R, NIR) from USDA's NAIP leaf‐on data, obtained from the 2021 NAIP dataset available in GEE. The normalized difference vegetation index (NDVI) was calculated using the 2021 NAIP data across the study area, and this was filtered with a threshold of 0.1 to identify all green areas (i.e., photosynthetically active vegetation) that coincided with the smoothed canopy height layer described in the previous section. In addition, an Enhanced Vegetation Index (EVI) filter with a threshold of 1.8 was applied to further distinguish between tree canopy, grasses, and other nontree vegetation that were not excluded from the study area in earlier stages of the methodology. In both the case of the NDVI index and the EVI index, the inclusion thresholds were determined by trial and error using aerial photography and field data. Although NDVI is widely used for assessing vegetation greenness, it can saturate in areas with dense canopy cover, whereas EVI can enhance sensitivity in high‐biomass regions (Jensen 2016; Gao et al. 2000; Boegh et al. 2002; Huete et al. 2002). It was determined that combining the use of NDVI with EVI produced a better representation of total tree cover. A portion of the NDVI‐ and EVI‐filtered layer is shown in Figure 8c. The model output for tree cover for the entire study area is shown in Figure 9.

### 4.6 Juniper Filtering

Lastly, the European Space Agency's Sentinel‐2 10‐m leaf‐off satellite imagery (2020–2022) was used in GEE according to the following steps. Sentinel‐2 images were filtered to keep those within the study area that contained less than 10% cloud cover and were collected in winter (between January 1 and February 14) of the years 2020 through 2022 (i.e., 3 years in total). Only winter images were analyzed due to the “leaf‐off” condition of deciduous trees during winter, allowing evergreen juniper species to be distinguished from other vegetation. The set of filtered images was captured over 21 separate dates across this time period. The 93 images in this set were then merged into a final Sentinel‐2 layer by calculating mean pixel values across all images. This composite image was subsequently used to produce a Sentinel‐2 based NDVI layer to identify juniper species (i.e., distinct from the NAIP‐derived NDVI layer used to produce the tree cover layer described previously). This layer provided an indication of the average intensity of green vegetation for each raster pixel over the time period of imagery collection.

**FIGURE 10:**
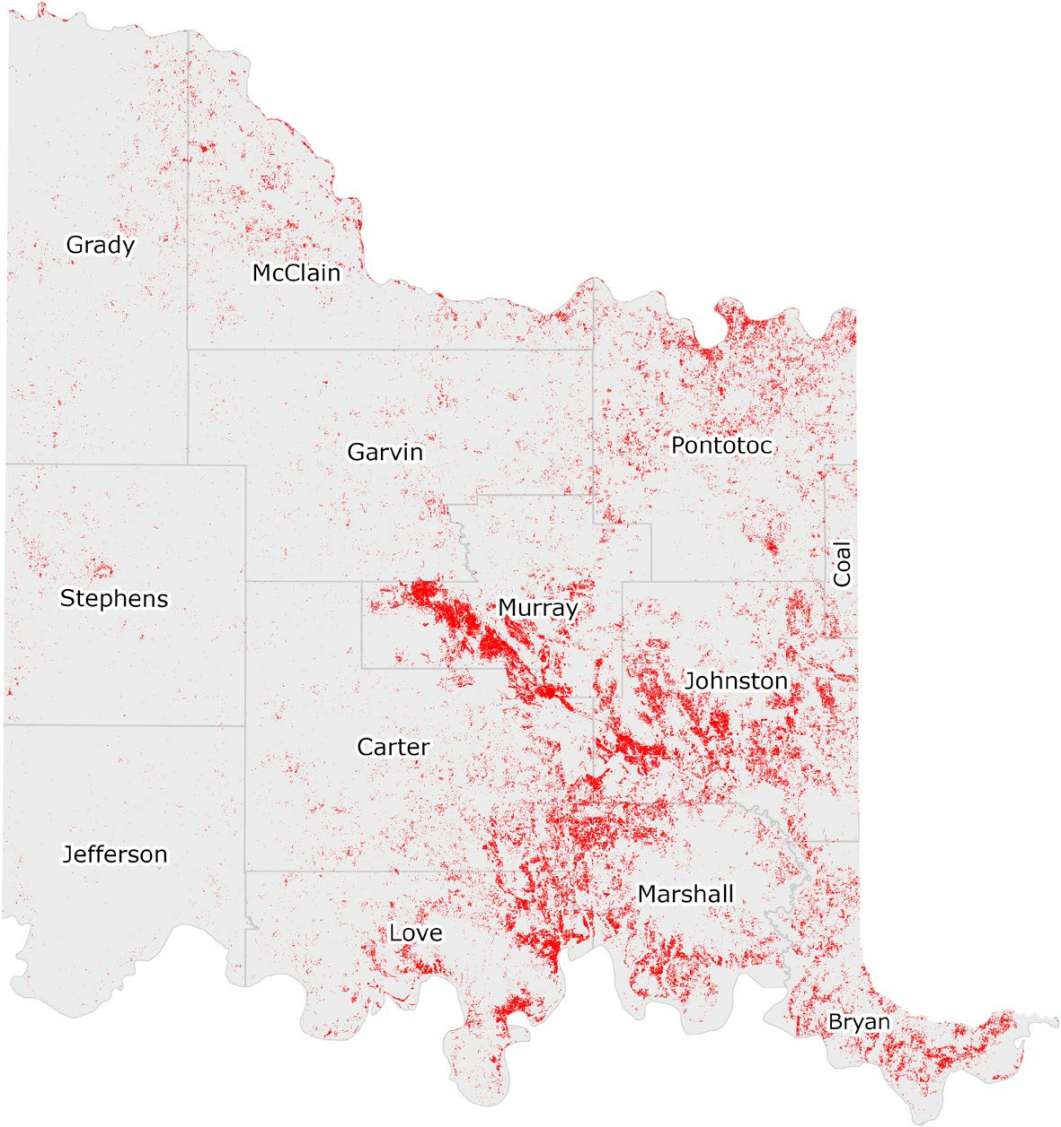
Modeled juniper tree species distribution within the CNTT. Major juniper species concentrations are found within the Arbuckle Mountains, Arbuckle Uplift, and Eastern Cross Timbers ecoregions.

The wooded areas identified from the tree canopy filtering steps described previously were further filtered to identify evergreen juniper species as pixels with an NDVI value greater than 0.35. Several values between 0.3 and 0.4 for the NDVI threshold were applied during model development, but 0.35 provided the best results when compared against training data collected within the CNTT. The resulting juniper tree species distribution layer was then further refined using another erosion/dilation function in GEE to smooth the output and remove small “islands” and gaps. A portion of this data layer is shown in Figure 8d and the entire juniper tree species distribution layer is shown in Figure 10.

### 4.7 Training Data Collection

**FIGURE 11:**
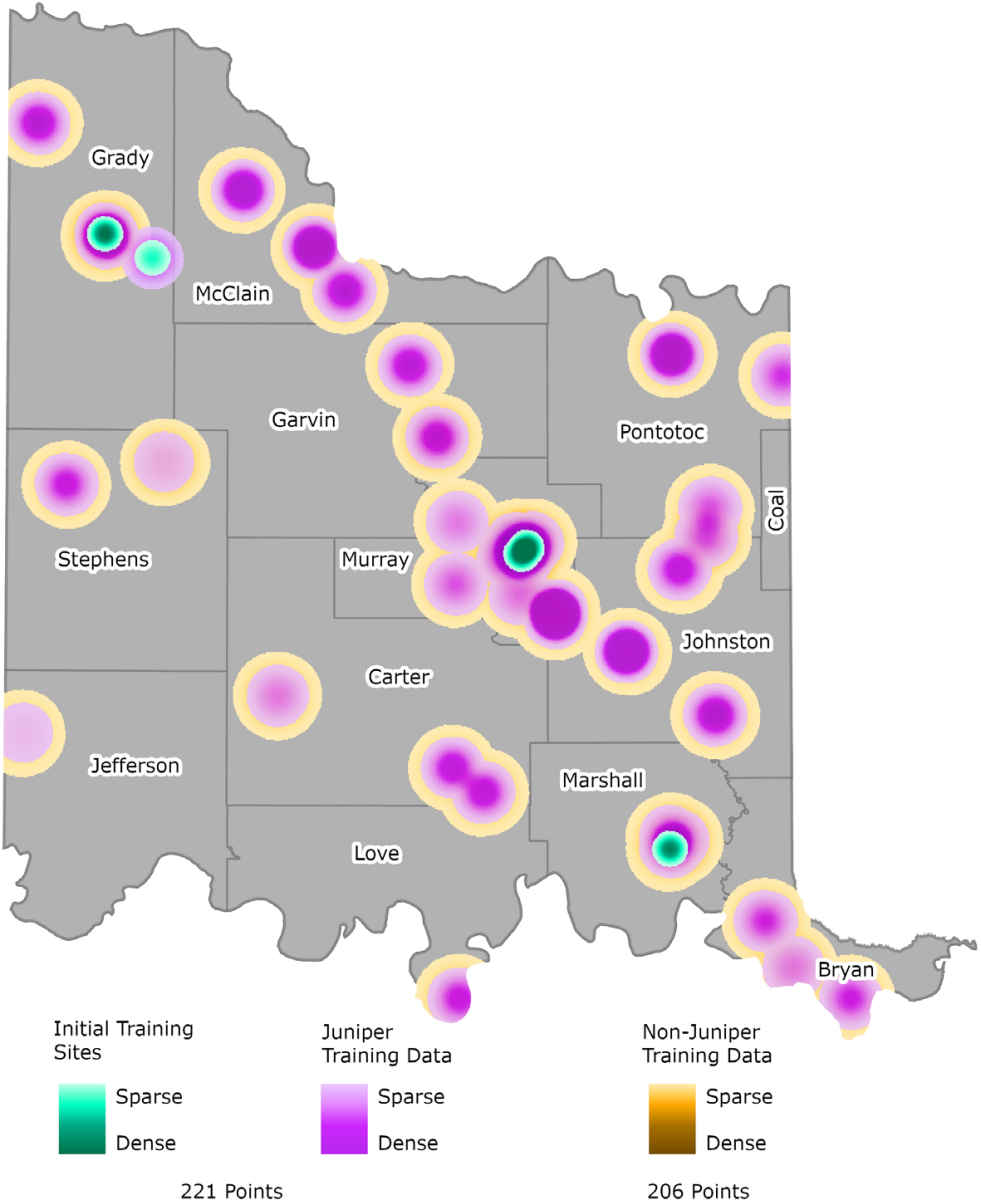
Training Data for Model Development (March 2023).

**FIGURE 12:**
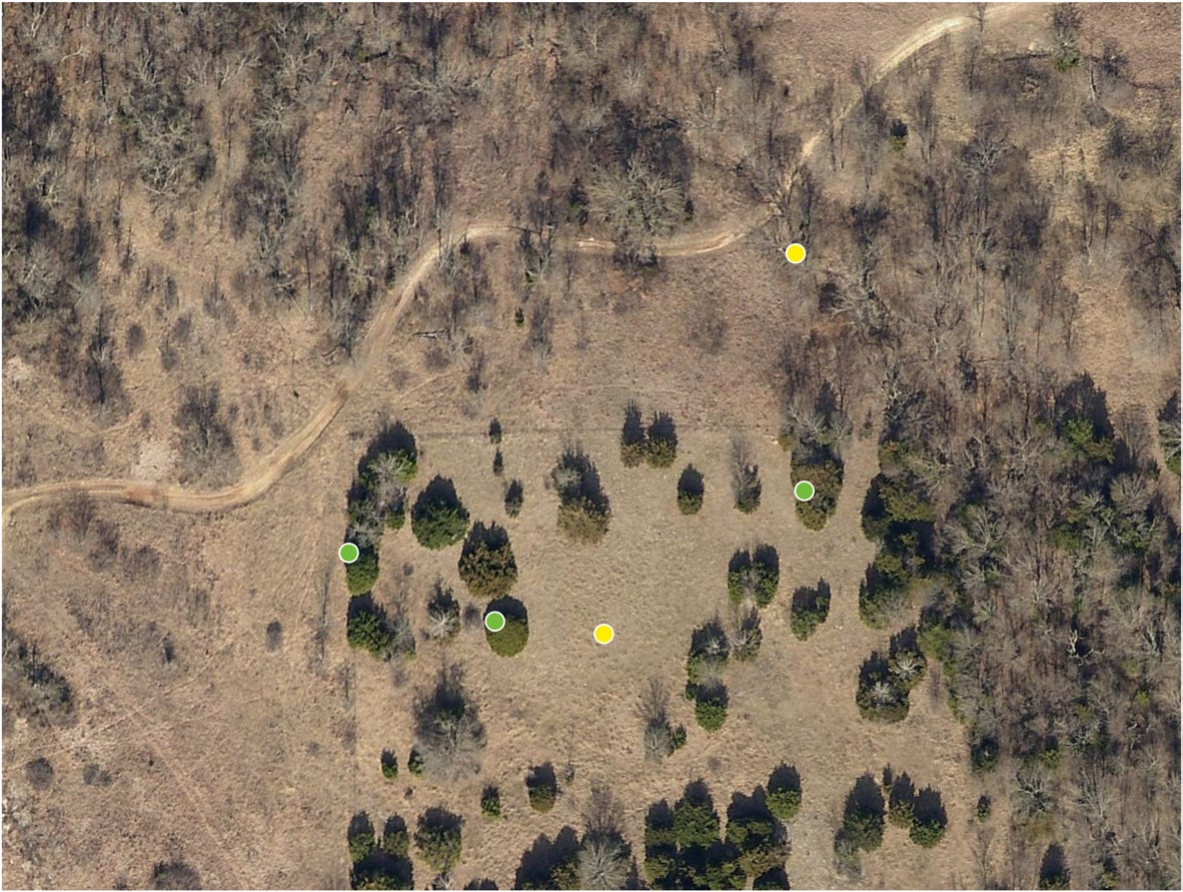
Example of field points collected in November 2023. Green circles are “juniper” points, and yellow circles are two “nonjuniper” points, bare ground and deciduous tree.

A field campaign to collect training data for the beginning phases of the juniper tree species distribution model development was performed in March 2023 (Figure 11). A northwest–southeast transect across the CNTT was established (green areas in Figure 11) representing various environmental conditions in which juniper trees are found. During the initial field campaign, high accuracy “juniper” and “nonjuniper” control points (i.e., tree species other than junipers and other areas—mainly unimproved roadways, pasture, and crop land) were captured using a Trimble TDC150 with centimeter accuracy. These control points were further supplemented with training data derived from high‐resolution imagery data and drone photography (purple and brown areas in Figure 11). An example of a field site with ground control points is shown in Figure 12. Altogether, 427 control points were collected in the field or by using imagery (221 juniper points and 206 nonjuniper points).

### 4.8 Accuracy Assessment Data Collection

**FIGURE 13:**
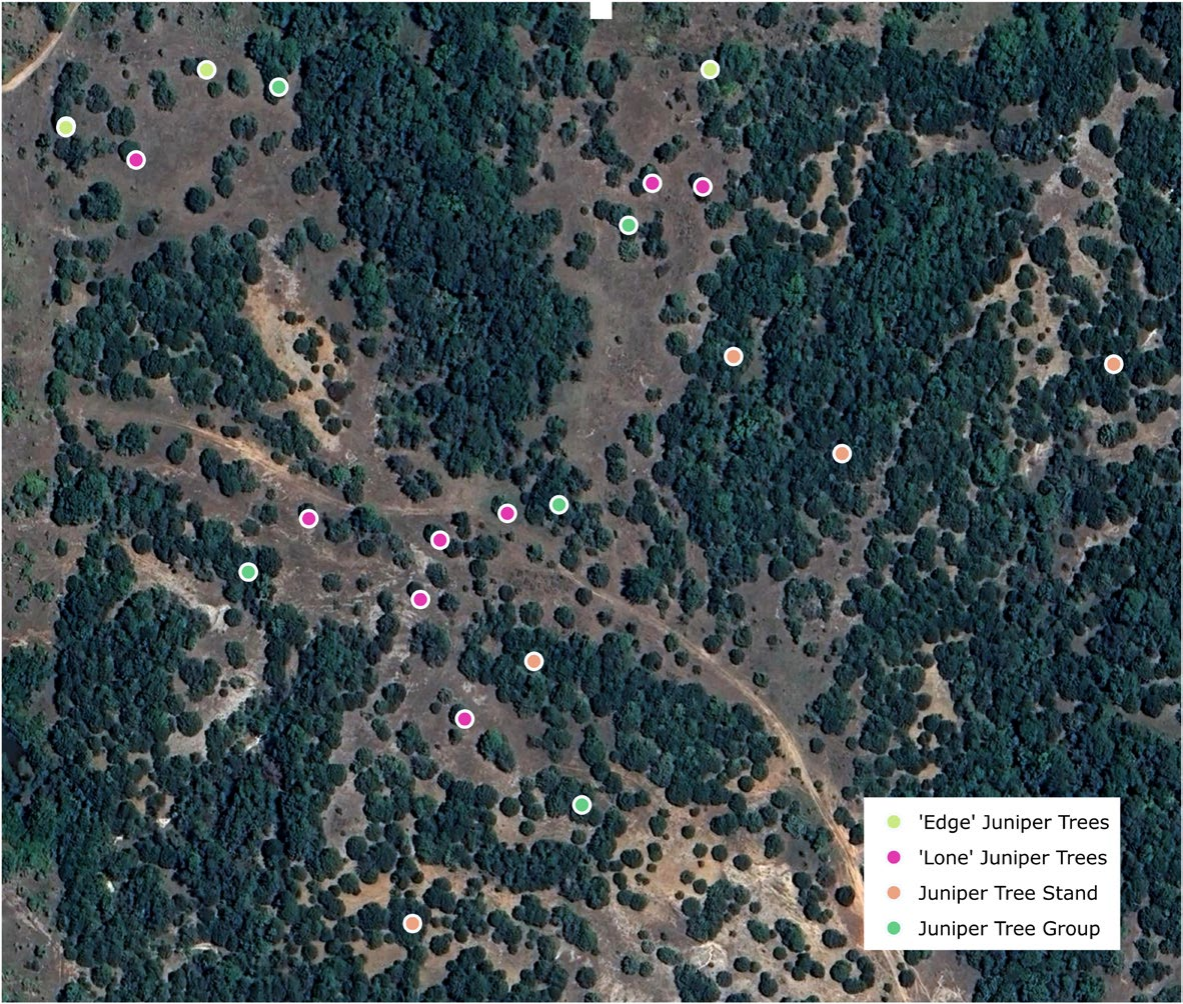
An example of juniper tree field validation points, segmented into four classes.

**FIGURE 14:**
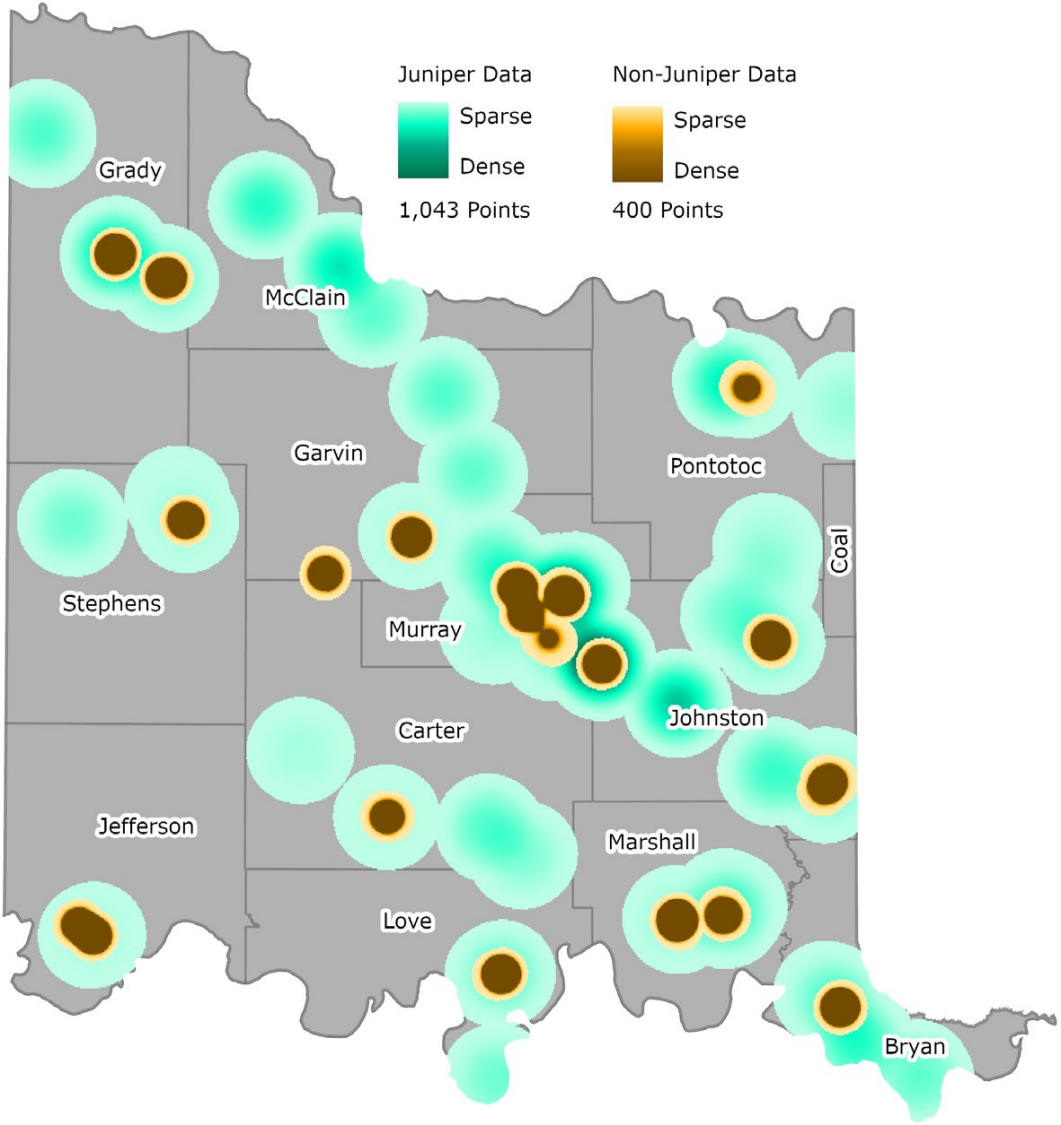
Model Validation and Accuracy Assessment data points.

To ensure the juniper tree species distribution model accurately represented juniper trees present on the ground, a model validation field campaign was undertaken between November 2023 and April 2024. In total, 55 “juniper” points and 139 “nonjuniper” points were collected, and these points were supplemented with points derived from high‐resolution imagery, to create a validation dataset of 1443 points (including 1043 “juniper” points and 400 “nonjuniper” points). The juniper tree field validation points were segmented into four classes, as described in Table 5. An example of this segmentation of field points is shown in Figure 13, and the full dataset is represented in Figure 14. To assess the accuracy of the final model output, an error matrix with producer's accuracies, user's accuracies, overall accuracies, and kappa coefficient values was calculated. Although ground samples for validation were taken 2 years after the aerial photography and satellite imagery were captured, and there have been alterations in land cover, any sites with apparent changes were avoided in the process of using training and accuracy assessment data points.

**TABLE 5:** Segmentation of Juniper Validation Points into four classes.

Juniper tree group type	Description	Number of points in the validation dataset
“Lone” Juniper Tree	A single juniper tree	309
“Edge” Juniper Tree	A juniper tree located along a fence line or roadway, found with other edge trees in a linear orientation	248
Juniper Tree Group	A small group (i.e., less than six) of juniper trees	244
Juniper Tree Stand	A large group (i.e., more than six) of juniper trees	242

### 4.9 Juniper Vulnerability Stages

**FIGURE 15:**
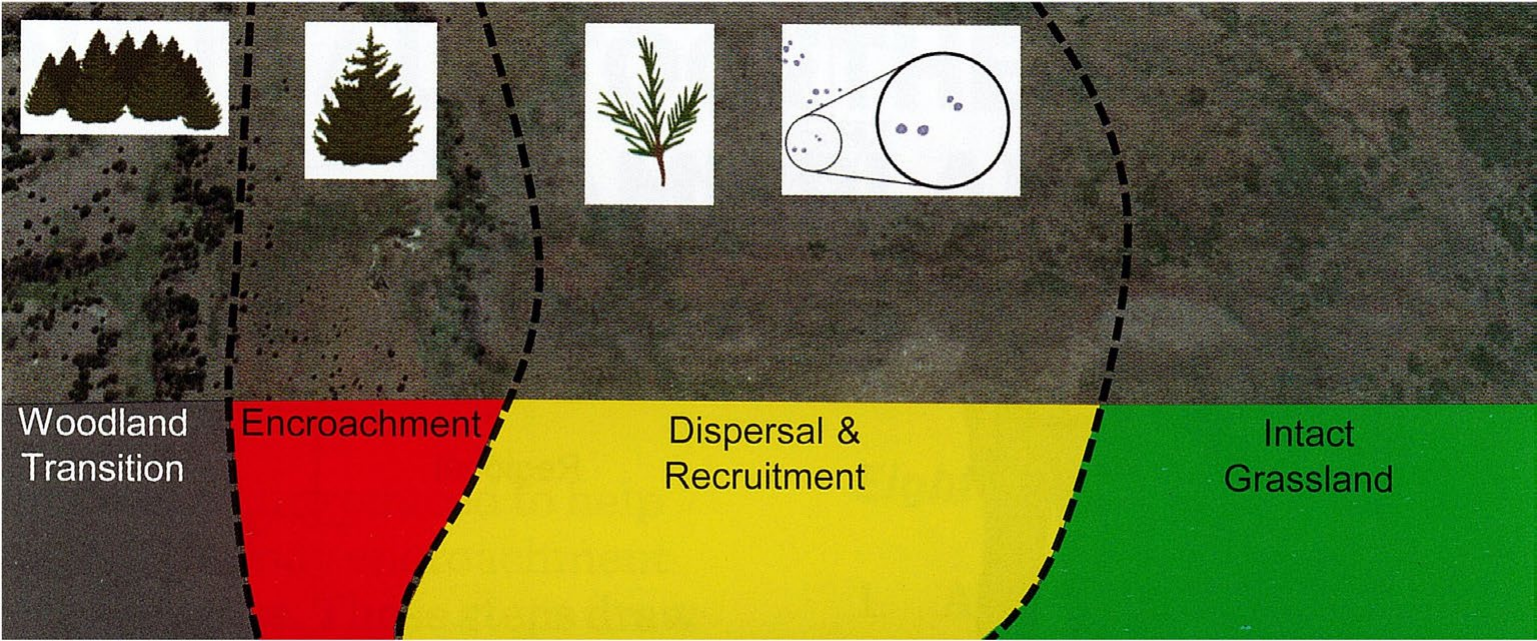
Woody Plant Encroachment Vulnerability Spectrum (Twidwell et al. 2021).

To model vulnerability throughout the CNTT, the Woody Encroachment Vulnerability Guide (Twidwell et al. 2021) was used as the overarching roadmap. At the core of this guide is the definition of five stages of woody plant encroachment, which include Intact Grassland (called Intact Areas for this project), Dispersal, Recruitment, Encroachment, and Woodland Transition.^1^ For the purposes of this project, and in keeping with the source methodology, the dispersal and recruitment stages were combined into one class. These stages are described in more detail in Table 6 and are shown graphically in Figure 15.

**TABLE 6:** Description of Woody Plant Encroachment Vulnerability as implemented in the juniper tree species distribution model (adapted from Twidwell et al. 2021).

Vulnerability stage	Category summary/Description	Juniper distribution model approach
Woodland transition	The final stage of woody encroachment refers to the collapse of the grassland ecosystem and a transition to woody plant dominance.	This stage is equivalent to the modeled juniper distribution. These are areas of dense juniper tree cover.
Encroachment stage	The encroachment stage is identified by the presence of seed‐bearing trees.	The encroachment stage is modeled as a 50‐m buffer around the Woodland Transition stage.
Dispersal stage	Grasslands in the dispersal stage are contaminated by seed.	These categories were combined in the model to mimic the grouping used by the NRCS. This stage is modeled as a 200‐m buffer around the modeled Encroachment stage.
Recruitment stage	Recruitment is a stage of spatial expansion—where self‐sustaining woody populations expand from parental sources to eventually grow to maturity and become potential future seed sources.	
Intact stage	Intact areas have minimal vulnerability to woody plant encroachment because they have no exposure to the source of the encroachment problem (seed).	This stage is represented by all areas that are not included in the categories above (also excluding water areas, croplands, roadways, and urban areas).

To identify zones of juniper species encroachment across the CNTT, the output from the juniper tree species distribution model described previously was used to develop four raster‐based woody encroachment vulnerability layers using GEE. The encroachment stage was defined by applying a 50‐m buffer around the modeled woodland transition layer, while the dispersal and recruitment stage was delineated by an additional 200‐m buffer extending from the edge of the encroachment layer. The design of the buffers used in these two main vulnerability layers (i.e., encroachment and dispersal and recruitment) was guided by the Woody Encroachment Vulnerability Guide (Twidwell et al. 2021), consultation with the Oklahoma NRCS, and field data from Major Land Resource Areas (MLRAs) across the CNTT (United States Department of Agriculture, Natural Resources Conservation Service 2022). Areas outside of these buffer areas were classified as intact areas, indicating no modeled presence of juniper, although other woody species may be present. The decision to use meters over yards was consistent with current units used for remotely sensed data and provided a conservative buffer. This classification framework was then used to spatially assess the vulnerability of land parcels in CN watersheds to juniper expansion. Esri's Experience Builder (Esri 2025) was used to build an interactive tool as the public outward‐facing platform for all the data.

## 5 Results

### 5.1 Accuracy Assessment

A segmented error matrix (Table 7) comparing the final juniper distribution (Figure 10) and the juniper tree field validation points (Figure 14) was generated. Table 7 demonstrates that the model did not classify “lone” and “edge” juniper trees as successfully as “group” and “stand” juniper trees. Only 34 of the 309 lone juniper trees were correctly classified as compared to the success of 219 of the 242 “stand” juniper trees being correctly classified. From the error matrix, producer's accuracies, user's accuracies, overall accuracies (Story and Congalton 1986; Jensen 2016), and kappa coefficient values (Congalton 1991) were calculated for each validation point class and are provided in Table 8. This shows that the producer's accuracy, the probability of a juniper tree reference pixel supplied from the field being correctly classified is low for lone (11.00%) and edge (14.11%) trees and high for group (79.51%) and stand (90.50%) trees. The user's accuracy, the probability that a pixel classified by the model represents that validation point class (e.g., lone, edge, group, or stand) on the ground is relatively high for all classes, lone (91.89%), edge (92.11%), group (98.48%) and stand (98.65%).

**TABLE 7:** Error matrix, segmented by Juniper Tree Field Validation Point Class.

		Validation data					
		“Other” land cover	Juniper trees				Total
			Lone	Edge	Group	Stand	
Classified data	“Other” land cover	397	275	213	50	23	958
	juniper trees	3	34	35	194	219	485
	Total	400	309	248	244	242	1443

**TABLE 8:** Producer's and User's Accuracy, Overall Accuracy and Kappa Coefficient, segmented by Juniper Tree Field Validation Point Class.

Juniper validation point class	Producer's accuracy		User's accuracy		Overall accuracy	Kappa coefficient
	“Other” land cover	Juniper trees	“Other” land cover	Juniper trees		
Lone	99.25%	11.00%	59.08%	91.89%	60.79%	0.1139
Edge	99.25%	14.11%	65.08%	92.11%	66.67%	0.1593
Group	99.25%	79.51%	88.81%	98.48%	91.77%	0.8183
Stand	99.25%	90.50%	94.52%	98.65%	95.95%	0.9124
Combined	99.25%	46.21%	41.44%	99.38%	60.91%	0.3179

Overall accuracies for each juniper validation point class (Table 8) show that there are lower class accuracies for lone (60.79%) and edge (66.67%) trees and higher class accuracies for group (91.77%) and stand (95.95%) trees. Combined juniper validation point set overall accuracies (Table 9) increase from 60.91% to 95.95% when excluding lone and edge juniper trees. The kappa coefficient for each juniper validation point class (Table 8) shows a similar trend when compared to the overall accuracies whereby the lone (0.1139), edge (0.1593), and combined classes (0.3179) show a fair to slight agreement and the group (0.8183) and stand (0.9124) classes demonstrate a substantial to almost perfect agreement (Landis and Koch 1977).

**TABLE 9:** Overall Accuracy, segmented by Juniper Tree Field Validation Point Class.

Juniper validation point set	Overall accuracy
Combination of “Edge,” “Lone,” “Group’,” and “Stand” Points	60.90%
Combination of “Edge,” “Group,” and “Stand” Points	74.51%
Combination of “Group” and “Stand” Points	91.42%
“Stand” Points Only	95.95%

### 5.2 The JET and Vulnerability Stages

**FIGURE 16:**
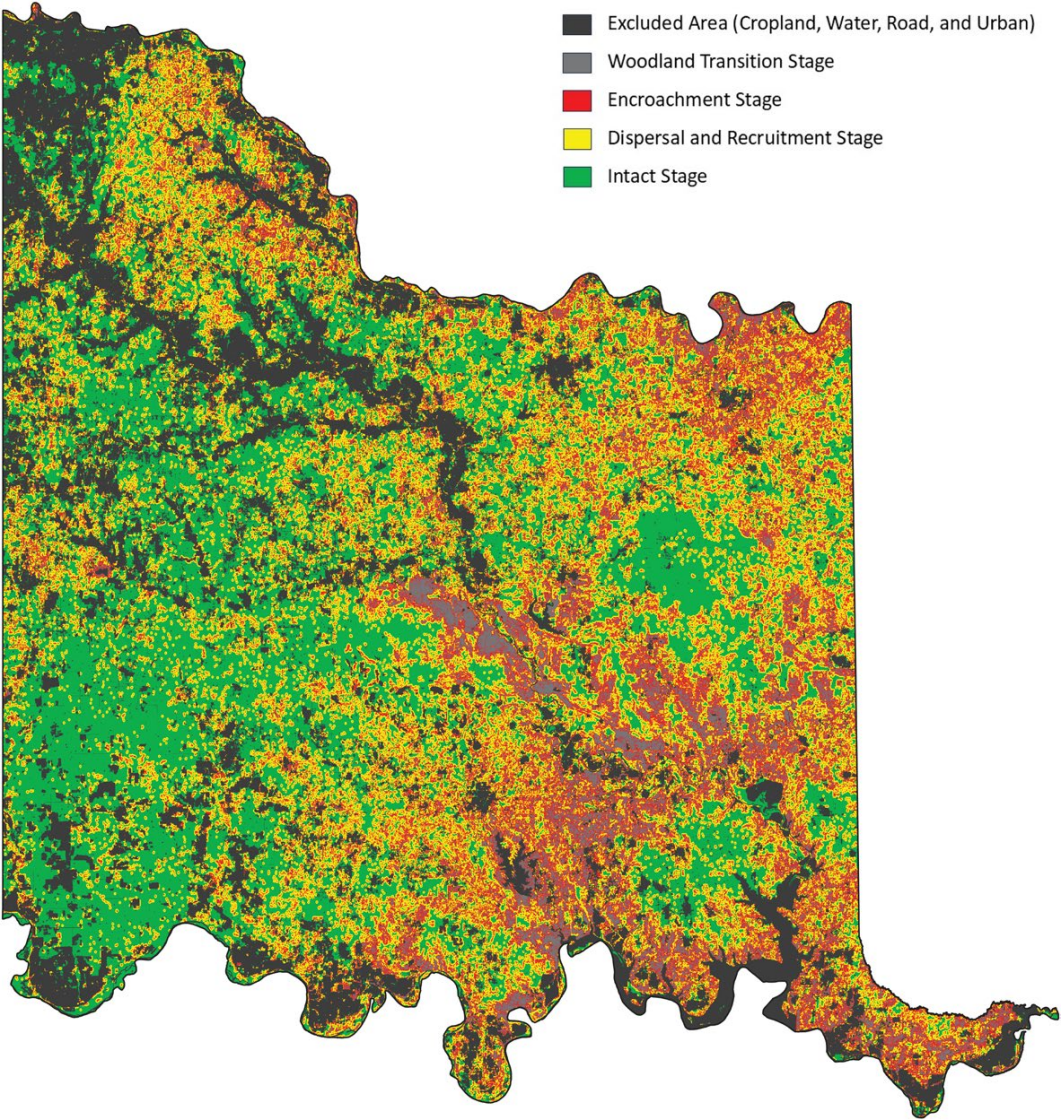
Model output of vulnerability across Chickasaw Nation Treaty Territory.

The JET was developed by the Center for Spatial Analysis at the University of Oklahoma in Norman, Oklahoma (https://www.ou.edu/ags/csa) and is housed at the Oka Institute at East Central University in Ada, Oklahoma (https://www.okainstitute.org/) (The Oka Institute, n.d.). It was created as a decision‐support tool for stakeholders to be able to view and interact with the vulnerability data described in the previous sections. A total of six raster data sets were produced for the CNTT in GEE: tree cover, four vulnerability stages of juniper encroachment (juniper cover or woodland transition, encroachment, dispersal and recruitment, and intact areas) and exclusion (i.e., all areas excluded from the stepwise model) (Figure 16). Each of these raster layers was exported from GEE and a zonal statistics function was used in QGIS (QGIS 2024) to summarize the area of each vulnerability stage in each of the four mapping units available in the JET: (i) parcel, (ii) watershed, (iii) county, and (iv) CNTT. Other attributes were also summarized in GEE and QGIS to create the following attributes for each mapping unit dataset displayed in the JET: parcel ID, parcel area (acres), woodland transition area (acres), woodland transition (%), encroachment area (acres), encroachment (%), dispersal and recruitment area (acres), dispersal and recruitment (%), intact areas (acres), intact areas (%), tree cover area (acres), tree cover (%), percent of juniper cover within tree cover, average elevation (ft), average slope (%), average juniper height (ft), median juniper height (ft), crops and water areas (acres), crops and water (%), urban and roads area (acres), urban and roads (%), exclusion (acres), and exclusion (%). The definitions of these attributes can be found on the JET homepage (The Oka Institute, n.d.).

**FIGURE 17:**
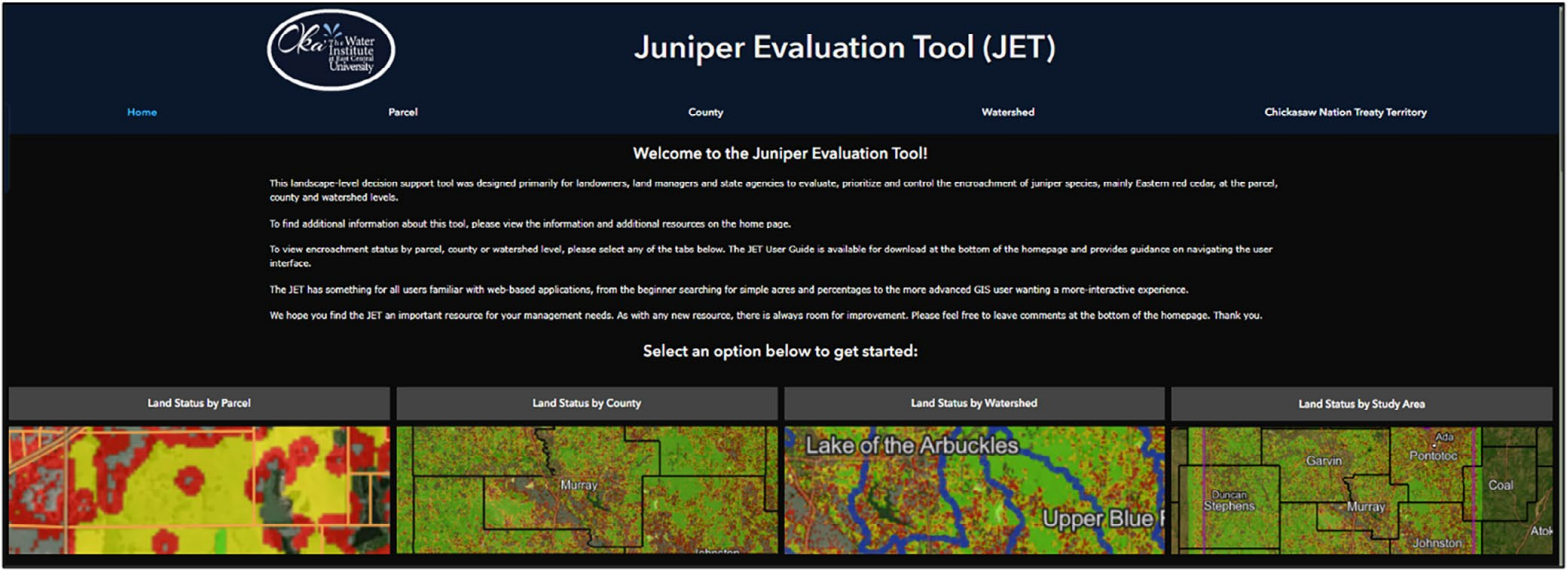
The upper portion of the JET homepage showing the site navigation ribbon, welcome message, and visual options to select the scale. Juniper Evaluation Tool (JET)|The Oka Institute Spatial Data Center.

The JET contains a homepage (Figure 17) that is broken into nine sections: site navigation ribbon, welcome message, visual options to select the scale, project background and scope, color class definitions, content blocks (attribute definitions, model explanation, economics of prescribed fire and fact sheets), frequently asked questions, user guide, and project partners with a feedback submission link.

**FIGURE 18:**
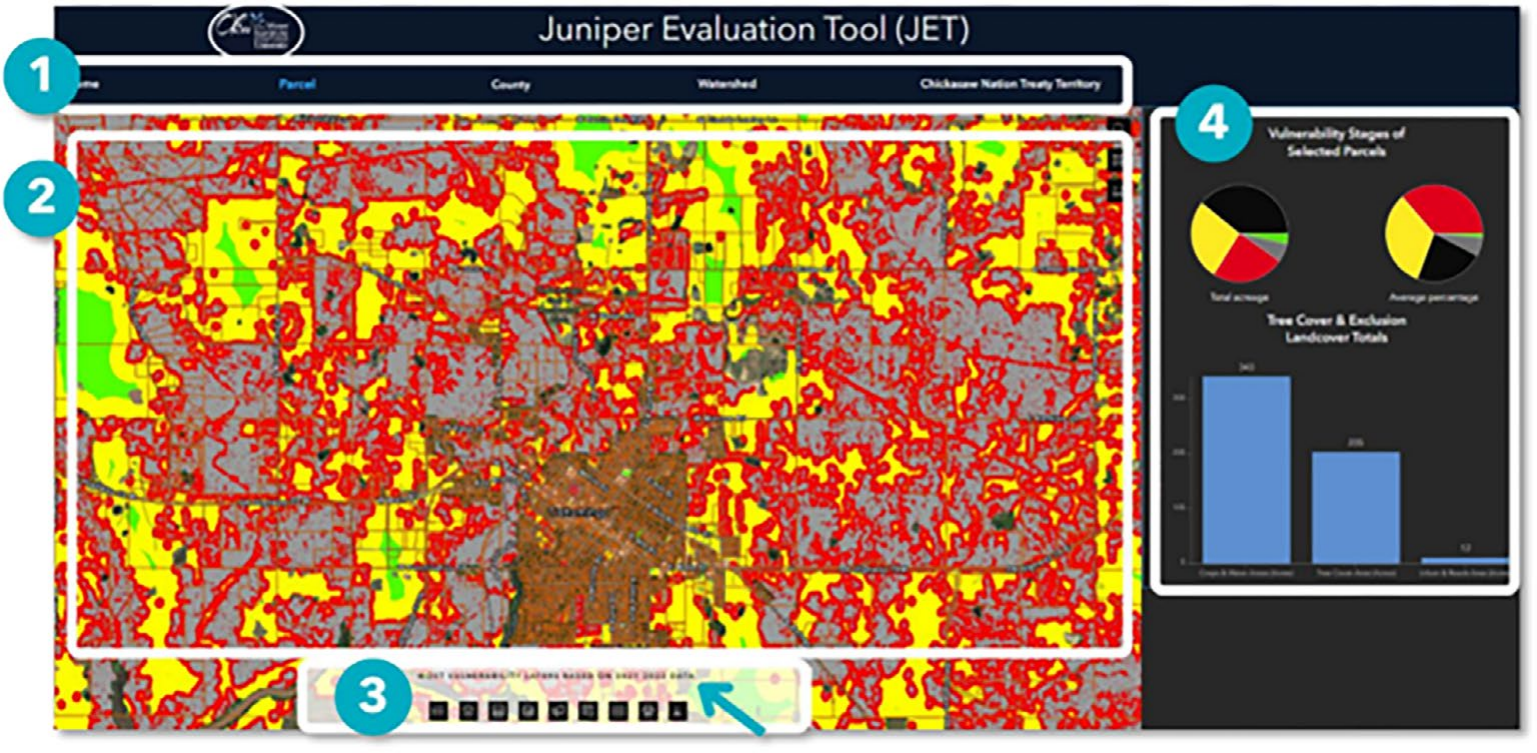
The JET Interface consisting of four parts. (1) The site navigation ribbon. (2) The map canvas. (3) The widget toolbox. (4) Charts.

**FIGURE 19:**
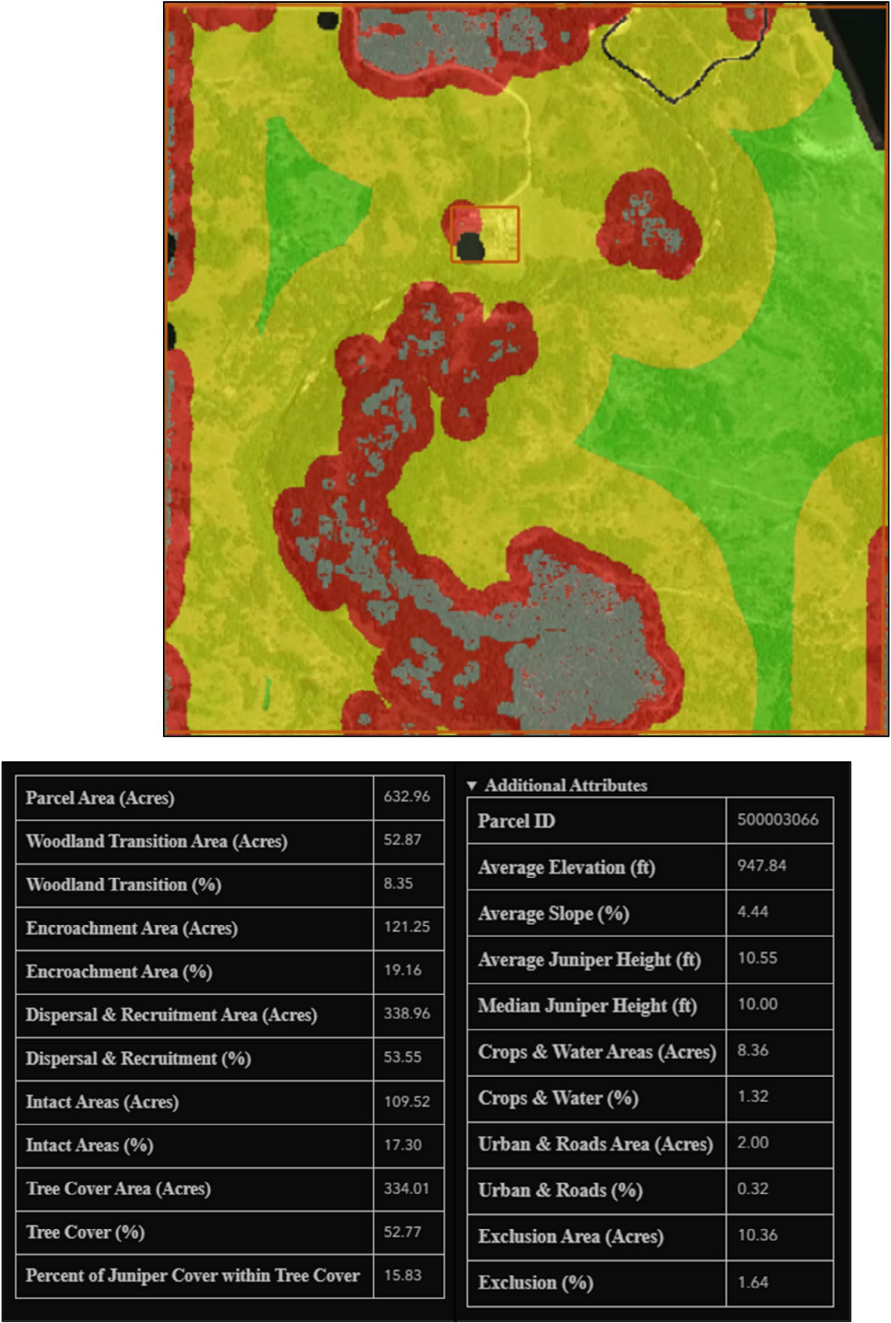
Attributes associated with each parcel, county, watershed and the CNTT.

The interface for the JET consists of four main parts (Figure 18):

1. The site navigation ribbon where Home, Parcel, County, Watershed or Chickasaw Nation Treaty Territory can be selected.
2. The map canvas, which displays the four vulnerability layers and the landscape level boundaries (i.e., Parcel, County, Watershed, or the Chickasaw Nation Treaty Territory).
3. The widget toolbox, located at the bottom of the map canvas. This is a container for several widgets, depending on which tab (i.e., Parcel, County, Watershed, or Chickasaw Nation Treaty Territory) the user is viewing. Available widgets include Legend, Map Layers, Attribute Table, Add Data, Select by Location, Select by Attributes, Swipe, Print Map, and Download Data.
4. Charts displaying the vulnerability stages of selected boundaries (i.e., pie charts in the top panel) and the tree cover and exclusion land cover totals (i.e., bar chart in the lower panel). A simple selection of a parcel on the map canvas will yield vulnerability information in the chart area to the right and provide the same information with other attributes in a table format (Figure 19).

More advanced tools for analysis are found in the widget toolbox and are explained in greater detail in the user's guide provided on the homepage of the JET (The Oka Institute Spatial Data Center, n.d.).

### 5.3 Model‐Based Tree and Juniper Cover Estimates

#### 5.3.1 Chickasaw Nation Treaty Territory and County Estimates

**FIGURE 20:**
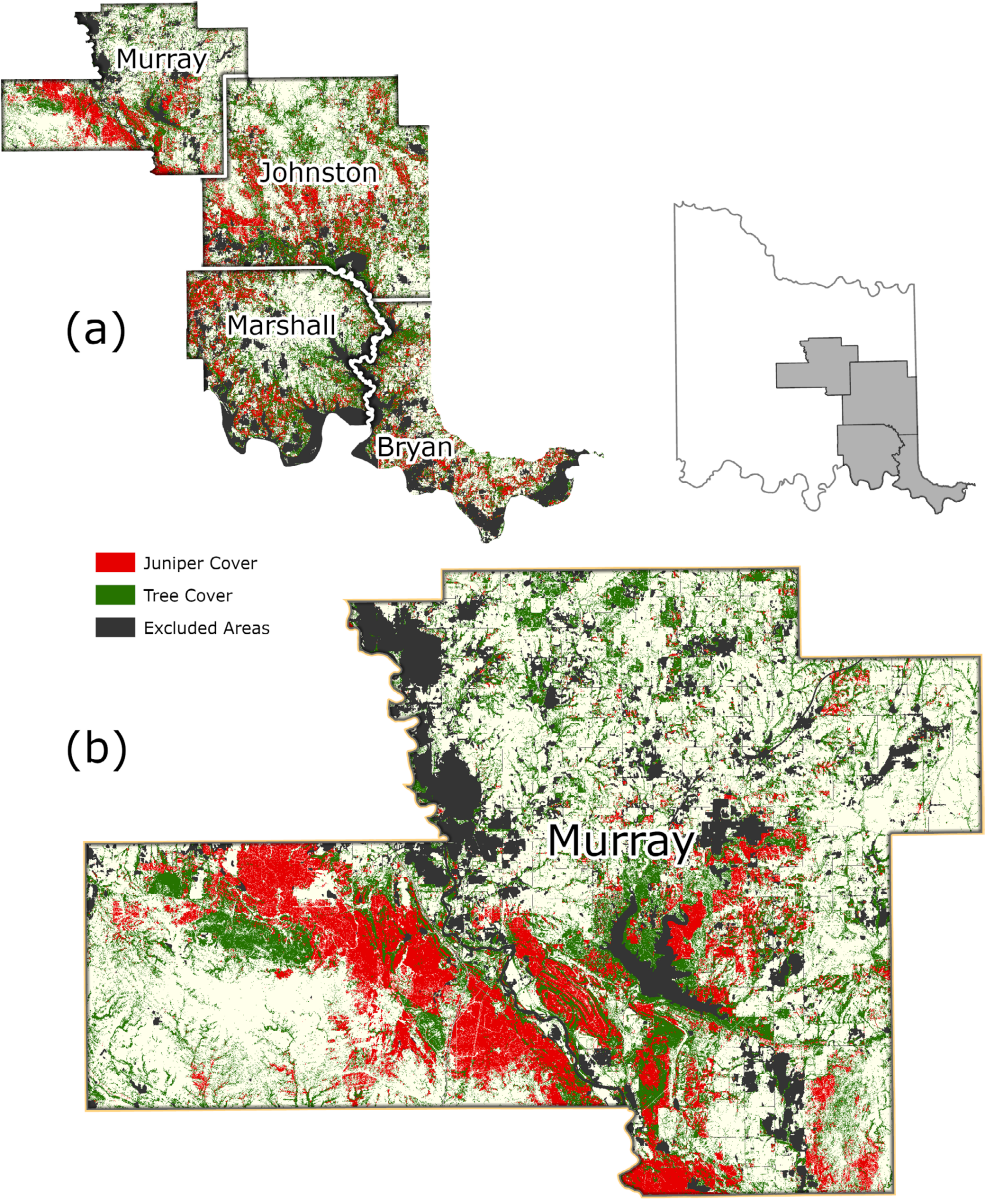
(a) Counties in the CNTT with a high percentage (> 30%) of juniper cover (red) with respect to tree cover (green) using the model output data. (b) Murray County is shown in greater detail. Areas shown in black represent features that were excluded from the model (water areas, crop land, roadways and urban areas). Inset map of the CNTT shows the locations of the four counties: Murray, Johnston, Marshall and Bryan.

**FIGURE 21:**
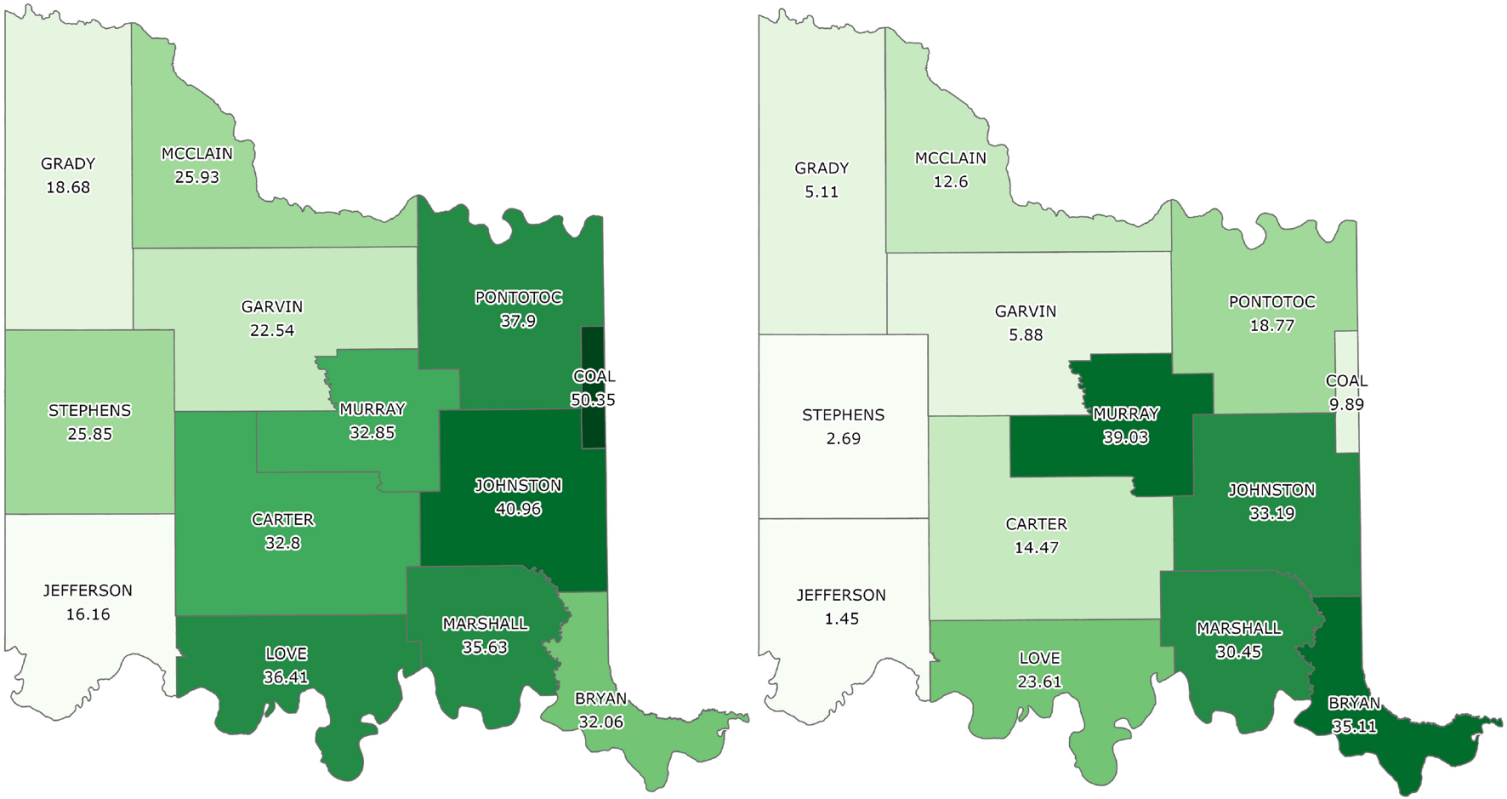
Percent tree cover by county area (left) and percent juniper cover as a percentage of tree cover area by county (right).

Based on the final model output (Figure 10), tree cover across the CNTT is approximately 29%, of which, approximately 18.4% is made up of juniper species, accounting for approximately 5.4% of the CNTT area (Table 10). In terms of acres of land lost to juniper species, this equates to approximately 255,145 acres of juniper tree species with major concentrations within the Arbuckle Mountains, Arbuckle Uplift, and Eastern Cross Timbers ecoregions. In addition, juniper tree species are also abundant in riparian areas, particularly in the drier areas in the eastern half of the study area. Of the 13 counties represented in the CNTT, Murray, Bryan, Johnston, and Marshall Counties have the highest concentrations (> 30%) of juniper species with respect to tree cover at 39.0%, 35.1%, 33.2%, and 30.5%, respectively (Table 10) with tree and juniper cover data from the JET shown in Figure 20. The two columns outlined in red in Table 10 are visually represented in Figure 21. As expected, more tree cover and a greater percentage of juniper cover with respect to tree cover are found in the eastern portion of the CNTT, following an increasing precipitation gradient from west to east (Oklahoma Climatological Survey 2024). For counties intersecting the CNTT boundary, only the county area within the CNTT is included in the analysis.

**TABLE 10:** Tree and Juniper Cover by County. Columns in red are mapped in Figure 21.

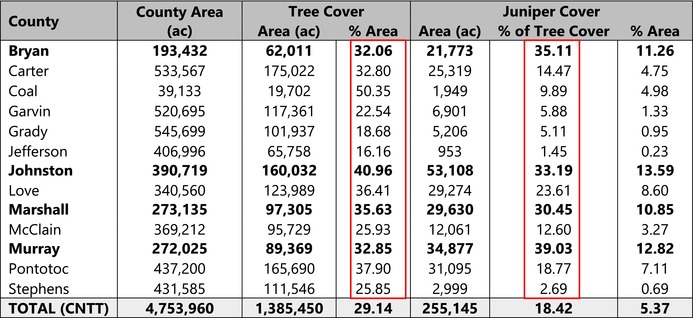

*Note:* Tree and Juniper Cover by County. Bold numbers in both red columns are mapped in Figure 20 and all numbers in the red columns are displayed in Figure 21.

#### 5.3.2 Watershed Level Estimates

**FIGURE 22:**
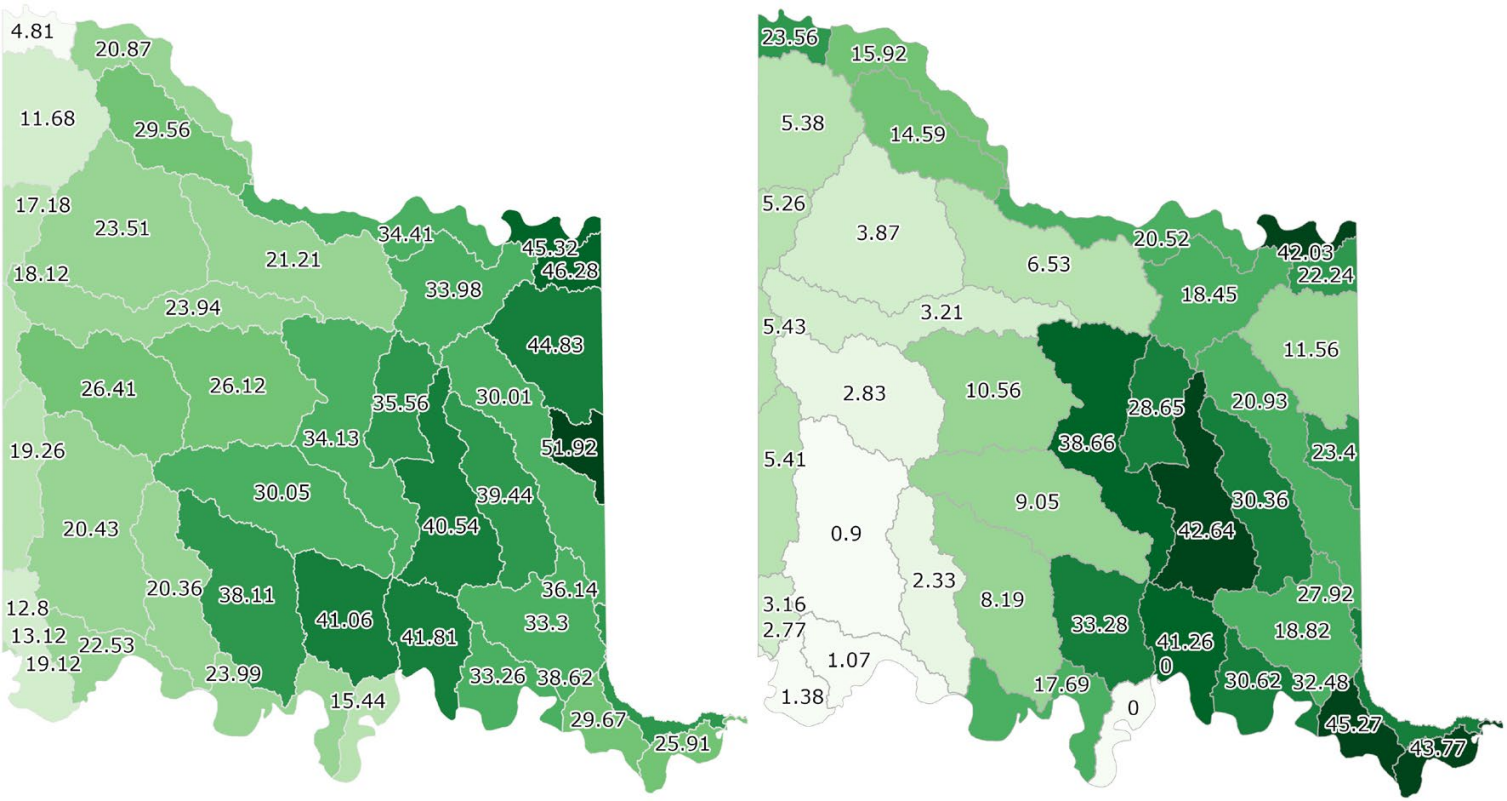
Percent tree cover by watershed area (left) and percent juniper cover as a percentage of tree cover area by watershed (right).

**FIGURE 23:**
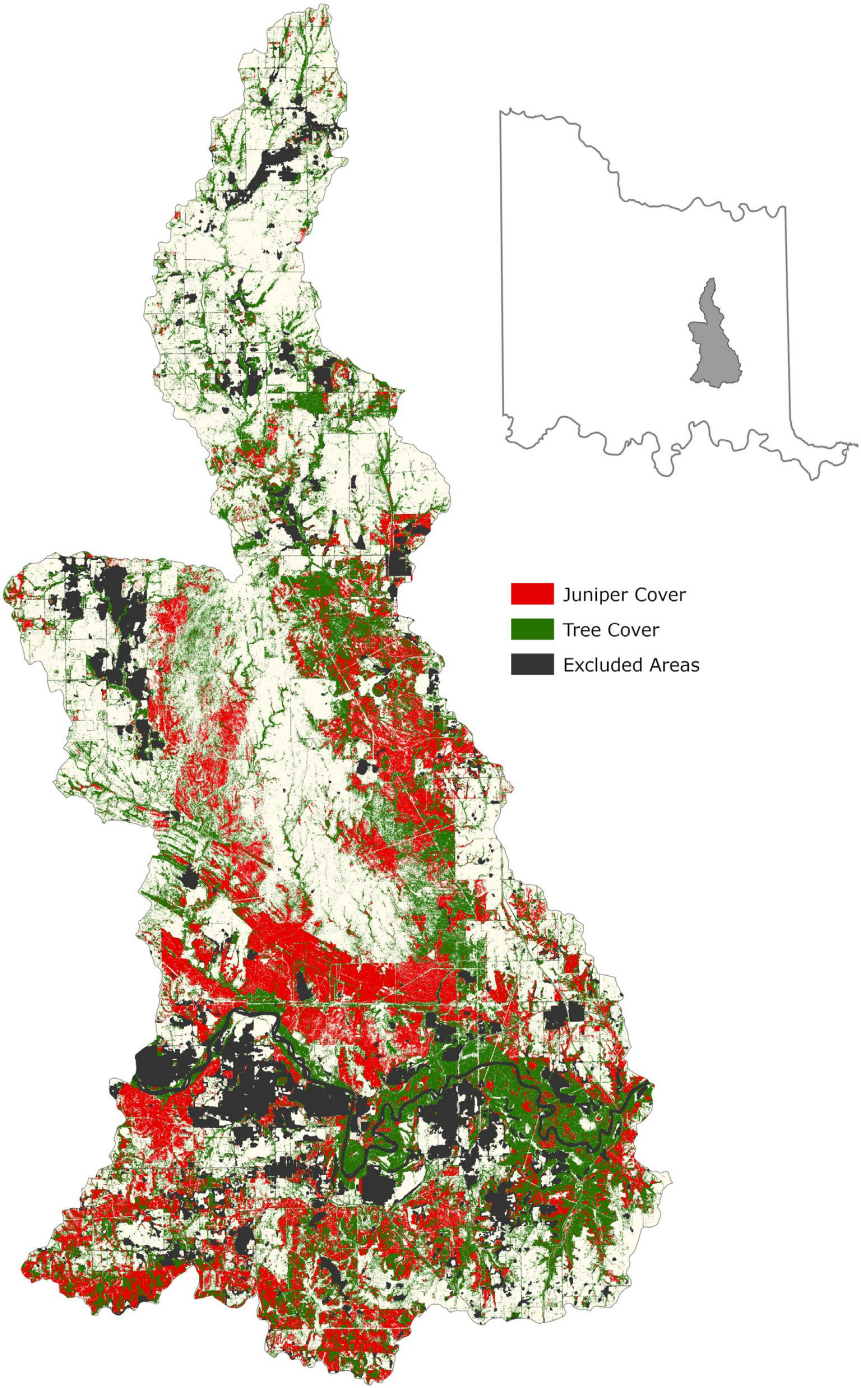
City of Ravia‐Washita River watershed showing juniper cover (red) with respect to tree cover (green) using the JET data. Areas shown in black represent features that were excluded from the model (water areas, crop land, roadways and urban areas). The inset map of the CNTT shows the location of the watershed.

There are 41 Hydrologic Unit Code 10 (HUC‐10) watersheds represented in the CNTT as shown in Table 11. Watersheds in bold contain more than 30% juniper cover with respect to tree cover; these are also shown in Figure 22. A sample watershed (i.e., City of Ravia‐Washita River) showing juniper cover, tree cover, and about 43% juniper cover with respect to tree cover from the JET is shown in Figure 23. Following a similar pattern as county data, more tree cover and a higher percentage of juniper cover with respect to tree cover are found in the eastern portion of the CNTT, following an increasing precipitation gradient from west to east (Oklahoma Climatological Survey 2024). For watersheds intersecting the CNTT boundary, only the area within the CNTT is included in the analysis.

**TABLE 11:** Tree and Juniper Cover by Alphabetized Watershed.

Watershed Name	Watershed area (ac)	Tree cover		Juniper cover		
		Area (ac)	% Area	Area (ac)	% of Tree Cover	% Area
Belknap Creek‐Red River	52,928	6946	13.12	96	1.38	0.18
Buggy Creek‐Canadian River	37,272	1791	4.81	422	23.56	1.13
Caddo Creek	224,273	67,398	30.05	6101	9.05	2.72
Canadian Sandy Creek‐Canadian R.	145,311	49,379	33.98	9112	18.45	6.27
City of Chickasha‐Washita River	160,177	18,705	11.68	1006	5.38	0.63
**City of Davis‐Washita River**	**214,514**	**73,203**	**34.13**	**28,301**	**38.66**	**13.19**
**City of Ravia‐Washita River**	**170,022**	**68,928**	**40.54**	**29,393**	**42.64**	**17.29**
City of Tuttle‐Canadian River	94,374	19,700	20.87	3136	15.92	3.32
Cow Creek	94,349	18,173	19.26	983	5.41	1.04
**Denison Dam‐Red River**	**81,137**	**26,988**	**33.26**	**8264**	**30.62**	10.18
**Fobb Bottom‐Red River**	**103,060**	**43,087**	**41.81**	**17,779**	**41.26**	**17.25**
Headwaters Muddy Boggy Creek	38,307	17,728	46.28	3943	22.24	10.29
Horseshoe Bend‐Red River	170	26	15.44	0	0.00	0
**Island Bayou**	**27,645**	**10,677**	**38.62**	**3468**	**32.48**	**12.54**
**Lake Konawa‐Canadian River**	**33,393**	**15,133**	**45.32**	**6361**	**42.03**	**19.05**
**Lake Murray**	**136,222**	**55,936**	**41.06**	**18,615**	**33.28**	**13.67**
Lake Nocona‐Red River	86,195	19,417	22.53	208	1.07	0.24
Lake of the Arbuckles	88,581	31,500	35.56	9026	28.65	10.19
Lake Texhoma‐Washita River	160,035	53,293	33.3	10,032	18.82	6.27
Little Beaver Creek	27,723	5023	18.12	273	5.43	0.98
Little Washita River	45,438	7805	17.18	410	5.26	0.9
Lower Beaver Creek	34,730	4444	12.8	141	3.16	0.4
Lower Blue River	5036	1820	36.14	508	27.92	10.09
Lower Mud Creek	135,861	27,658	20.36	643	2.33	0.47
Lower Wildhorse Creek	203,504	53,153	26.12	5614	10.56	2.76
Middle Clear Boggy Creek	43,911	22,798	51.92	5334	23.40	12.15
Moss Lake‐Red River	98,223	23,560	23.99	4168	17.69	4.24
Rush Creek	166,558	39,880	23.94	1278	3.21	0.77
**Tishomingo NWR‐Washita River**	**137,835**	**54,357**	**39.44**	**16,503**	**30.36**	**11.97**
**Town of Colbert‐Red River**	**56,537**	**16,777**	**29.67**	**7595**	**45.27**	**13.43**
**Town of Kemp‐Red River**	**40,979**	**10,619**	**25.91**	**4647**	**43.77**	**11.34**
Town of Lindsey‐Washita River	278,398	65,460	23.51	2531	3.87	0.91
Town of Maysville‐Washita River	228,170	48,397	21.21	3159	6.53	1.38
Town of Wanette‐Canadian River	100,932	34,735	34.41	7127	20.52	7.06
Upper Blue River	184,837	55,471	30.01	11,612	20.93	6.28
Upper Clear Boggy Creek	159,157	71,353	44.83	8246	11.56	5.18
Upper Mud Creek	279,353	57,061	20.43	516	0.90	0.18
Upper Wildhorse Creek	200,976	53,085	26.41	1502	2.83	0.75
Walnut Bayou Creek	213,470	81,359	38.11	6664	8.19	3.12
Walnut Creek‐Canadian River	129,850	38,388	29.56	5602	14.59	4.31
Whiskey Creek‐Red River	1445	276	19.12	8	2.77	0.53

*Note:* Tree and Juniper Cover by Alphabetized Watershed. Rows in bold represent watersheds that contain more than 30% juniper cover with respect to tree cover.

#### 5.3.3 Parcel Level Estimates

**FIGURE 24:**
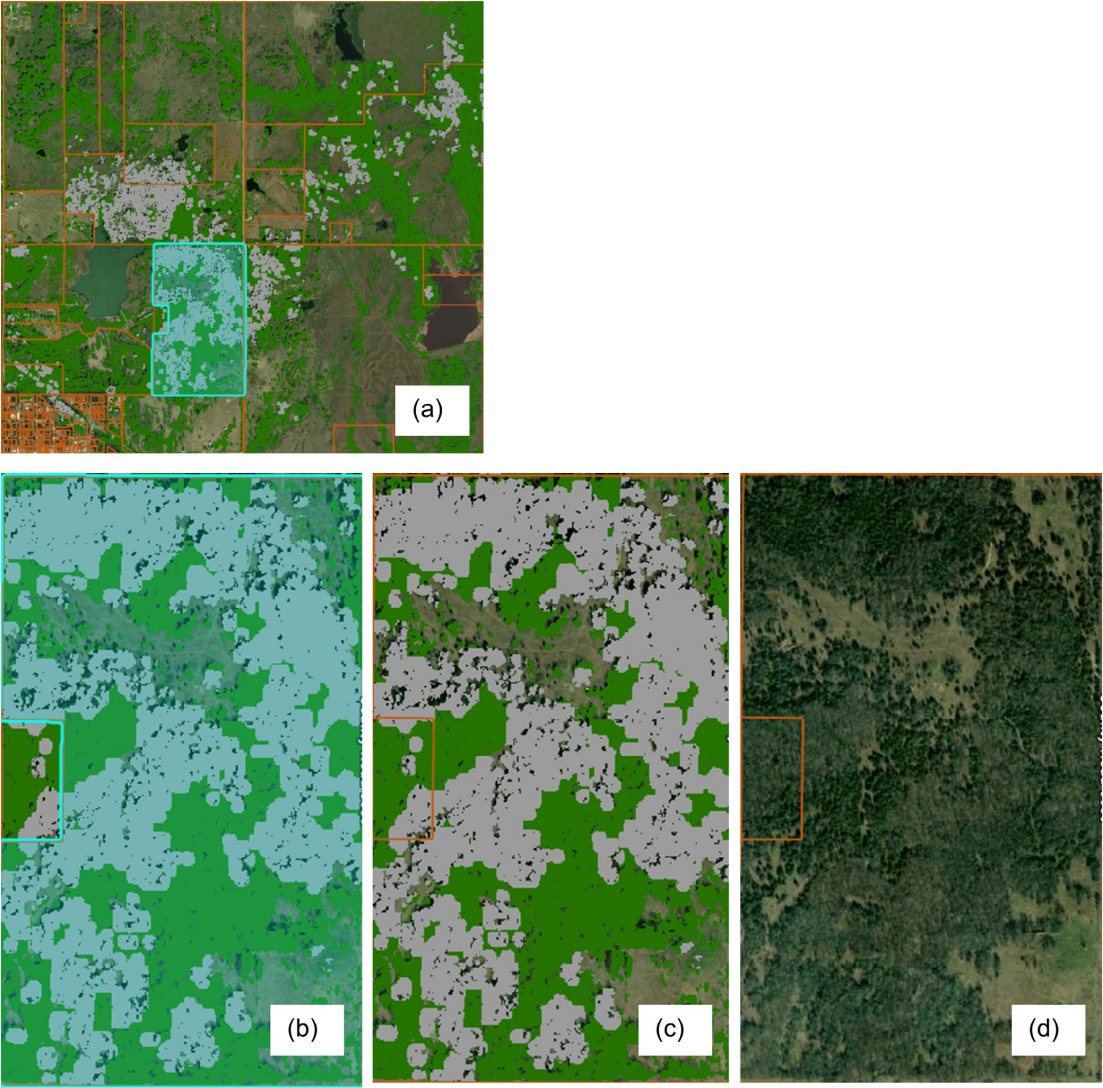
Parcel ID 620001462 selection using the JET‐ (a) showing tree cover in green and woodland transition (juniper distribution) in gray, (b) Closer view of the highlighted parcel, (c) Tree cover and woodland transition raster layers displayed, and (d) Parcel with no raster layers displayed.

There are 247,567 parcels represented in the JET. An example parcel is shown in Figure 24 and the associated attributes for tree cover and juniper cover are shown in Table 12. This parcel is 146 acres in size and contains 113 acres (77%) of tree canopy cover (TCC) and 66 acres (45%) of juniper canopy cover, with 58% of the tree cover classified as juniper.

**TABLE 12:** Tree and Juniper Cover for an example parcel.

Parcel ID	Parcel(s) area (ac)	Tree Cover		Juniper cover		
		Area (ac)	% Area	Area (ac)	% of Tree Cover	% Area
620,001,462	146	113	77	66	58	45

## 6 Discussion

The JET was launched in 2025 as a public, science‐based decision‐support tool for the Chickasaw Nation in south‐central Oklahoma. This tool was created from a species‐focused juniper evaluation model to provide landowners, land managers, resource managers, and state and federal support staff with various parcel‐level metrics needed to access juniper encroachment for the purpose of targeted and parcel‐level management for watershed conservation within the CNTT. The initial goal of the project was to produce juniper extent and vulnerability information that could be integrated into the existing RAP tool as value‐added species‐specific data layers to make the existing tree cover data more robust. However, this integration proved to be infeasible, so the JET was created as a separate ArcGIS‐based outward‐facing public tool. The approach and methodology underlying the JET were based partly on the Rangeland Analysis Platform methodology (Rangeland Analysis Platform, n.d.), the Woody Encroachment Guide (Twidwell et al. 2021), and NRCS’s support of a tool that would be used in concert with the RAP and the RaBET.

As with the other tools described in this article, the JET is free to use, science‐based, scalable, and actionable. The JET also uses open datasets readily available throughout the United States. Unlike the other tools, however, the JET is built using a stepwise masking and thresholding methodology that is simple and can be accomplished within the GEE platform with minimal data pre‐processing and associated staff time. The open‐source nature of the JET will allow it to be updated, altered and improved as future datasets become available to provide (1) landscape changes in time and space, (2) higher accuracy results, and (3) science‐driven planning for management purposes over larger geographic areas. Its open‐source methodology will allow the JET to serve as a springboard for future state‐wide and potential regional expansion.

### 6.1 Accuracy Assessment

The producer's accuracy for the JET confirms that “lone” and “edge” juniper pixels are least likely to be identified by the model. The user's accuracy for all classes is high and confirms that the final model output represents juniper trees on the ground, regardless of validation point class. Also, the trend in overall accuracies is expected, as “lone” and “edge” juniper trees and small trees in groups may not be accurately classified or not classified at all due to the relatively large pixel size of the Sentinel‐2 dataset (Starks et al. 2011; Coates et al. 2017; Falkowski et al. 2017; Gustafson et al. 2018; Roth et al. 2021), which was used to identify juniper trees in the model after using the one‐meter lidar‐derived canopy layer and NAIP aerial photography. Conversely, the “group” and “stand” accuracies are much higher as the combined canopy crowns are larger and more easily mapped by 10‐m satellite data. Similarly, the lower kappa coefficients for “lone” and “edge” juniper trees compared to the higher kappa coefficient for “group” and “stand” juniper trees validates a “substantial agreement” for the model (Landis and Koch 1977). Because the model is aimed at guiding parcel‐level management for watershed conservation planning, rather than identifying individual trees, these accuracy metrics can be considered acceptable.

### 6.2 Tree Cover Distribution Estimates

Although tree cover (Figure 9) was not the main output of the JET, it deserves some mention as it was essential for mapping juniper extent (Figure 10). While other methods and products exist for generating tree cover, they either lack the simplicity or level of positional accuracy that the JET's stepwise model provides. For instance, a comparison was made between the tree cover canopy classification results from the Rangeland Analysis Platform (Rangeland Analysis Platform, n.d.) and the tree canopy layer from the JET model for all counties and the overall study area (Table 13). Tree canopy cover estimates derived from the RAP and the JET differed significantly across the 13‐county comparison (paired *t*‐test, *t* (12) = −3.87, *p* = 0.002). On average, the JET reported higher tree cover percentages than the RAP and this may reflect differences in data sources and dates, pixel averaging and spatial resolution, masking techniques, and classification methods used by each platform. See Tree Cover Distribution Estimates in the Supporting Information for comparisons to other tree cover data sets.

**TABLE 13:** Percent tree cover (2021–2022) from the RAP and JET and percent of JET tree cover above or below the RAP tree cover estimates by county in the CNTT. Data for the RAP was obtained from https://rangelands.app/ using shapefiles uploaded for each county.

County	2021–2022 Tree cover		Percent of JET tree cover above (+) or below (−) RAP tree cover percent
	RAP (% Area)	JET (% Area)	
Garvin	23.64	22.54	−1.10
Bryan	32.73	32.06	−0.66
Marshall	35.08	35.63	+0.56
Grady	17.43	18.68	+1.26
Jefferson	14.19	16.16	+1.97
Stephens	23.25	25.85	+2.60
Pontotoc	34.47	37.9	+3.44
Love	32.87	36.41	+3.54
McClain	21.70	25.93	+4.23
Carter	27.86	32.8	+4.95
Murray	25.72	32.85	+7.13
Johnston	32.24	40.96	+8.73
Coal	41.46	50.35	+8.90
CNTT	26.48	29.14	+2.67

### 6.3 Juniper Cover Distribution Estimates

The juniper filtering process using Sentinel‐2 leaf‐off satellite imagery produced the final juniper extent from the previously established NAIP‐derived tree cover filtering data (Figure 10). This filtering and thresholding process, although not as accurate as object‐based image classification and wavelet analysis for mapping trees (Coates et al. 2017; Falkowski et al. 2017; Gustafson et al. 2018), proved to be simpler and less time‐consuming, while still meeting the objective of parcel‐level management for watershed conservation planning within the CNTT. Other classification methods to classify evergreen species were considered, but it was not possible to fully integrate these higher‐order image classification techniques into a GEE workflow (Google Earth Engine Developers 2023; Pérez‐Cutillas et al. 2023) and fit within the guidelines of the original project objectives (Natural Resources Conservation Service, n.d.).

**FIGURE 25:**
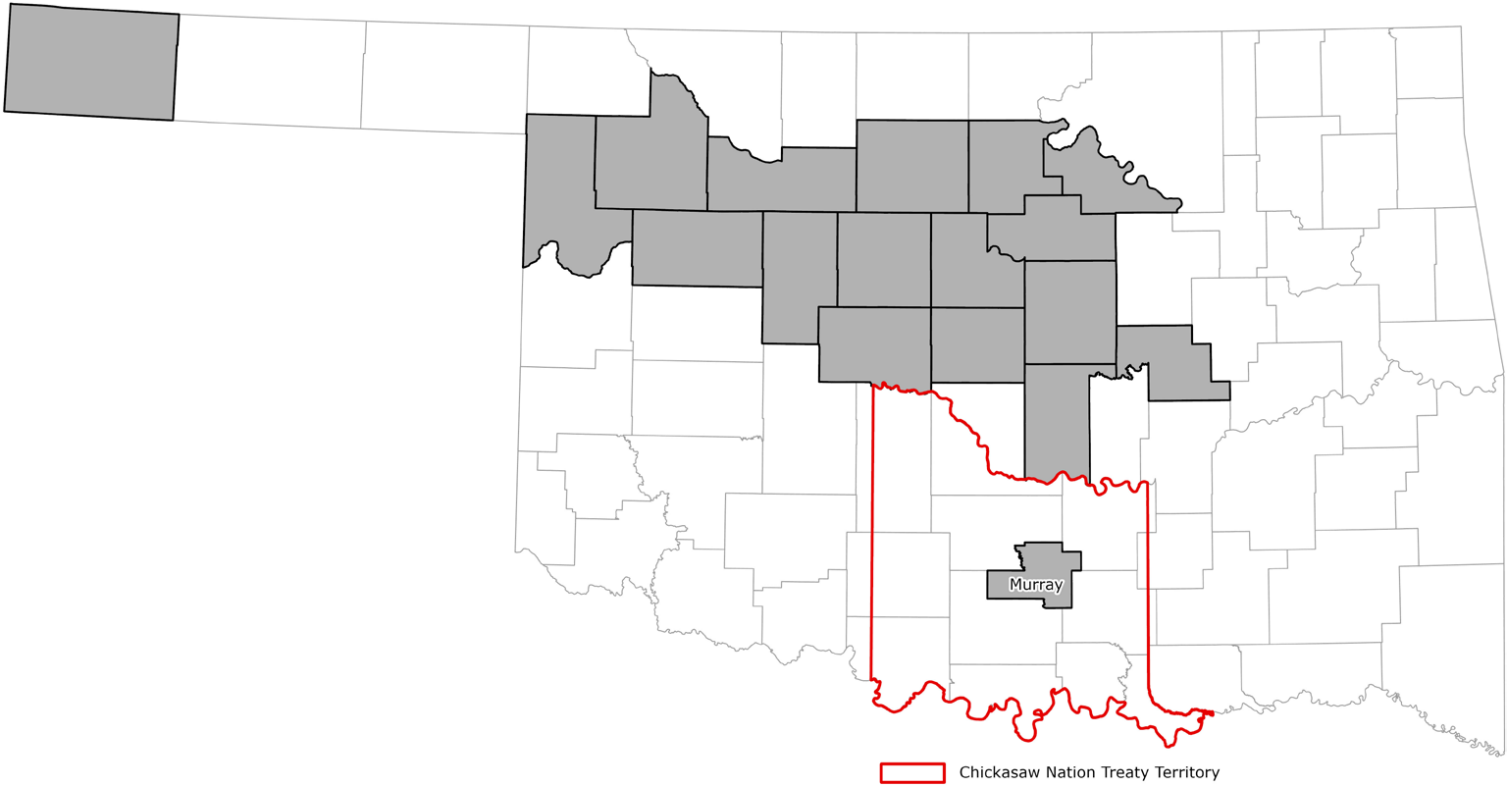
The county‐wide NRCS Red Cedar Mapping Project (2002–2008) included 18 counties, including Murray County in the CNTT.

Unfortunately, there is little to no current juniper species‐level data available to compare the JET model results against. The most comprehensive county‐wide juniper cover estimate to date was carried out under the Red Cedar Mapping Project by the NRCS and funded through the Oklahoma Department of Agriculture, Food and Forestry and the Oklahoma Department of Commerce in the early 2000s (United States Department of Agriculture, Natural Resources Conservation Service 2017). Of the 77 counties in Oklahoma, 18 were mapped (Figure 25) using Landsat‐5 imagery (i.e., 2004, 2005 and 2008 data) for 12 counties and Landsat‐7 imagery (i.e., 2002, 2005 and 2006 data) for 6 counties. The intended use of the early 2000s mapping project was to assess the concentration and spatial coverage of eastern redcedar ( *Juniperus virginiana* ) and Ashe juniper ( *Juniperus ashei* ), based on approximate canopy cover.

**FIGURE 26:**
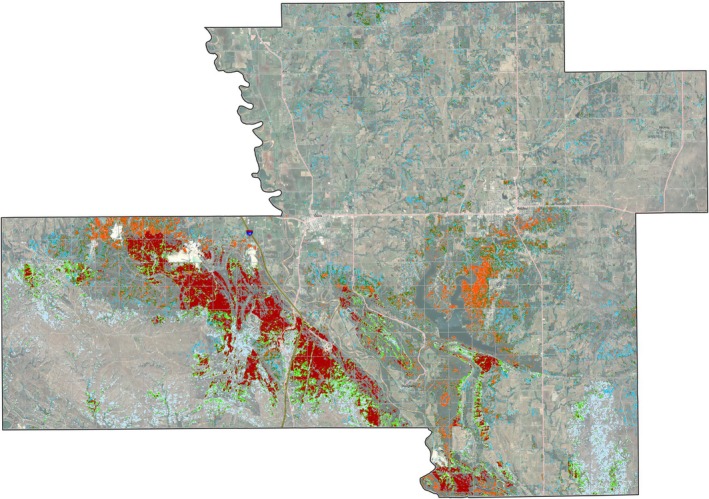
Juniper tree species encroachment in Murray County, OK. Higher concentrations of eastern redcedar are shown in orange and dark green and higher concentrations of Ashe juniper are shown in maroon and light green. The maps display the spatial relationship of different canopy densities occurring in each county. The three legend classes used for all maps include the following: (1) ≥ 70%—orange, (2) 30%–70%—dark green, and (3) 10%–30%—light blue. Murray County was the only county that fell within the CNTT and both eastern redcedar (Juniperus virginiana) and Ashe juniper (Juniperus ashei) were mapped using September 2005 Landsat‐7 imagery. Source: NRCS County Surveys found on an archive webservice: https://web.archive.org/web/20170602061233/https:/www.nrcs.usda.gov/wps/portal/nrcs/detail/ok/technical/dma/gis/?cid=nrcs142p2_000527.

**FIGURE 27:**
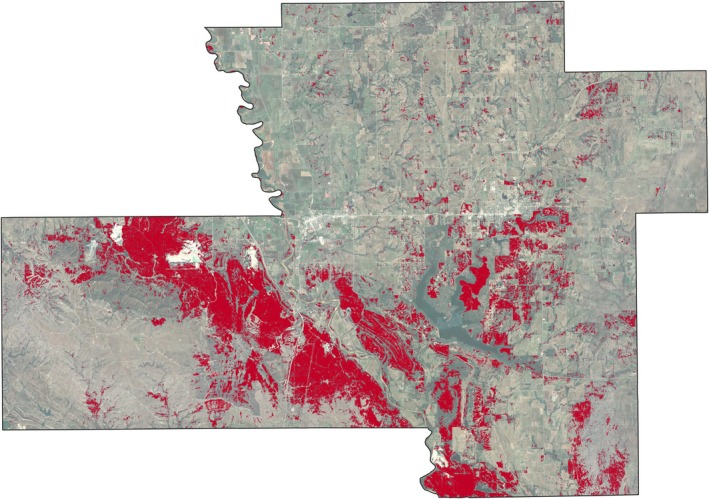
Juniper tree species encroachment in Murray County, OK. Source: Model output (2022) from the JET.

**FIGURE 28:**
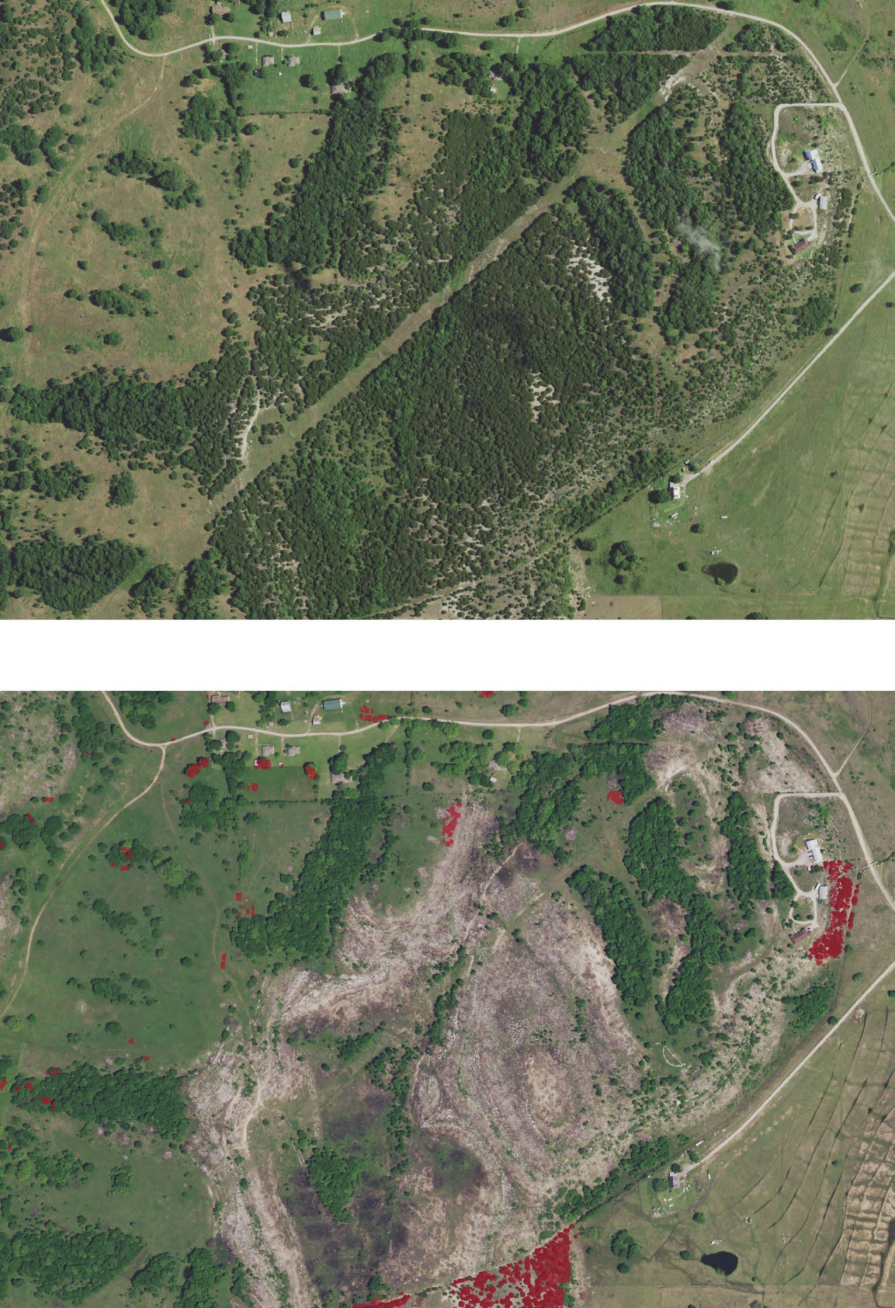
Targeted juniper removal between 2019 (top) and 2021 (bottom), as shown in NAIP photography. The modeled juniper tree species distribution is shown in red on the 2021 image.

Overall, there is good visual agreement between the NRCS‐produced juniper species maps and the woodland transition produced by the model and displayed in the JET (Figures 26 and 27). Some areas of difference in the presence of juniper species are visible, and these are likely due to prescribed burns and/or mechanical removal BMPs. These are located in particular between the Lake of the Arbuckles and the City of Sulfur. An example of an area where juniper tree removal has been prioritized by the CN, south of the Lake of the Arbuckles, is shown in Figure 28. It should be noted that the digital data from the NRCS‐produced juniper species maps from the early 2000s are not currently available, so no quantitative comparison was made.

### 6.4 Juniper Vulnerability Stages and the JET Functionality

The final output from the juniper tree species distribution model (i.e., juniper extent or woodland transition) was the basis for the creation of the juniper vulnerability stages in the JET. The stages (Table 6 and Figure 15) are correlated with the Woody Encroachment Vulnerability Guide (Twidwell et al. 2021), which explains the risk and vulnerability facing the Great Plains, of which south central Oklahoma is a part. This guide also provides strategies for proactively managing woody encroachment and addresses the shortcomings of the existing rangeland management paradigm by promoting the idea of “protecting the core,” or intact areas (i.e., areas without woody encroachment), to stop further woody encroachment, rather than prioritizing restoring areas that have already undergone a woodland transition (Twidwell et al. 2013; Maestas et al. 2022). Moving from a reactive to a proactive approach shifts the paradigm from spotted removal (i.e., a “shotgun” approach) to a continual process of reinforcing and enlarging functional grasslands. The proposed strategy is to prioritize the defense and growth of these “core” areas involving boundary management to safeguard intact areas and grasslands against further woody encroachment and to ensure the resilience and sustainability of these landscapes (Twidwell et al. 2021).

**FIGURE 29:**
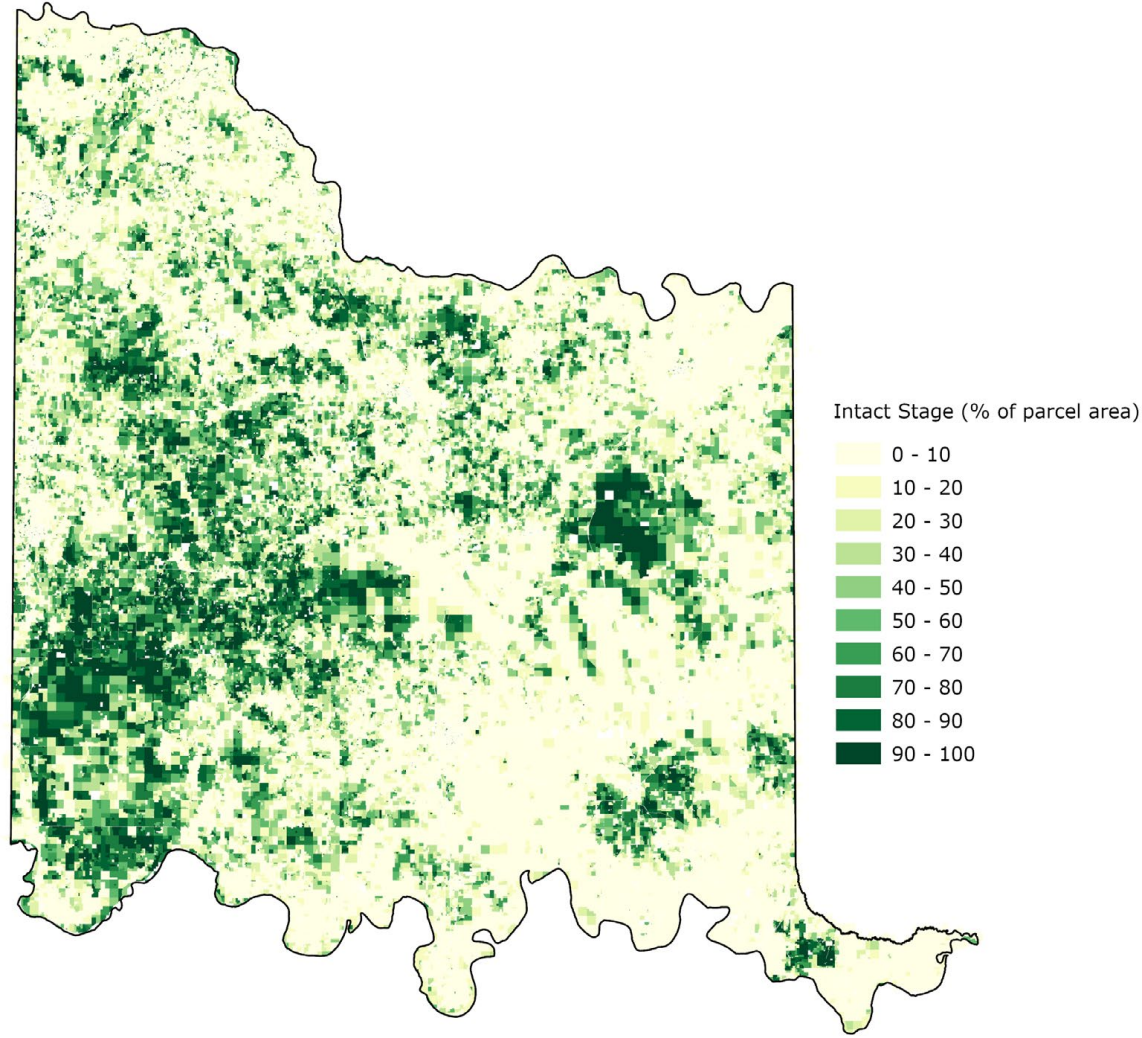
Intact area by parcel, showing core areas of defense against potential woody encroachment.

**FIGURE 30:**
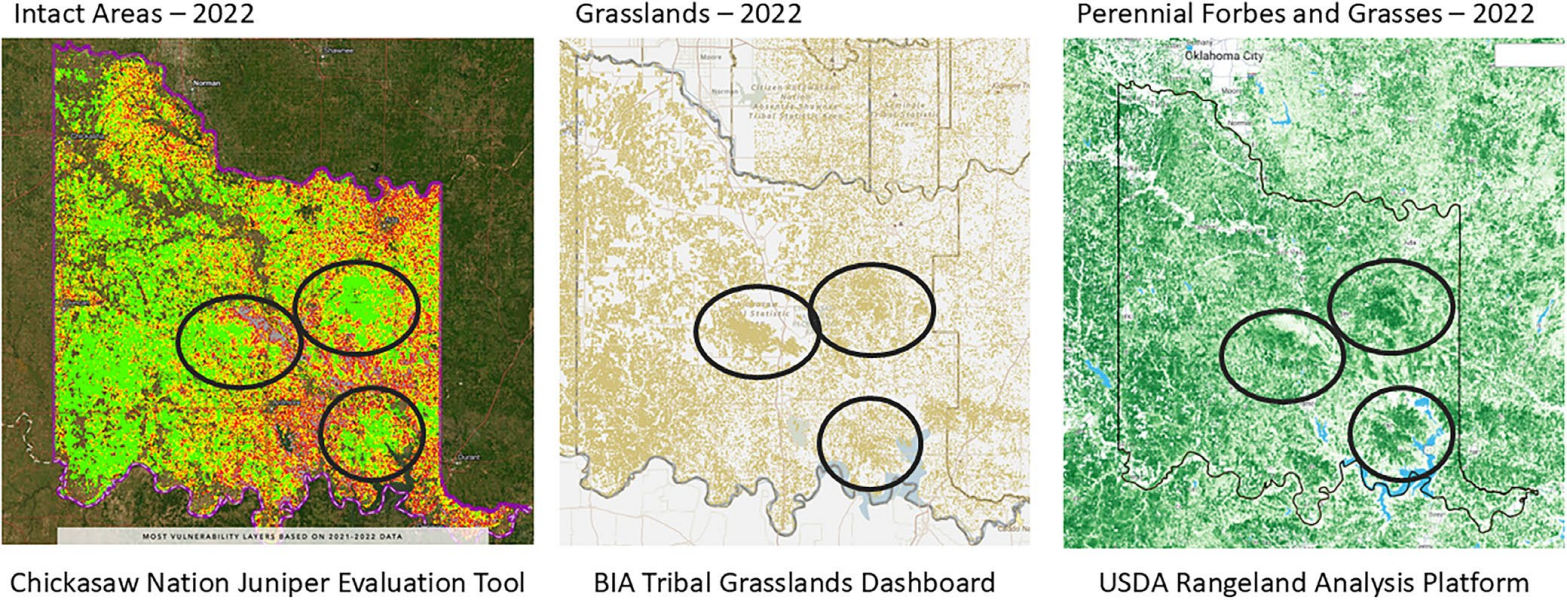
Intact areas from the JET compared to grassland areas mapped by the BIA and the NRCS, showing core areas of defense from potential woody encroachment.

A similar approach is being adopted throughout the CNTT by using the JET to prioritize lands still undergoing a woodland transition and expanding/protecting intact areas. Figure 29 shows “core” areas from the JET to display intact areas within each parcel that are mostly devoid of juniper tree species (i.e., darker green colors). This also visually correlates with other tools such as the Grassland on Tribal Lands dashboard (Bureau of Indian Affairs, Branch of Geospatial Support 2025) and the Rangeland Analysis Platform (Rangeland Analysis Platform, n.d.) (Figure 30), with the JET providing a higher level of detail. A display of intact areas using the JET (i.e., light green color) is a very straightforward exercise, as vulnerability layers are automatically visible on the JET map canvas (Figure 18).

In general, when used for encroachment control, light green (i.e., intact areas) and yellow areas (i.e., dispersal and recruitment) in the JET would be more conducive to prescribed fire, whereas red areas (i.e., encroachment) would be better suited to mechanical removal methods, followed by pile burning and/or prescribed fire. To protect the ecological integrity and economic productivity of rangeland and grasslands in the CNTT, prescribed fire has been a popular management practice by landowners in Oklahoma (Stevens et al. 1997; Weir 2010; Bidwell et al. 2021; Stroman et al. 2020). Overall, Oklahoma is one of the most active states in the use of this land management practice (Melvin 2018) and according to research from the Oklahoma State University Extension, about “2.5 million acres, or 6% of the total land area, of native prairie, shrubland, and forest are burned in Oklahoma each year (Bidwell et al. 2021).” It is well known that fire can mitigate woody encroachment, including juniper tree species, and improve rangeland and grassland productivity through nutrient and water cycling (Twidwell et al. 2013; Hmielowski 2014; Stroman et al. 2020; Bidwell et al. 2021). In fact, First American peoples intentionally made use of fire to stimulate vegetation growth and keep woodlands and the understory of forested areas open (Stambaugh et al. 2009; DeSantis, Hallgren, and Stahle 2010; Hallgren et al. 2012; Roos et al. 2018; Bidwell et al. 2021) and today is no different. Additionally, the native plant community in Oklahoma has adapted to treatment by periodic fire, and some plants even require fire to complete their lifecycles (DeSantis, Hallgren, and Stahle 2010; Bidwell et al. 2021).

It has been shown that tree removal can produce adverse short‐term ecological effects when site resilience is low, ground cover is inadequate, or removal methods exacerbate disturbance as impacts vary with the removal method, ecological site characteristics, vegetation composition, and disturbance history (Miller et al. 2013; Pierson et al. 2015). Knowing site‐specific ecological factors in which to tailor tree removal that optimizes vegetation recovery and minimizes runoff and erosion risks can elevate successful management. For example, Ricca et al. (2018) demonstrated that spatial data can be used to classify the landscape based on indices of ecological resilience and resistance to prioritize restoration in areas for Sage‐grouse use and avoid costly investments in other areas that may be prone to restoration failure. Coates et al. (2019) demonstrated the importance of integrating spatiotemporal habitat indices for better site‐specific management practices for the greater sage‐grouse. Additionally, the National Park Service is using a resilience‐based framework in the management of grassland ecosystems threatened by woody encroachment (Ling et al. 2023). The JET, in its inaugural version, provides the initial step in reclaiming rangeland and grassland productivity at the expense of encroaching juniper trees, with future versions of the JET planned to focus on adding ecological data for more site‐specific BMPs.

**FIGURE 31:**
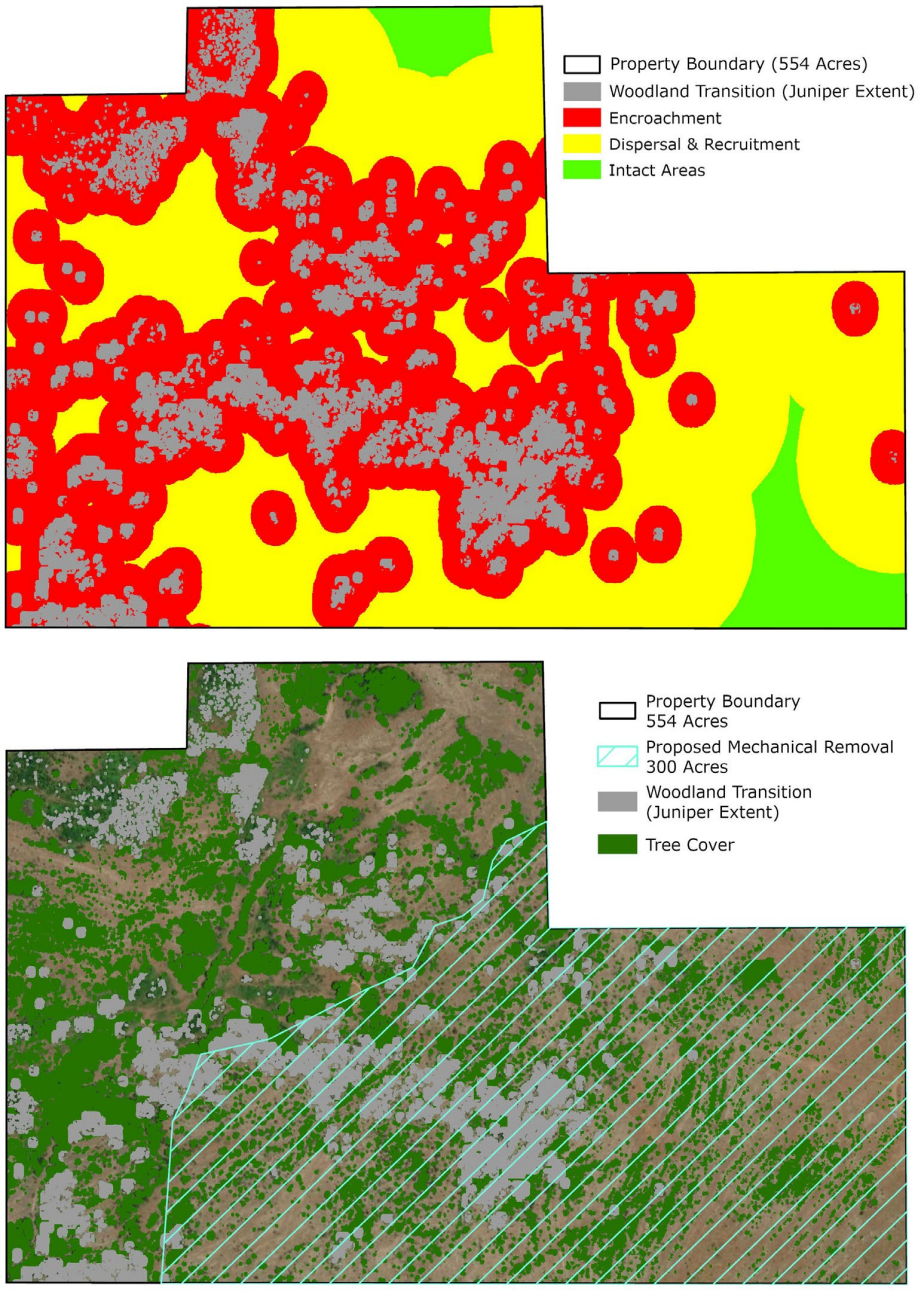
The JET is used on a 554‐acre property to assess its current vulnerability (top image—vulnerability layers) and then a preliminary plan for 300 acres of mechanical removal was produced (bottom image—green hatched area) followed by pile burning and prescribed fire.

Resource managers in the CN are utilizing the JET to efficiently collaborate with landowners in the implementation of prescribed fire and mechanical removal treatments under the Reserved Treaty Rights Lands (RTRL) program (Bureau of Indian Affairs 2015, 2025). The JET enables managers to rapidly assess parcel‐level metrics through integrated tools such as the *Select by Location* and *Attribute Table* widgets. These data can be exported into tabular formats for further analysis or mapped for field use. Figure 31 exemplifies the JET being used on a 554‐acre property to assess its tree and juniper cover and juniper vulnerability so that a preliminary management plan, after a landowner visit, can be produced. The JET shows about 50% of the property in the encroachment stage (i.e., red color) and about 30% in the dispersal and recruitment stage (i.e., yellow color). With dense areas of tree and juniper cover and steep topography in the northwestern half of the property, attention was turned to the southeastern half. With a significant number of juniper trees on the property (i.e., gray color), along with heavy encroachment (i.e., red color), it was determined to first use mechanical treatment across the southeastern 300 acres and then return for pile burning and prescribed fire in the more open areas for boundary control so that physical and financial resources were used efficiently. This functionality of the JET streamlines the development of preliminary management plans and prioritizes field visits, often reducing the duration and number of required landowner visits by 2 to 4 days, thereby increasing the effectiveness of conservation planning and implementation. Similar functionality can be used to mitigate wildfire risk and protect culturally significant structures.

The Chickasaw Nation is also collaborating with local watershed organizations, including the Lake of the Arbuckles Watershed Association (LAWA) and the Blue River Foundation, to advance conservation planning and best management practices (BMPs) within the Lake of the Arbuckles and Upper Blue River watersheds. The vulnerability data and widgets in the JET are being used to identify areas that may be experiencing a decline in favorable ecological composition due to varying degrees of woody encroachment and areas listed as impaired waters under section 303 (d) of the Clean Water Act by the U.S. Environmental Protection Agency. This targeted conservation strategy applies a watershed‐based framework to identify critical source areas (CSAs) for intervention (Meals et al. 2012), in contrast to the reactive “shotgun” approach commonly used in the former paradigm (Twidwell et al. 2021). White et al. (2009) demonstrated that a small proportion of targeted areas within watersheds contributes disproportionately to overall pollutant loads, underscoring the importance of this approach. Despite the demonstrated effectiveness of CSA‐based targeting, many state and federal conservation programs have yet to fully implement it. The CN is committed to advancing proactive, data‐driven conservation planning that integrates watershed‐scale analysis with on‐the‐ground management to improve water quality, restore habitat, and build ecological resilience.

**FIGURE 32:**
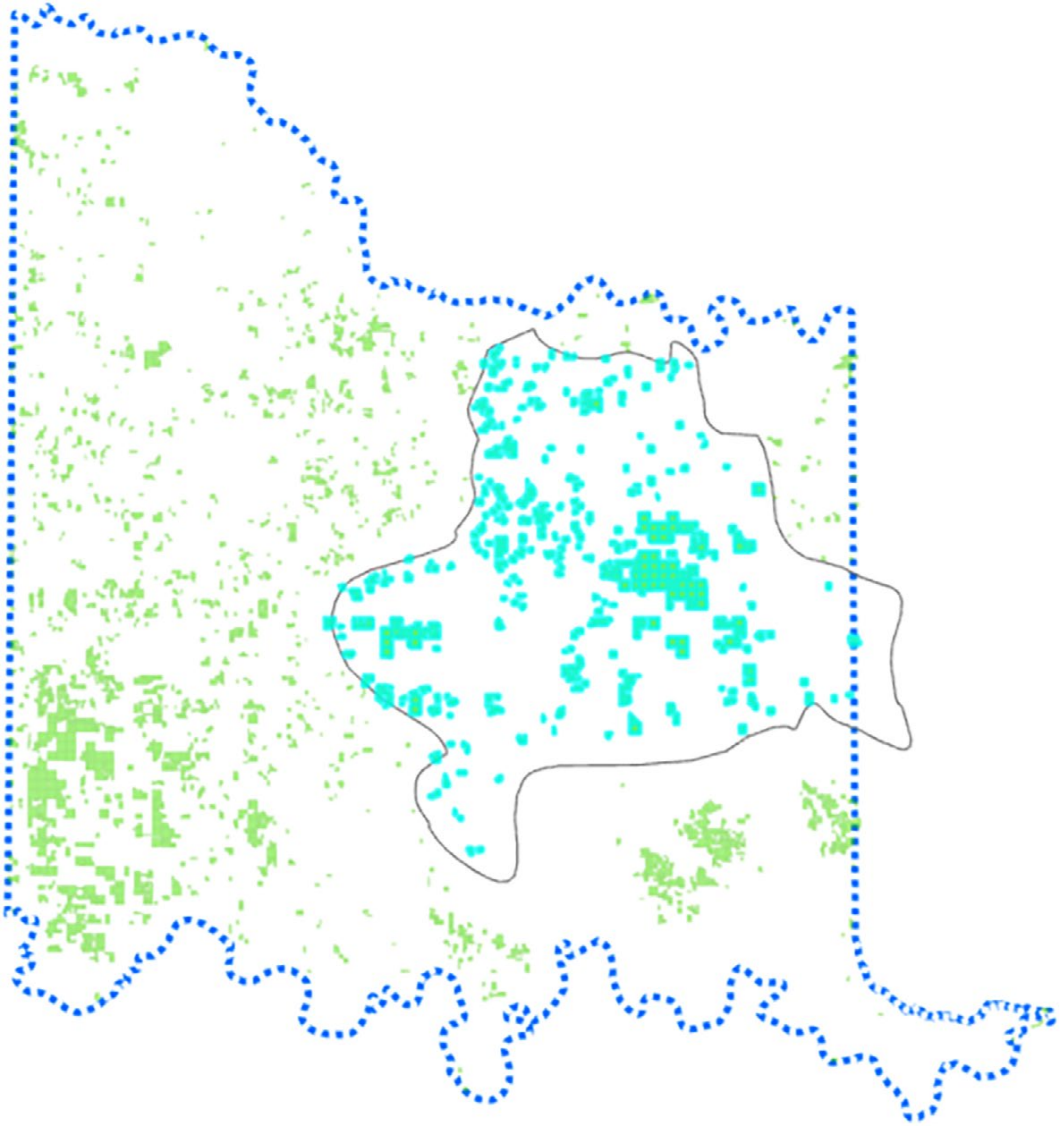
The Select by Attributes widget in the JET was used to perform a complex query in the Arbuckle Uplift major land resource area.

Lastly, the JET showed great potential when recently used to assist the NRCS in a complex query for a cost‐share program. Through the *Select by Attributes* widget, the JET identified all parcels larger than 40 acres, with greater than 50% intact area, and with less than 10% tree cover and woodland transition within the Arbuckle Uplift multiland resource area. The result of this analysis yielded 482 parcels and saved potentially hundreds of hours of office and field time (Figure 32).

### 6.5 Model Limitations

While the remotely sensed data and analyses described above provide a consistent and defensible estimate of juniper tree species distribution across the CNTT, there are some accuracy limitations associated with the model and data. In terms of spatial resolution, the first and last steps of the stepwise model (Table 2) used datasets that have a 10‐m spatial resolution (i.e., land cover and juniper identification). The land cover mask involved removing water areas and cropland and may have discarded groups of juniper tree species. Likewise, the final step of the model involved distinguishing between deciduous and coniferous trees using the Sentinel‐2 dataset which has a spatial resolution of 10 m; therefore, individual juniper trees, small stands and “edge” juniper trees may not be accurately classified due to pixel averaging (i.e., nonpure juniper pixels or “mixels”) of spectral reflectance data from other ground objects. Conversely, nonjuniper trees at or near the edges of juniper tree stands may also be misclassified as juniper trees due to the same pixel averaging effects.

Because the various LiDAR datasets used in this methodology were collected over a range of time periods (i.e., 2009 through 2018), stands of small trees currently present, but not evident in older LiDAR datasets, may not be classified correctly and multiple years of LiDAR data may capture tree canopy that no longer exists due to current management practices. These varying years of data created imperfect tree canopy data resulting in errors in classifying tree cover, and ultimately juniper cover. Unfortunately, LiDAR datasets are not updated frequently so the input in this step of the model is limited by the most up‐to‐date release; however, it was found that using the LiDAR dataset improved the model (i.e., over not using it at all). Lastly, for visual overlay purposes, the aerial base maps used in the JET may not have the same date stamp as the 2021–2022 data used in the model and could be misleading when interpreting the results displayed in the JET using the *Swipe Tool*.

It should be explicitly noted that the juniper tree species distribution model described in this article does not attempt to accurately map intact grassland areas but rather includes grasslands as part of the dispersal/recruitment and intact areas stages not heavily impacted by juniper species. Therefore, intact areas are not a surrogate for grasslands and may contain thick stands of deciduous trees (i.e., Cross‐Timbers vegetation) or individual juniper trees not captured by the model. An additional, related model could be produced to show grassland areas.

The land cover layer used in the model could be improved, perhaps by utilizing higher resolution base imagery layers; however this addition would be costly and much more time‐consuming on a large scale. Therefore, the highest resolution datasets available were used. Likewise, purchasing higher resolution leaf‐off imagery for the final step of the model (i.e., isolating juniper tree species) would offer an improvement over Sentinel‐2 imagery; however, this would again be cost prohibitive, especially for statewide and regional expansion. Similarly, higher resolution data could help to distinguish between different juniper species (e.g., Eastern Redcedar and Ashe Juniper).

In addition to tree cover and juniper species vulnerability provided by the JET, other data sets related to land features (i.e., water areas, crop land, roadways and urban areas) as well as attributes such as average parcel elevation (ft), average parcel slope (%), average parcel juniper height (ft) and median parcel juniper height (ft) (Figure 19) are provided. Despite its limited use on large parcel sizes, gridding the raster data, as opposed to tying the data to parcels, might prove a better option for more detailed planning, as elevation transects, slope, juniper height (especially for fire planning), and other ecological data could be used more effectively to make a sub‐parcel selection on the map canvas.

The JET is a static, region‐specific intrastate tool and is not updated “on the fly” when compared to other web‐based tools such as the RAP. If expanded to the state of Oklahoma, the JET would require updates every 2 years (i.e., the usual statewide NAIP leaf‐on data refresh schedule). In terms of historical data for Oklahoma, it would be possible to backdate the JET to 2015, the start of Sentinel‐2 data acquisition, but the other layers would also need to be backdated. Future versions of the JET in the CNTT may include ecological data for vulnerability refinement and/or a growth model to project juniper expansion in the future and assess the economic risk of “doing nothing.”

As for expansion outside of the state of Oklahoma (e.g., southern Great Plains), this would likely require a much larger team to process, analyze and display the modeled data. Although this can be done relatively easily using the thresholding and masking techniques described in this article, each state would have different release dates of LiDAR and NAIP data; this could be integrated into the JET using various state tabs. Also, different states may have areas where nonjuniper evergreen species exist, and these might be indistinguishable from juniper species. Depending on the objectives of each state, better classification methods (e.g., object‐oriented analysis) could be used to map woody encroachment, but this would require more time and funding.

## 7 Conclusions and Recommendations

Aligned with the program goals of the NRCS CIG program (Natural Resources Conservation Service, n.d.), this project is innovative in its use of recent open‐source satellite imagery and aerial photography, geospatial data and methods, and the GEE platform. By utilizing remote sensing processing techniques on source data sets within GEE, a public‐facing conservation planning web tool, called the JET, was created with the primary goal of enhancing the decision‐making for BMPs with data derived from a nonbiased, science‐based approach. A corollary to this goal was to put decision‐making in the hands of individual landowners, land managers and CN resource managers. This will enable the CN resource managers and partners to quickly and economically work toward restoring the productivity of agricultural ranchland and grasslands, reduce field survey and planning time, and focus on high‐priority areas for managing juniper tree species. The results of this project will also be used to help analyze and describe the economics associated with prescribed fire and encourage the targeted use of various removal methods within the CNTT.

Furthermore, where there is considerable interest from tribal and state governments in promoting the adoption and continued use of BMPs to manage juniper infestation, a methodology is needed to routinely evaluate juniper encroachment. This will allow: (1) tracking of juniper tree spread over time; (2) targeting areas in which removing and halting the spread of juniper species would prove most economical and effective; and (3) demonstrating the various positive effects of prescribed fire and other juniper removal methods for landowners.

At the federal level, the JET could add value and save time and money that would be otherwise allocated for mapping juniper vulnerability in the field with multiple trips to communicate planning efforts with landowners by USDA NRCS staff. It also provides visualization and science‐based information about juniper expansion and vulnerability on landowner property for specific programs (e.g., Environmental Quality Initiative Program [EQIP], Great Plains Grassland Initiative [GPGI], Working Lands for Wildlife, etc.) targeting invasive species management and providing cost‐share assistance (Oklahoma NRCS, n.d.; Conservation Coalition of Oklahoma n.d.; Fogarty et al. 2023; Natural Resources Conservation Service 2021; USDA NRCS 2017, 2022).

At the state level, the JET can assist in similar efforts championed by Oklahoma House Bill 2239 which created the Terry Peach Watershed Restoration Act (http://www.oklegislature.gov/BillInfo.aspx?Bill=HB2239&Session=2400) that allocated funding to the Oklahoma Conservation Commission for the purpose of collaborating with landowners along the North Canadian River to control the infestation of juniper species. At the local level, the JET provides stakeholders with science‐based knowledge necessary to make land management decisions based on juniper vulnerability and prevent further juniper encroachment. Understanding the changes in juniper tree species distribution within the CNTT could also help to tie changes in water quality and quantity to juniper removal and other best management practices moving forward. Potentially, impact (e.g., ecological and economic) over the years can be tracked as the progression of woody encroachment is slowed and landowners' grazeable lands increase while also protecting ecological integrity and economic productivity. This approach will help all stakeholders prioritize management practices and make constructive and financially responsible land management decisions. In the end, these local actions will help deliver positive impacts to CN waterways and watersheds and potentially provide a springboard for an expanded tool that can be applied in other parts of Oklahoma, the Great Plains, or nationally.

## Author Contributions

**Mark Micozzi:** conceptualization (equal), data curation (equal), formal analysis (equal), funding acquisition (lead), investigation (equal), methodology (equal), project administration (lead), supervision (lead), validation (equal), writing – original draft (lead), writing – review and editing (lead). **Justin Baker:** conceptualization (equal), data curation (equal), formal analysis (equal), investigation (equal), methodology (equal), supervision (supporting), validation (equal), writing – original draft (supporting), writing – review and editing (supporting).

## Funding

This work was supported by the Natural Resources Conservation Service, NR223A750013G024.

## Conflicts of Interest

The authors declare no conflicts of interest.

## Supporting information


**Data S1:** ece372551‐sup‐0001‐supinfo.docx.

## Data Availability

All data locations are either provided in the manuscript or in the references section. The public‐facing interactive decision support tool, called the Juniper Evaluation Tool (JET), contains the deliverables of the NRCS CIG funded project and is hosted by the Oka' Institute at East Central University in Ada, Oklahoma (see https://www.okainstitute.org/). Navigate to the Oka' Spatial/Interactive Maps tab at the top menu bar to open the JET. The direct link can be found here: https://the‐oka‐institute‐spatial‐data‐center‐okainstitute.hub.arcgis.com/apps/cbd35674f54b43afad8fed376e4eae6f/explore. A link to all the manuscript files (e.g., figures, non‐Google Earth Engine data sources, county and watershed JET data, RAP versus JET tree cover statistics, and the GEE code) can be found using this link for reviewers and the public: https://doi.org/10.5061/dryad.31zcrjf1s.

## Endnotes

^1^
While some areas of rocky outcrops and canyons may contain old growth juniper woodlands, these are not distinguished in this project.
