# Supplementary material for: A Decision Support Tool Using an Open‐Source Methodology for Identifying Woody Encroachment and Juniper Species Vulnerability in the Chickasaw Nation, Oklahoma, USA
Source: Ecol Evol. 2025 Dec 21;15(12):e72551. doi: 10.1002/ece3.72551 (PMC12719920; doi:10.1002/ece3.72551)
Supplement: Supplementary file 1 — Data S1: ece372551‐sup‐0001‐supinfo.docx. [file ECE3-15-e72551-s001.docx]

Supplemental Material

A Decision Support Tool Using an Open-Source Methodology for Identifying Woody Encroachment and Juniper Species Vulnerability in the Chickasaw Nation, Oklahoma, USA

Mark Micozzi, PhD. - Water Resources Planner. Office of Natural Resources. The Chickasaw Nation. Ada, Oklahoma, USA. mark.micozzi@chickasaw.net

Justin Baker, PE, CFM. - Water Resources Engineer. Hazen and Sawyer. Austin, Texas, USA. jcbaker@hazenandsawyer.com

**Study Area**

The CNTT encompasses all or part of 13 counties within south-central Oklahoma (Figure 1), covering approximately 7,648 square miles (4.9 million acres) of the Central Great Plains and Cross Timbers Ecoregion (Figure 2). Land use within the CNTT is primarily agricultural, though there are several municipal areas, including Ada, Ardmore, Sulphur, and Tishomingo. There are also several large water bodies, including the Lake of the Arbuckles, Lake Murray, and a large portion of Lake Texoma in the CNTT. However, approximately 80% of the land cover within the CN is made up of Grass/Pasture (approx. 53%) and Deciduous Forest (approx. 28%), as shown in Table 1. As displayed in Figure 3, most of the cropping agriculture is found in the western portion of the CNTT. In the eastern portion of the CNTT, most agriculture consists of cow-calf operations and ranching.


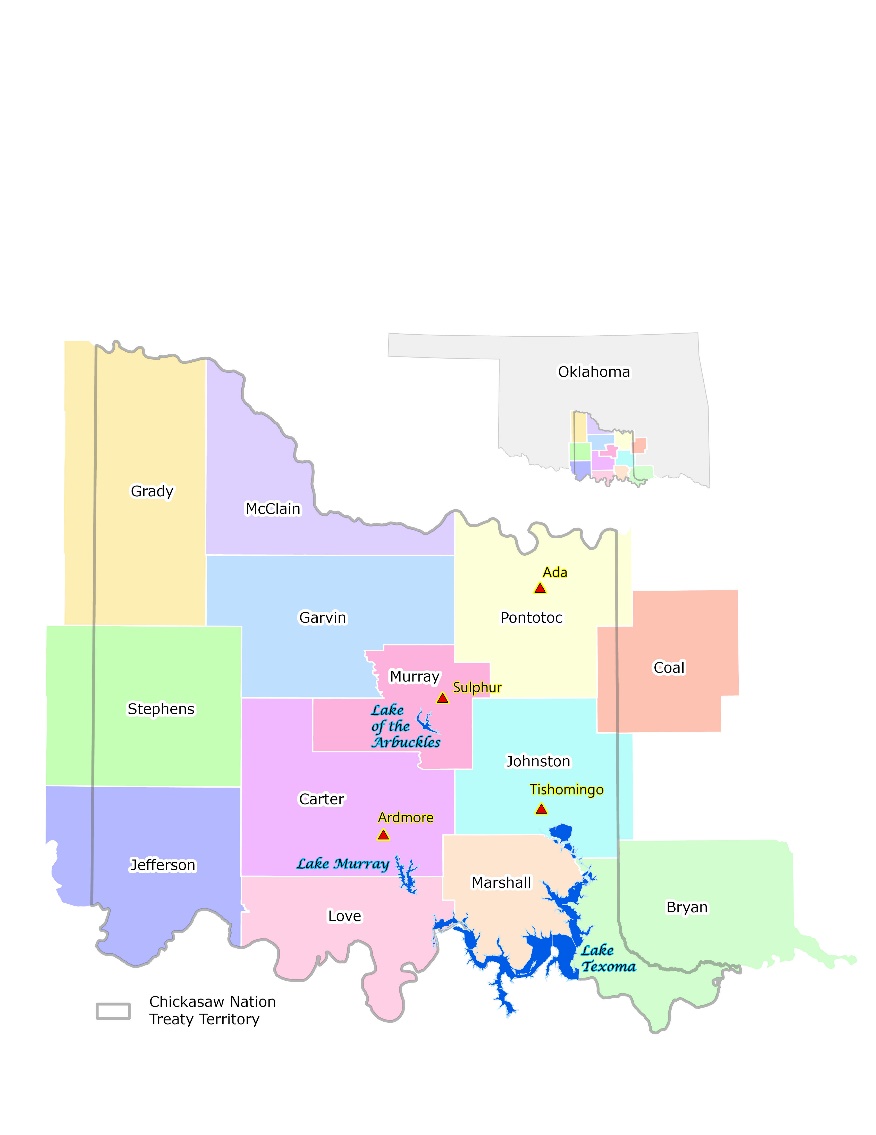


Figure 1: The Chickasaw Nation Treaty Territory and noteworthy towns and lakes. Tribal jurisdictional boundaries can be found on the Bureau of Indian Affairs website at https://onemap-bia-geospatial.hub.arcgis.com.


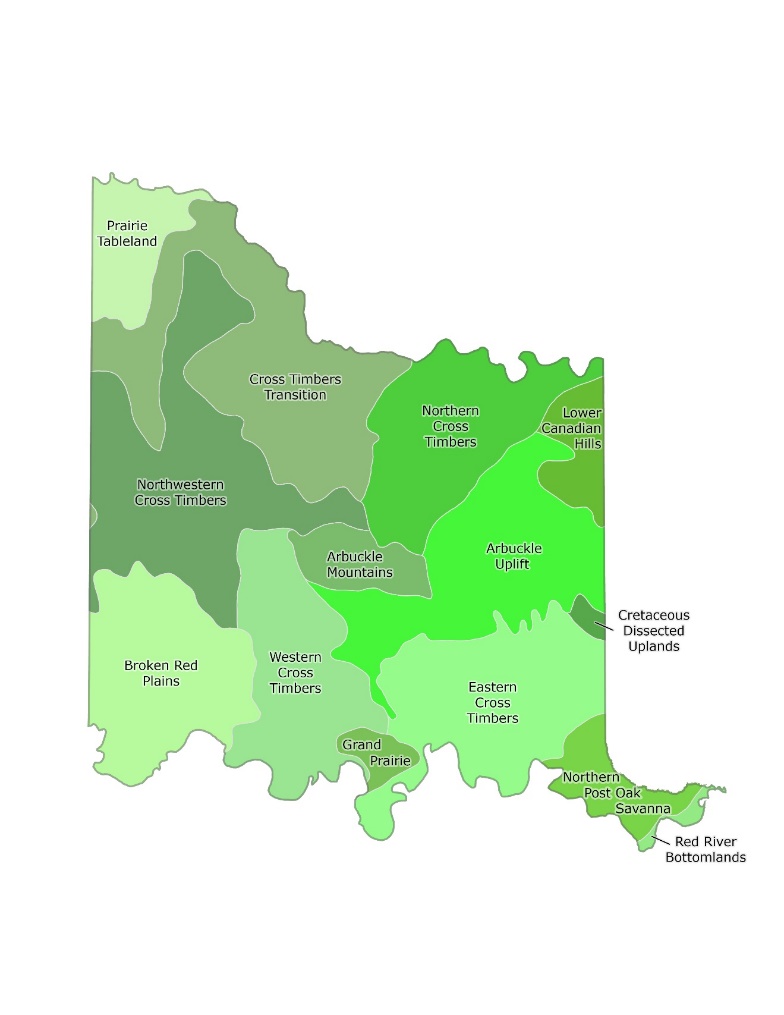


Figure 2: The Chickasaw Nation Treaty Territory within the Central Great Plains and Cross Timbers Ecoregions (members of each ecoregion shown). Source data: <https://www.epa.gov/eco-research/ecoregions>

Table 1: Land use classes within the Chickasaw Nation Treaty Territory. Landcover data are drawn from USDA’s Cropland Data Layer (2021), available here: <https://www.nass.usda.gov/Research_and_Science/Cropland/Release/>

| Land Use Class | Area (acres) | %  Cover | Land Use Class | Area (acres) | % Cover |
| --- | --- | --- | --- | --- | --- |
| Grass/Pasture | **2,518,000** | **52.9%** | Alfalfa | 32,000 | 0.7% |
| Deciduous Forest | **1,350,000** | **28.3%** | Evergreen Forest | 26,000 | 0.5% |
| Winter Wheat | 266,000 | 5.6% | Shrubland | 26,000 | 0.5% |
| Developed/Open Space | 139,000 | 2.9% | Corn | 23,000 | 0.5% |
| Open Water | 130,000 | 2.7% | Soybeans | 12,000 | 0.3% |
| Developed/Low Intensity | 66,000 | 1.4% | Barren | 12,000 | 0.3% |
| Other Hay/Non-Alfalfa | 58,000 | 1.2% | Developed/High Intensity | 12,000 | 0.2% |
| Developed/Med Intensity | 41,000 | 0.9% | All other land cover/use | 55,000 | 1.2% |

*
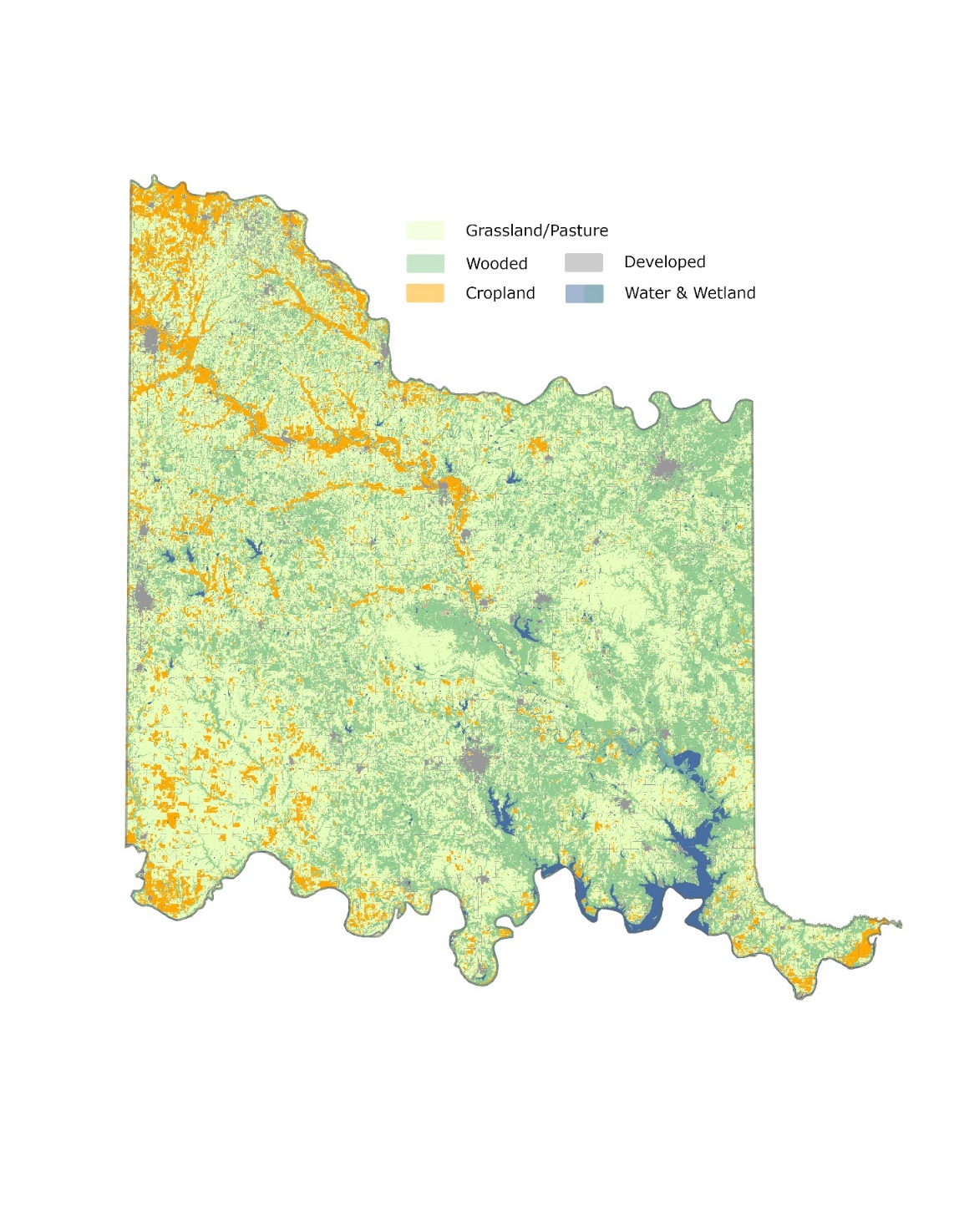
*

Figure 3: Land use in the Chickasaw Nation Treaty Territory. Landcover data are drawn from USDA’s Cropland Data Layer (2021), available here: <https://www.nass.usda.gov/Research_and_Science/Cropland/Release/>

**Open-Source Woody Encroachment Web-Based Tools**

There are several open-source tools worth mentioning that have been developed to help support large-scale vegetation cover mapping for rangeland management. The Rangeland Analysis Platform (RAP, n.d.), known as the RAP tool and now managed by the USDA Agricultural Research Service (ARS) (USDA Agricultural Research Service 2023), was created in 2018 by the University of Montana in partnership with Working Lands for Wildlife of the USDA (NRCS, ARS) and the Department of the Interior (BLM, NPS), and utilizes field-collected data and 30-meter Landsat satellite imagery. Its primary purpose is to provide a free, user-friendly, public, science-based geospatial tool that is multi-decadal, nation-wide, cloud-powered and offers near-real time updates for assessing trends in rangeland resources by mapping rangeland vegetation cover and production, such as annual and perennial forbs and grasses, shrubs, trees, and bare ground dating back to 1985. (RAP n.d., USDA-NRCS Working Lands for Wildlife n.d., USDA Agricultural Research Service 2023).

The Sage Grouse Initiative (SGI) Interactive Web Application (Ricca et al. 2018, Sage Grouse Initiative n.d.), launched in February 2016, is a free, user-friendly and public science-driven geospatial tool developed by USDA–NRCS in partnership with the University of Montana and others that combines layers of related data to better target invasive species damaging sagebrush-steppe habitat and rangeland across 11 western U.S. states (Working Lands for Wildlife, n.d.). The tool primarily uses object-based image analysis and automated feature extraction applied to high-resolution NAIP aerial photography. This process allows for the detailed mapping of pinyon-juniper presence, distribution, and canopy cover while integrating ecosystem resilience and resistance indices at one-meter spatial resolution. The methodology yields highly accurate outputs which are rigorously validated through reference imagery and ground-based surveys with strong classification accuracy, enabling mapping of both individual trees and broader woodland canopies (Gustafson et al. 2018, Roth et al. 2021). The combined efforts of sequential work along the way (Poznanovic et al. 2014, Falkowski et al. 2017, Coates et al. 2017, Ricca et al. 2020) have resulted in a robust, replicable, and scalable conservation planning tool that overcomes the shortcomings of previous lower-resolution products. It delivers highly precise, actionable data to land managers, facilitating targeted, efficient, and evidence-based strategies for sage-grouse habitat protection and broader rangeland conservation goals. This tool represents a critical advance for conservation planning in sagebrush ecosystems, providing both the scientific foundation and practical resources for effective, large-scale management of pinyon-juniper expansion and sage-grouse habitat conservation.

The Multi-Resolution Land Characteristics (MRLC) Consortium, a partnership among federal agencies including the U.S. Geological Survey (USGS) and U.S. Department of Agriculture (USDA), develops and maintains two complementary national geospatial products: the National Land Cover Database (NLCD) and the Rangeland Condition Monitoring, Assessment, and Projection (RCMAP) dataset (Multi-Resolution Land Characteristics Consortium n.d.). The NLCD, first released in 1992 and regularly updated, provides comprehensive nation-wide land cover, land cover change, and tree canopy data at 30-meter resolution, enabling consistent monitoring of urban growth, forest trends, water surface changes, and land use patterns (Housman et al. 2023, Jin et al. 2023). The tree canopy data (i.e., the Tree Canopy Cover project) uses Landsat and Sentinel-2 imagery, along with USDA Forest Service Forest Inventory and Analysis data to create tree canopy estimates for the continental United States.

The RCMAP dataset and tool form a remote sensing-based system developed to track, analyze, and project the conditions of rangelands across the western United States dating back to 1985. RCMAP quantifies the percent cover of eight key rangeland components (annual and perennial herbaceous, bare ground, litter, non-sagebrush shrub, sagebrush, total shrub, and tree canopy) by integrating extensive field data and 30-meter Landsat satellite imagery. Advanced machine learning models, particularly neural networks, were used to predict fractional cover and detect both gradual and abrupt changes to the landscape. The tool also provides detailed annual timeseries maps and trend analyses and projects future vegetation cover under different climate scenarios. Widely validated using multiple independent field datasets, RCMAP supports land managers and researchers in monitoring vegetation dynamics, assessing management actions, appraising habitat health and fragmentation, and making data-driven conservation decisions at various spatial and temporal scales. All RCMAP products are openly accessible and free, facilitating transparent, timely, and science-based rangeland management, making it a powerful tool for adaptive management across broad landscapes (Rigge et al. 2022, Shi et al. 2022, Rigge 2023, Multi-Resolution Land Characteristics Consortium n.d.)

The Rangeland Brush Estimation Tool (RaBET), developed by the USDA ARS and made publicly available in 2023 (Hollifield Collins et al. 2023), in collaboration with the NRCS under the Conservation Effects Assessment Project (CEAP) for Grazing Lands, is a free, user-friendly and public science-based geospatial tool designed to quantify woody plant encroachment across western rangelands in the United States. By integrating 30-meter Landsat satellite imagery with high-resolution aerial photography (NAIP) and ground-based measurements, RaBET provides repeatable, field-scale estimates of brush canopy cover. The tool allows landowners, conservation planners, and natural resource managers to evaluate current brush density, assess historical changes, efficiently monitor brush removal and its reemergence, and monitor the effectiveness of land management practices, facilitating informed resource allocation for conservation practices. By offering a spatially and temporally dynamic view of woody vegetation trends, RaBET supports data-driven decision-making for targeted brush control, ecological restoration, and rangeland productivity improvement across diverse landscapes (Holifield Collins et al. 2015, Holifield Collins et al. 2023, Rangeland Brush Estimation Toolbox n.d., USDA-Agricultural Research Service 2025).

**Tree Cover Distribution Estimates**

Although tree cover was not the main output of the JET, it deserves discussion as it was essential for mapping juniper extent. While other methods and products exist for generating tree cover, they either lack the simplicity or level of positional accuracy that the JET’s stepwise model provides. For instance, a comparison was made between the tree cover canopy classification results from the RAP (Rangeland Analysis Platform n.d.) and the tree canopy layer from the JET model for all counties and the overall study area (Table 2 and Figure 4). Tree canopy cover estimates derived from the RAP and the JET differed significantly across the 13-county comparison (paired t-test, t(12) = –3.87, *p* = 0.002). On average, the JET reported higher tree cover percentages than the RAP and this may reflect differences in data sources and dates, pixel averaging and spatial resolution, masking techniques, and classification methods used by each platform.

Table 2: Percent tree cover (2021-2022) from the RAP and JET and percent of JET tree cover above or below the RAP tree cover estimates by county in the CNTT. Data for the RAP was obtained from <https://rangelands.app/> using shapefiles uploaded for each county.

| **County** | **2021-2022 Tree Cover** | | **Percent of JET Tree Cover Above (+) or Below (-) RAP Tree Cover Percent** |
| --- | --- | --- | --- |
|  | **RAP (% Area)** | **JET (% Area)** |  |
| Garvin | 23.64 | 22.54 | -1.10 |
| Bryan | 32.73 | 32.06 | -0.66 |
| Marshall | 35.08 | 35.63 | +0.56 |
| Grady | 17.43 | 18.68 | +1.26 |
| Jefferson | 14.19 | 16.16 | +1.97 |
| Stephens | 23.25 | 25.85 | +2.60 |
| Pontotoc | 34.47 | 37.9 | +3.44 |
| Love | 32.87 | 36.41 | +3.54 |
| McClain | 21.70 | 25.93 | +4.23 |
| Carter | 27.86 | 32.8 | +4.95 |
| Murray | 25.72 | 32.85 | +7.13 |
| Johnston | 32.24 | 40.96 | +8.73 |
| Coal | 41.46 | 50.35 | +8.90 |
| CNTT | 26.48 | 29.14 | +2.67 |


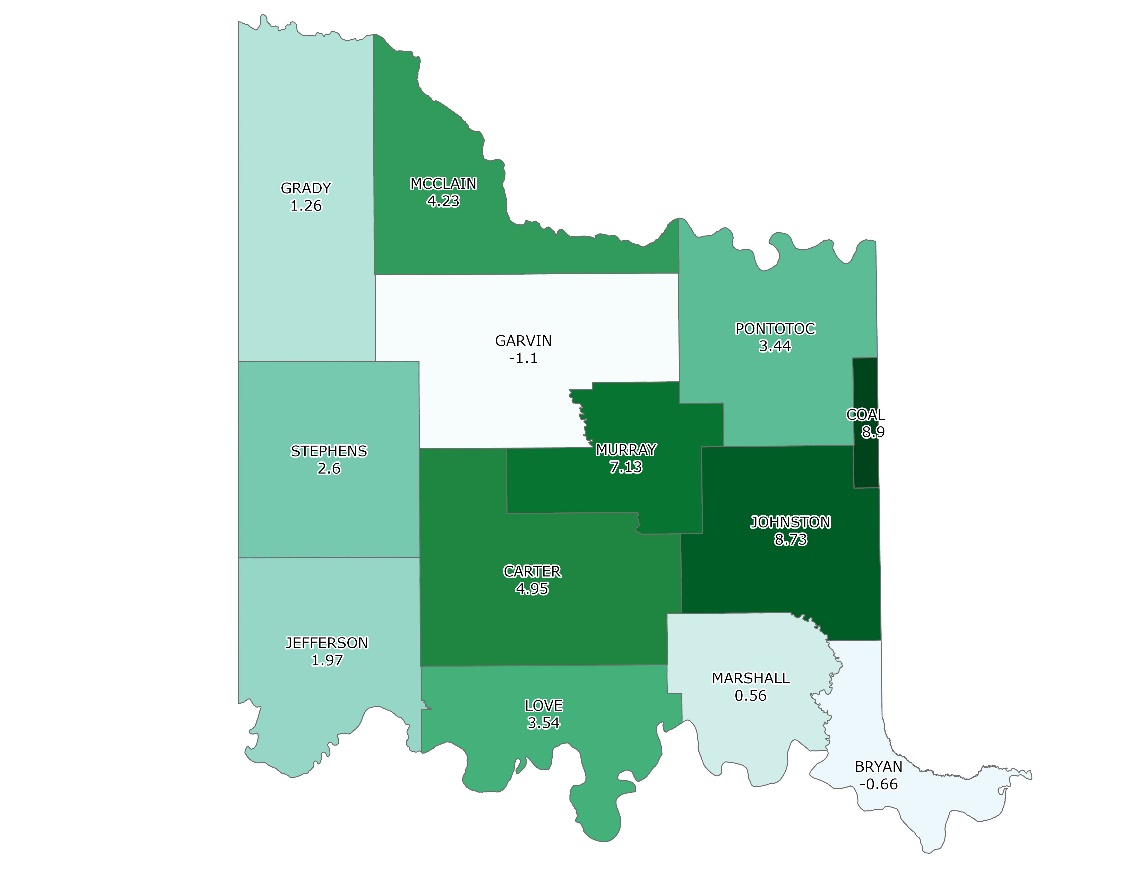


Figure 4: Percent of JET tree cover above or below the RAP tree cover percent by county.

Another comparison was made against the Tree Canoy Cover (TCC) dataset, a part of the National Land Cover Database (Multi-Resolution Land Characteristics Consortium n.d.), which calculates a TCC value using photo interpretation methods and machine learning models. Both the TCC and RAP use a classification gradation scale of 0-100 (lighter to darker pixel color ramp) for each 30-meter pixel to estimate the percentage of pixel area covered by tree canopy, while the JET model uses a presence/absence classification. A visual representation (Figure 5) shows an example of the finer resolution and presence of tree cover of the JET output, versus the courser gradation scale of the RAP and TCC datasets. In the images shown in Figure 6, there is a higher level of tree detail and accuracy in the JET tree cover (purple transparent color) and this coincides with the location of the varying intensities of the courser shades of green-gray pixel symbology of the TCC dataset. Graphic (a) shows the level of detail of the JET tree cover over the TCC dataset. Graphic (b) shows the varying pixel levels of the TCC corresponding to the location of the JET tree cover. Graphics (c) and (d) show a larger-scale view of both datasets and the level of detail in the JET tree cover. An unimproved pasture road is seen in (d) as green with tree cover lining both sides of the roads. This reinforces the importance of having accurate juniper tree cover location data over a courser gradation scale (i.e., percent of a pixel) method.

The Forestry Services Division of the Oklahoma Department of Agriculture, Food, and Forestry, the primary state agency responsible for managing, protecting, and enhancing Oklahoma's forest resources, produced high-quality forest cover maps (Johnson et al. 2010), as did the Center for Earth Observation and Modeling at the University of Oklahoma (Yao et al. 2025), but since the resolution (i.e., 30 meters) was similar to previous datasets, they were not compared. For the purposes of this project, it was important to produce accurate tree canopy cover data to subsequently produce the woodland transition vulnerability layer, and therefore the use of a lower-resolution tree canopy dataset, such as that available from the RAP or MRLC, was precluded.


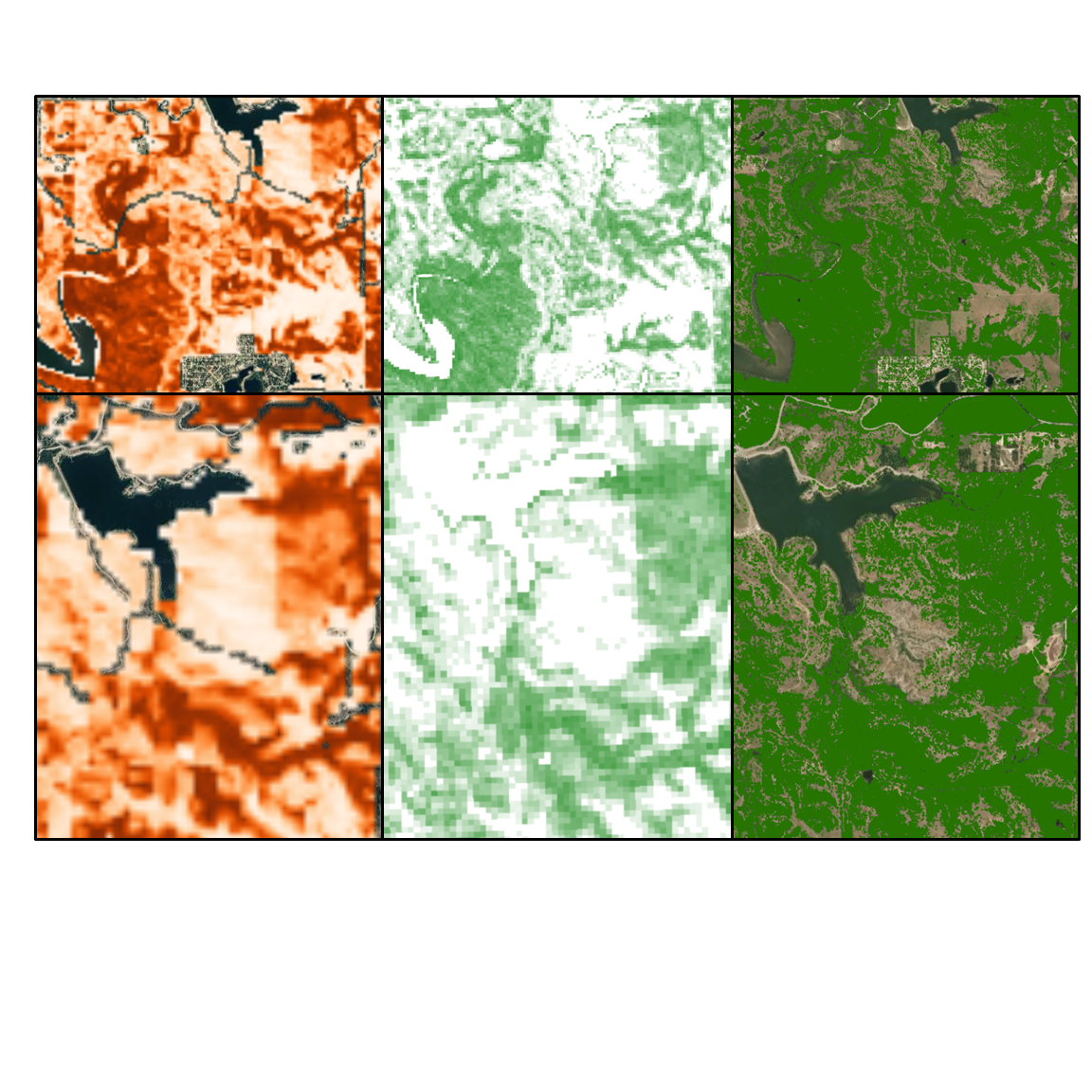


Figure 5: Tree cover data from (a) the RAP tool (left), (b) the NLCD (center) and (c) the JET (right). Varying shades of orange (RAP) and green (NLCD) represent percentages (0-100) of tree cover within a pixel. Lighter shades represent less tree cover percent within a pixel and darker shades represent more. The dark green color of the JET represents tree cover canopy at a higher spatial resolution.

Figure 6: Tree canopy data from the JET (transparent purple) on top of the tree canopy cover dataset from the NLCD in varying shades of green. Darker areas of green are the higher percentage pixels of the tree canopy cover dataset from the NLCD that also intersect with the transparent tree canopy JET data.


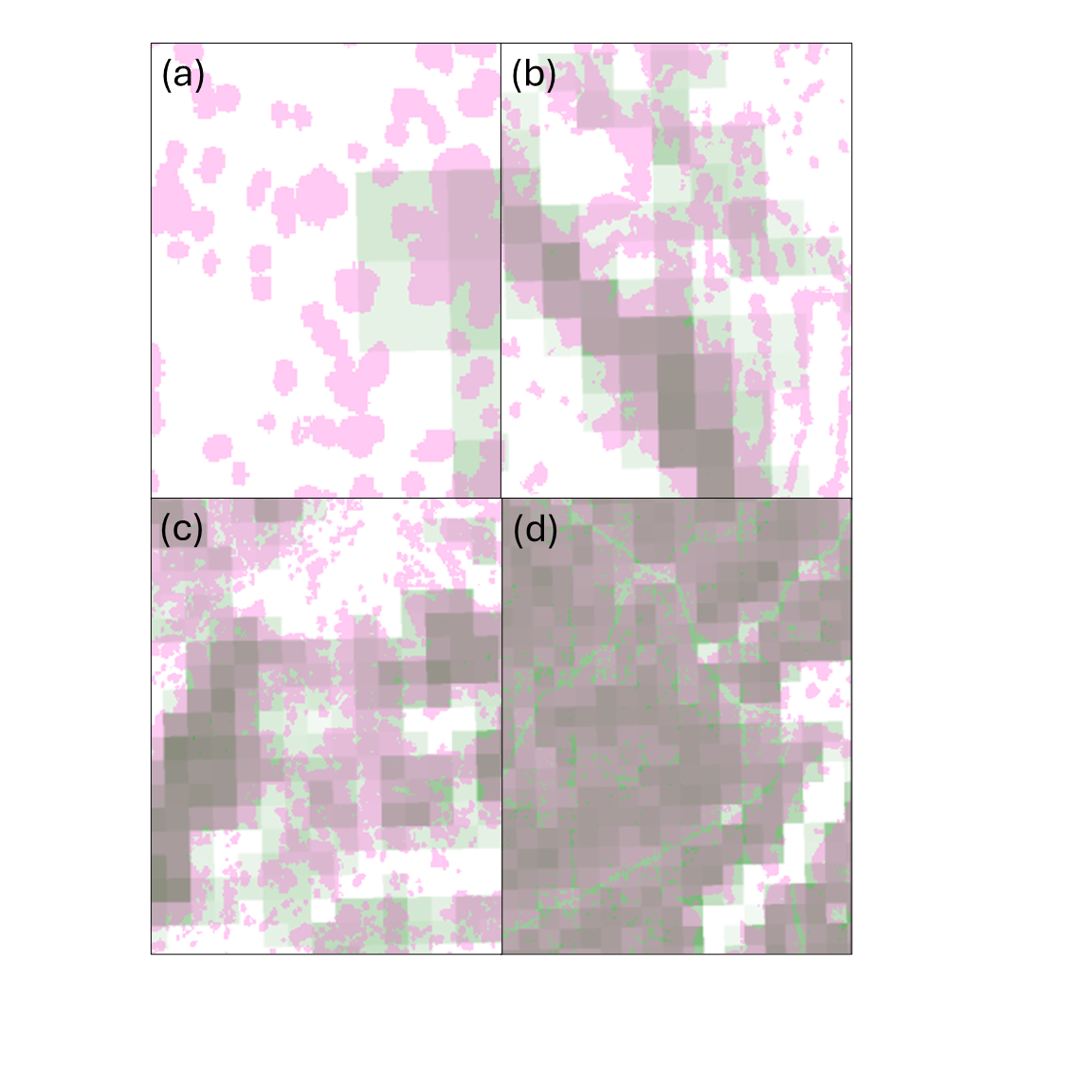


**References**

Coates, P.S., Gustafson, K.B., Roth, C.L., Chenaille, M.P., Ricca, M.A., Mauch, Kimberly, Sanchez-Chopitea, Erika, Kroger, T.J., Perry, W.M., and M. L. Casazza. 2017. Geospatial data for object-based high-resolution classification of conifers within the geographic range of the Bi-State Distinct Population Segment of greater sage-grouse in California and Nevada: U.S. Geological Survey data release. https://doi.org/10.5066/F7G15ZRN.

Falkowski, M.J., Evans, J.S., Naugle, D.E., Hagen, C.A., Carleton, S.A., Maestas, J.D., Khalyani, A.H., Poznanoic, A.J., and A.J. Lawrence. 2017. Mapping tree canopy cover in support of proactive prairie grouse conservation in western North America. Rangeland Ecology & Management. 70:15-24. <https://doi.org/10.1016/j.rama.2016.08.002>.

Gustafson, K.B., Coates, P.S., Roth, C.L., Chenaille, M.P., Ricca, M.A., Sanchez-Chopitea, E., and M.L. Casazza. 2018. Using object-based image analysis to conduct high- resolution conifer extraction at regional spatial scales. International Journal of Applied Earth Observation and Geoinformation. 73: 148-155. <https://doi.org/10.1016/j.jag.2018.06.002>.

Holifield Collins, C., Skirvin, S., Kautz, M., Winston, Z., Curley, D., Corrales, A., Bishop, A., Bishop, N., Norton, C., Ponce-Campos, G., Armendariz, G., Metz, L., Heilman, P., and W. van Leeuwen. 2023. Rangeland Brush Estimation Tool (RaBET): An Operational Remote Sensing-Based Application for Quantifying Woody Cover on Western Rangelands. Remote Sensing. 15(21): 5102.

Holifield Collins, C.D.; Kautz, M.A.; Tiller, R.; Lohani, S.; Ponce-Campos, G.; Hottenstein, J. and L. J. Metz. 2015. Development of an integrated multiplatform approach for assessing brush management conservation efforts in semiarid rangelands. Journal of Applied Remote Sensing. 9: 096057.

Housman, I. W., Schleeweis, K., Heyer, J. P., Ruefenacht, B., Bender, S., Megown, K., Goetz, W., and S. Bogle. 2023. National Land Cover Database Tree Canopy Cover Methods v2021.4 (GTAC-10268-RPT1). U.S. Department of Agriculture, Forest Service, Geospatial Technology and Applications Center. <https://data.fs.usda.gov/geodata/rastergateway/treecanopycover/index.php>.

Jin, S., Dewitz, J. A., Danielson, P., Granneman, B. J., Costello, C., Smith, K., and Z. Zhu. 2023, National Land Cover Database 2019—A new strategy for creating clean leaf-on and leaf-off Landsat composite images: Journal of Remote Sensing. 3: 0022. <https://doi.org/10.34133/remotesensing.0022>.

Johnson, E., Geissler, G., and D. Murray. 2010. Oklahoma Forest Resource Assessment: A comprehensive analysis of forest-related conditions, trends, threats and opportunities. Oklahoma Forestry Services, Oklahoma Department of Agriculture, Food, and Forestry. [https://www.forestry.ok.gov](https://www.forestry.ok.gov/).

Multi-Resolution Land Characteristics Consortium (MLRC). n.d. <https://www.mrlc.gov/> and <https://www.mrlc.gov/viewer/>. Last accessed July 22, 2025.

Poznanovic. A. J., Falkowski, M. J., Maclean, A. L., Smith, A. M. S. and J. S. Evans. 2014. An Accuracy Assessment of Tree Detection Algorithms in Juniper Woodlands. Photogrammetric Engineering & Remote Sensing. 80(5): 627–637. doi: 10.14358/PERS.80.7.627.

Rangeland Analysis Platform (RAP). n.d. <https://rangelands.app/>. Last accessed August 1, 2025.

Rangeland Brush Estimation Toolbox (RaBET). n.d. <https://www.tucson.ars.ag.gov/rabet/>. Last accessed February 20, 2025.

Ricca, M. A., Coates, P. S., Gustafson, K.B., Brussee, B. E., Chambers, J. C., Espinosa, S. P., Gardner, S. C., Lisius, S., Ziegler, P., Delehanty, D. J. and M. L. Casazza. 2018. A conservation planning tool for Greater Sage-grouse using indices of species distribution, resilience, and resistance. Ecological Applications. 28: 878-896. <https://doi.org/10.1002/eap.1690>.

Ricca, M. A. and P. S. Coates. 2020. Integrating Ecosystem Resilience and Resistance Into Decision Support Tools for Multi-Scale Population Management of a Sagebrush Indicator Species. Frontiers in Ecology and Evolution. 7. <https://doi.org/10.3389/fevo.2019.00493>.

Rigge, M. 2023. Rangeland Condition Monitoring Assessment and Projection, 1985–2021. USGS Fact Sheet 2023-2024. <https://doi.org/10.3133/fs20233004>

Rigge, M. B., Bunde, B., Postma, K., and H. Shi. 2022. Rangeland Condition Monitoring Assessment and Projection (RCMAP) fractional component time-series across the western U.S. 1985–2021: U.S. Geological Survey data release. Last accessed July 5, 2025. <https://doi.org/10.5066/P9ODAZHC>.

Roth, C.L., Coates, P.S., Gustafson, K.B., Chenaille, M.P., Ricca, M.A., Sanchez-Chopitea, E. and M.L. Casazza. 2021. A customized framework for regional classification of conifers using automated feature extraction, MethodsX. 8: 101379. <https://doi.org/10.1016/j.mex.2021.101379>.

Shi, H., Rigge, M., Postma, K., and B. Bunde. 2022, Trends analysis of Rangeland Condition Monitoring Assessment and Projection (RCMAP) fractional component time series (1985–2020). GIScience & Remote Sensing. 59(1): 1243–1265.

Sage Grouse Initiative (SGI). n.d. <https://map.sagegrouseinitiative.com/ecosystem?ll=43.4799,-110.7624>. Last accessed July 5, 2025.

USDA Agricultural Research Service. 2023. USDA-ARS Now Stewarding the Rangeland Analysis Platform. <https://www.ars.usda.gov/news-events/news/research-news/2023/usda-ars-now-stewarding-the-rangeland-analysis-platform/>.

USDA, Agricultural Research Service. 2025. RaBET Development for Rangelands in the Western U.S. (Project No. 2022-13610-013-014-S). Southwest Watershed Research Center. Retrieved on August 15, 2025 from <https://www.ars.usda.gov/research/project/?accnNo=440844&utm_source>.

USDA-NRCS, Working Lands for Wildlife, n.d. Rangeland Analysis Platform: Monitoring Rangelands Across the United States [Fact sheet]. Retrieved from [www.wlfw.org](http://www.wlfw.org).

Working Lands for Wildlife. n.d. Keeping Trees Where They Belong. <https://www.wlfw.org/landscapes/sagebrush/woodland-expansion/>. Last assessed June 21, 2025.

Yao, Y., Xiao, X., Qin, Y., Wang, J., Zhang, C., Newman, G., Pan, L., Meng, C., Pan, B. and C. Yin*.* 2025. Improved annual forest cover maps in Oklahoma from analyses of PALSAR-2, Landsat, and LiDAR data sets during 2015–2021. Frontiers of Earth Science. 19: 304–321 <https://doi.org/10.1007/s11707-025-1151-4>.
